# Super-Resolution Imaging of Voltages in the Interior of Individual, Vital Mitochondria

**DOI:** 10.1021/acsnano.3c02768

**Published:** 2023-06-08

**Authors:** ChiaHung Lee, Douglas C. Wallace, Peter J. Burke

**Affiliations:** ^†^Department of Electrical Engineering and Computer Science, ^‡^Department of Biomedical Engineering, University of California, Irvine, California 92697, United States; Δ Center for Mitochondrial and Epigenomic Medicine, Children’s Hospital of Philadelphia and Department of Pediatrics, Division of Human Genetics, University of Pennsylvania, Philadelphia, Pennsylvania 19104, United States

**Keywords:** Voltage, mitochondria, super-resolution, fluorescent dye, metabolism, electrophysiology, lipid bilayer

## Abstract

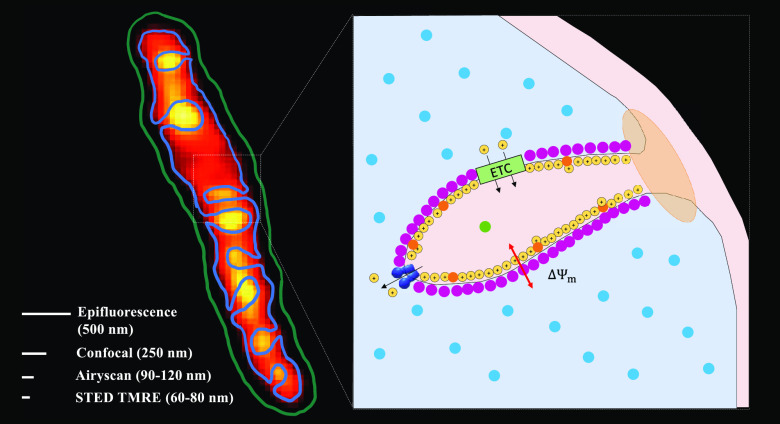

We present super-resolution microscopy of isolated functional mitochondria, enabling real-time studies of structure and function (voltages) in response to pharmacological manipulation. Changes in mitochondrial membrane potential as a function of time and position can be imaged in different metabolic states (not possible in whole cells), created by the addition of substrates and inhibitors of the electron transport chain, enabled by the isolation of vital mitochondria. By careful analysis of structure dyes and voltage dyes (lipophilic cations), we demonstrate that most of the fluorescent signal seen from voltage dyes is due to membrane bound dyes, and develop a model for the membrane potential dependence of the fluorescence contrast for the case of super-resolution imaging, and how it relates to membrane potential. This permits direct analysis of mitochondrial structure and function (voltage) of isolated, individual mitochondria as well as submitochondrial structures in the functional, intact state, a major advance in super-resolution studies of living organelles.

Analysis of mitochondrial structure and function is increasingly being recognized as central to understanding human health and disease.^1−6^ Yet mitochondria within tissue cells can have markedly different structures and functions. Hence, there is a critical need to be able to characterize the structure and function of the individual isolated mitochondrion. One of the most important mitochondrial functions is the generation of cellular ATP by oxidative phosphorylation (OXPHOS). OXPHOS consists of the electron transport chain (ETC) plus the ATP synthase. The four multisubunit enzyme complexes of the mitochondrial inner membrane ETC (complexes I–IV) oxidize hydrogen derived from carbohydrates and fats with oxygen to generate H_2_O. Starting with NADH for complex I or succinate for complex II, the electrons are transferred to coenzyme Q, then complex III, through cytochrome c to complex IV, and then on to oxygen. As the electrons transverse complex I, III, and IV, protons derived from H_2_O (H^+^ + OH^–^) are transported out of the mitochondrial matrix to create an electrochemical gradient that is negative and alkali in the matrix and positive and acidic on the opposite side of the inner membrane. Thus, the electrochemical gradient ΔP consists of a membrane potential (ΔΨ_m_), also called voltage, and a pH gradient (Δμ^H+^) with the pH gradient typically less significant: ΔP = ΔΨ_m_ + Δμ^H+^. The mitochondria maintain a membrane electrochemical potential of about 150 mV.^7^ This electrochemical gradient (ΔP) is a source of potential energy for multiple mitochondrial functions, the most important being the driving of the ATP synthase (complex V) to condense ADP + P_i_ to generate ATP.

Respiration of isolated mitochondria is commonly studied by the sequential addition of substrates and specific respiratory complex inhibitors. NADH-linked substrates such as pyruvate and glutamate feed electrons through complex I while succinate feeds electrons into complex II. The addition of these substrates results in electron transport, increased ΔP, and oxygen consumption known as state II respiration. The addition of ADP activates complex V to deplete ΔP and increase the rate of oxygen consumption known as state III respiration. Depletion of the ADP returns the respiration to the pre-ADP state known as state IV respiration. The addition of a compound such as carbonyl cyanide *m*-chlorophenylhydrazon (CCCP) depletes the electrochemical gradient resulting in maximal “uncoupled” respiration. The different steps of OXPHOS can be blocked using specific OXPHOS complex inhibitors including rotenone for complex I, antimycin A for complex III, and oligomycin for complex V.

The mitochondrial inner membrane is highly in-folded into double membrane structures known as cristae. These cristae are closed at the intersection with the intermembrane space between the inner and outer mitochondrial membrane to create closed cristae lumens.^8^ The ETC pumps the protons into the cristae lumens on which the ATP synthases are bound.^9,10^ Cleavage of Optic atrophy-1 (OPA1), which is located at the site where the cristae lumen is closed, releases the protons and disrupts the coupling between the ETC and ATP synthase.^8^ The mitochondrial system is important for multiple other mitochondrial functions including mitochondrial membrane dynamics, thermogenesis, Ca^2+^ homeostasis, redox signaling, and apoptosis.^11,12^

Mitochondria ultrastructure has been extensively characterized with transmission electron microscopy (TEM) and CryoTEM in fixed cells.^13−18^ However, only by imaging functional, intact mitochondria can one ascertain information about the electrophysiology of the organelle. Because the cristae are about 100 nm wide, they cannot be studied with diffraction-limited microscopy. Recently, this limitation is being addressed using super-resolution microscopy.

Super-resolution optical microscopy of fixed cells has been deployed extensively to study cristae structure and membrane protein distributions along the membrane.^19^ But to understand the physiology of individual mitochondria, super-resolution of functional, intact mitochondria is required. Recent studies have employed super-resolution microscopy on mitochondria in live cells.^20−237^ To put this work into proper context, most of the previous works have focused on structure, not function (i.e., voltage). Although they are intimately linked, function is much more important (and difficult to measure) because, after all, life is function, not structure. Only one of these papers studied mitochondrial electrophysiology (function) using a membrane sensitive lipophilic cationic dye on whole cells, proving the concept of live cell super resolution imaging of voltages in mitochondria. Our work builds on the outstanding and pioneering prior work presented in ref (20), enhancing and clarifying the biophysical model of the interpretation of voltage microscopy, which, together with the invention of super-resolution microscopy, allows us contribute to the biophysics of this emerging field. A lipophilic cationic dye diffuses freely from one side of a membrane to the other, since the hydrophobic side groups give it good solubility in the interior of the lipid bilayer. No channel or energy consumption or redemption is required to allow passage of the dye. At low concentrations, the charge does not affect the membrane voltage, and the dye accumulates on one side or the other because thermodynamics says the probability of a particle being in a given state is proportional to e^∧^–(energy/*kT*) of that state. Therefore, the ratio of dye concentrations is given by the Nernst equation, discussed in more detail below. However, many previous studies^34^ incorrectly assumed all the dye was free, neglecting the membrane binding of the dye, which has long been known to be the most significant component of the total dye fluorescence.^24^ Reference (20) identified membrane bound dye, but did not consider it as pertinent to the fluorescence of energized mitochondria. We show in this paper through detailed experimental analysis and modeling that the membrane bound dye constitutes over 90% of the fluorescent signal in energized mitochondria. For example, our studies in 2010^25,26^ on isolated mitochondria using tetraphenyl phosphonium lipophilic cation (TPP^+^), detected electronically (rather than optically as in this study), used a calibration routine to convert to membrane potential that assumed (based on work by Kamo^27^ others) that the total amount of TPP^+^ on the matrix side was approximately 90% bound to the inner membrane, and 10% free. Other groups later^28^ used the same procedure, which lumped the membrane binding into an “activity coefficient” that combined free and membrane bound dye since they could not (until now) be resolved at that time. We^29^ and others (e.g.,^30^) that previously used diffraction-limited microscopy to image voltage dye concentration (Tetramethylrhodamine, Ethyl Ester, TMRE) were not able to resolve the cristae and therefore did not have any images showing membrane localization.

To characterize differences in the physiology of individual mitochondria from cells, we report here procedures for characterizing the mitochondrial structure and function of isolated, functional mitochondria using super-resolution microscopy. This revealed that we can use super-resolution quantification of mitochondrial membrane potential using lipophilic cationic dye fluorescence to characterize the respiratory function of individual isolated, functional mitochondria in a way not possible in whole cells, potentially permitting elucidation of differences between individual mitochondria. Finally, by careful analysis of structure dyes and voltage dyes (lipophilic cations), we demonstrate that most of the fluorescent signal seen from voltage dyes is due to membrane bound dyes, and develop a model for the membrane potential dependence of the fluorescence contrast for the case of super-resolution imaging, and how it relates to membrane potential. This quantitative voltage-dependent membrane binding model explains why super-resolution images of lipophilic cationic dyes show strong intensity near the cristae and not in the matrix. This model enables quantitative imaging of super-resolution voltages inside functional, intact mitochondria with super-resolution.

## Logical Structure of the Paper

This paper is organized into three major sections. In the first section, we develop and validate a quantitative model to demonstrate voltage imaging with super-resolution microscopy. In the second major section, we apply this to mitochondria in different metabolic states. In the third section, we discuss the need for biophysical models of mitochondrial function based on the Poisson statistics that results from the # of hydrogen ions in the cristae.

In the first section, we demonstrate that vital, isolated mitochondria voltages and ultrastructure can be imaged with super-resolution microscopy. Next, we demonstrate that the voltage dye is not free in the mitochondrial matrix but rather bound to the membranes, albeit in a voltage dependent manner. This finding contradicts prior, incorrect interpretations in some of the literature assuming that the dye is free and unbound, and requires a model to interpret the voltage imaging since the old model is wrong. Next, we develop and apply a model originally developed for diffraction limited micrsocopy (where the entire organelle appeared as one voxel, and the imaging technology could not localize the voltage dye within the organelle), and use this to quantitatively interpret and explain the voltage images we obtained. This model completely and consistently takes into account membrane binding. Next, in order to test our proposed model to more conditions, we use the model to make predictions about what would be observed in the absence of a membrane potential (e.g., created by CCCP) and to predict the fluorescence intensity inside the matrix of the small amount of free (unbound) voltage dye. These are unusual conditions which researchers usually do not investigate in detail. Sure enough, our measurements in these unusual conditions completely confirm the predictions of our voltage dependent membrane binding model. With this comprehensive, quantitative, experimentally validated model, we have provided the foundation for all future studies on the voltage distribution in mitochondria measured with super-resolution microscopy.

In our studies, we used all 3 modern methods of super-resolution microscopy: Airy, STED, and Lattice SIM (Structured Illumination Microscopy). While it is possible in some methods to observe clearly the outline of internal structure of mitochondria such as individual cristae, with voltage imaging, the resolution (of order 90 nm) is only able to observe cristae if they are separated, but cannot observe individual cristae in cristae dense mitochondria. We discuss this quantitatively in this section and demonstrate how the density of cristae, their voltage distribution, and the response to pharmacological manipulation of different metabolic states can be observed, even if individual cristae are not resolved.

The second major part of this paper aims to apply this model to mitochondria in different metabolic states, something not possible in whole cells. This demonstrates super-resolution voltage imaging in mitochondria in different metabolic states. These findings both reproduce all of the classical biochemistry metabolic studies of mitochondria, as well as demonstrate changes in the spatial distribution of the voltages as a function of metabolic state.

In the third section, based on our observed experimental data in the first two sections, we model the proton distribution inside the cristae semiquantitatively based on the membrane potential, capacitance, and pH. We demonstrate that bulk models of pH need to be replaced by refined models of electrophysiology at the nanoscale in mitochondria.

## Results

### Vital isolated mitochondria cristae structures and super-resolution electrical voltages can be resolved by super-resolution microscopy

**Figure 1:**
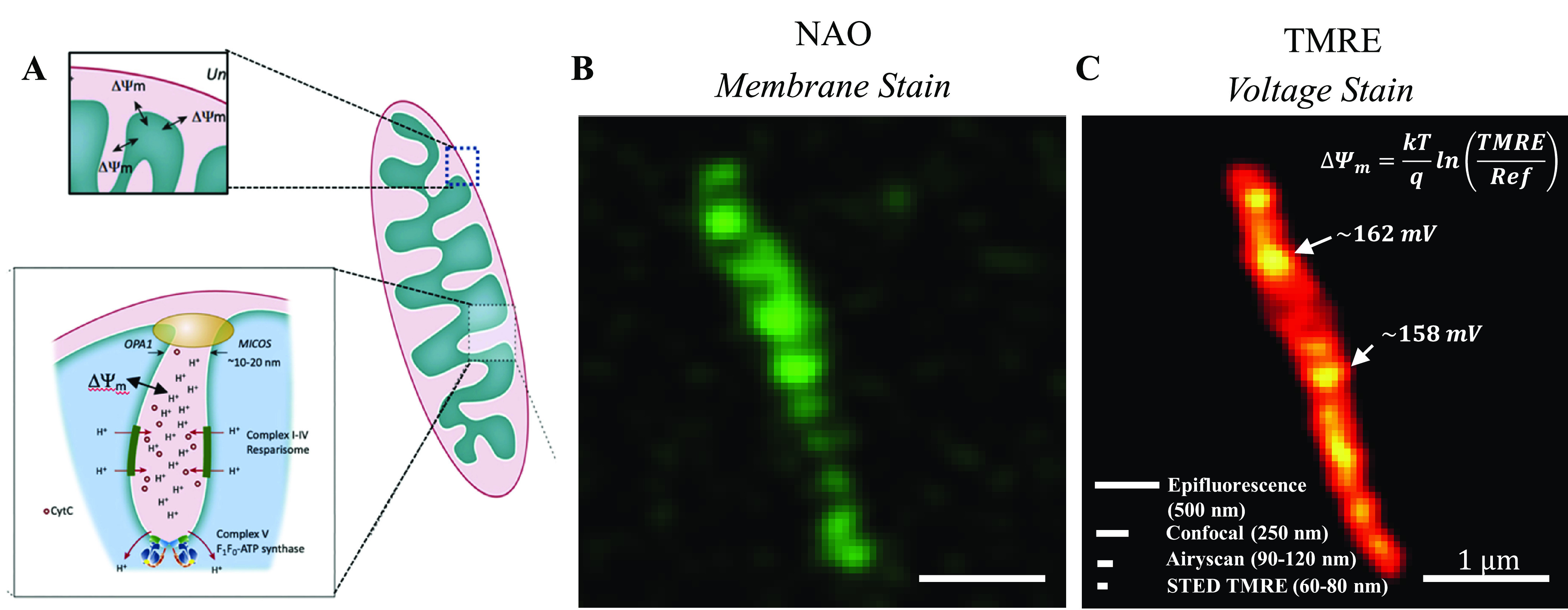
Model and images of isolated mitochondria. (a) Model of mitochondrial membrane voltage.^10^ (b) Isolated mitochondria stained with membrane fluorescent dye, NAO (c) Isolated mitochondria stained with voltage-dependent dye, TMRE. A simple application of the Nernst equation. Scale bars show the resolution of various imaging technologies for the TMRE dye. Reprinted from Trends Cancer, 3 (12), Burke, P. J. Mitochondria, Bioenergetics and Apoptosis in Cancer, Pages 857–870. Copyright (2017). with permission from Elsevier.

The ultrastructure of the organelle can be imaged using membrane binding dyes such as Nonyl Acridine Orange (NAO), mitotracker green (MTG), mitotracker red (MTR), mitotracker deep red (MTDR), *etc*. In this work, we utilize these dyes to image the location of the membrane cristae within the limits of the resolution of the imaging system. (Although these dyes show some dependence on membrane potential,^31^ we only use Tetramethylrhodamine, Ethyl Ester (TMRE) for voltage imaging, as it is more studied, and better understood.) This is shown in Figure 1, where we have used super-resolution microscopy (Airyscan) with NAO to image the ultrastructure of a single mitochondrion isolated from a human cell line (Methods).

In order to quantitatively assay membrane potential, a lipophilic cationic dye is traditionally used. As the dye has lipophilic moieties, it is soluble in the hydrophobic interior of the lipid bilayer membrane, and can readily diffuse from one side to the other. Since it is charged, its location inside vs outside the mitochondria is dependent on the membrane potential ΔΨ_m_. Motivated by the apparent success of this approach in previous literature,^7,32,33^ we seek to attain additional information about the spatial dependence of ΔΨ_m_ within the organelle. In Figure 1C, we show the fluorescence intensity of the lipophilic cationic dye TMRE in an isolated mitochondrion. The cristae harboring the ETC transported protons are clearly giving rise to a nonuniform distribution of TMRE, and hence a very nonuniform voltage ΔΨ_m_, along the organelle. Also shown in Figure 1C are scale bars indicating the spatial resolution of various imaging technologies. Thus, using the Airyscan microscope, we have succeeded in imaging the membrane potential of vital, isolated mitochondria.

How does one interpret these images in terms of the actual membrane potential/voltage and the TMRE dye? If one neglects binding to the membrane itself (discussed below), the difference in densities across the membrane would be governed by the Nernst equation:
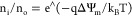
1where ΔΨ_m_ is the membrane voltage, and n_i_, n_o_ are the lipophilic cationic dye concentrations inside and outside the mitochondria (respectively), indirectly measured via the fluorescence intensity. A simplified application of the Nernst equation, using the background fluorescence (n_o_), indicates varying voltages (ΔΨ_m_) along the mitochondria, near where the cristae are, indicating “high” voltages near the cristae. In addition, large dark regions seem to indicate low voltages in the mitochondrial matrix.

However, this simple interpretation does not consider membrane bound dye as pertinent to the fluorescence of energized mitochondria. We show next in this paper through detailed experimental analysis and modeling that the membrane bound dye (which has long been known to be a significant factor^24^) constitutes over 90% of the fluorescent signal in energized mitochondria.

### Most of the voltage sensitive cationic lipophilic dye TMRE is membrane bound, not free

**Figure 2:**
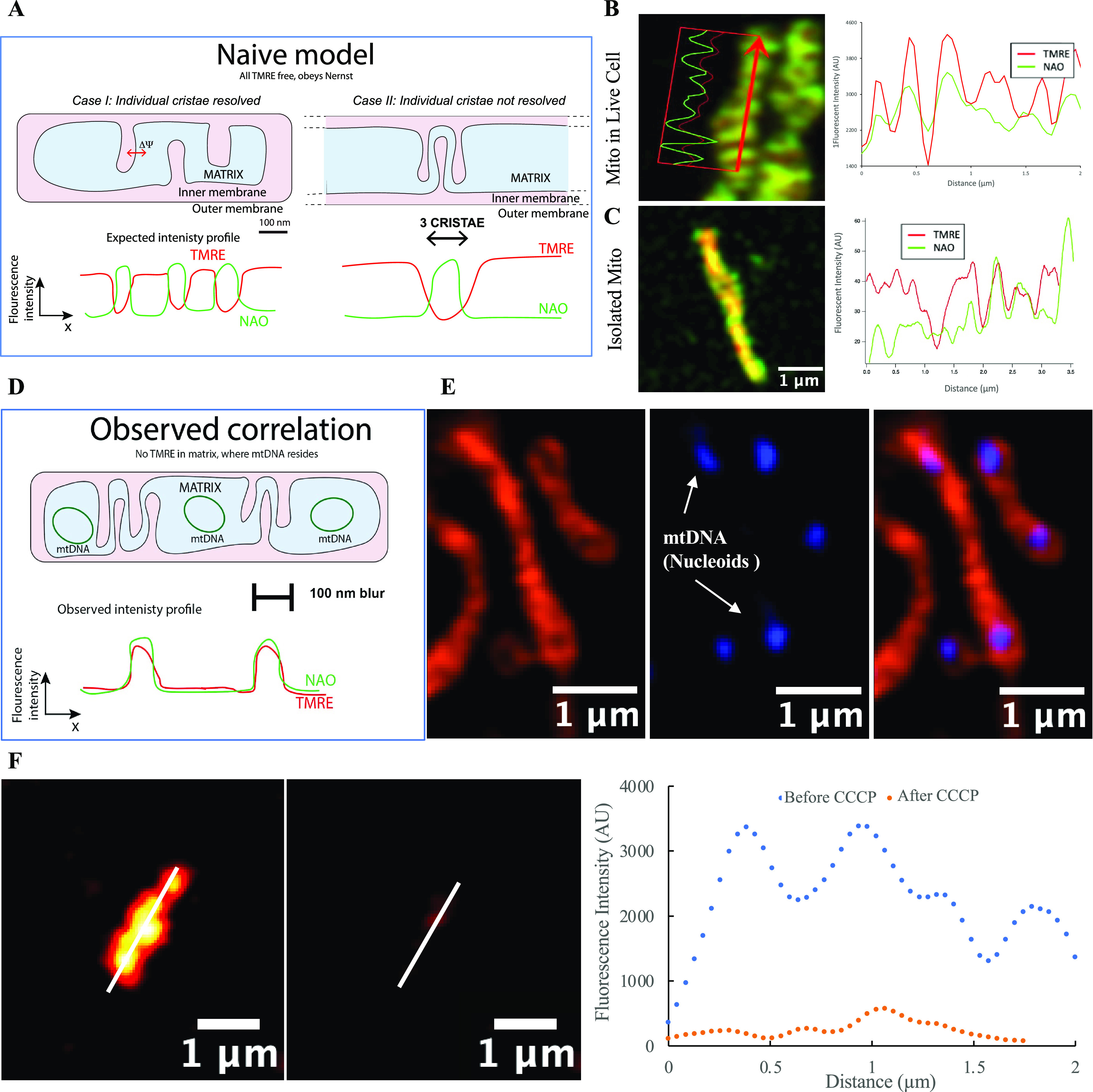
Correlation of TMRE and NAO. (A) Naïve model of TMRE uptake, neglecting membrane binding. If TMRE only exists as free molecules in the matrix, responding to the membrane potential ΔΨ_m_ according to the Nernst equation (eq 1), then the TMRE intensity should be anticorrelated with the membrane dye NAO, in contradiction to what we observed. Line profile of (B) mitochondria in a HeLa cell and (C) isolated mitochondria, stained with TMRE and NAO. The voltage sensitive cationic dye TMRE is localized at the cristae membrane, as demonstrated by the correlation between the TMRE and NAO intensity peaks, in contradiction to the naïve model for free TMRE. (D, E) Summary of experimental observations of TMRE localization in mitochondria. mtDNA was shown to be in the dark regions through PicoGreen staining in a separate experiment. (F) Line profile of isolated mitochondria TMRE image before and after treating with 10 μM CCCP.

Our Airyscan images reveal that the TMRE fluorescence correlates with the distribution of the mitochondrial cristae, as detected by the cardiolipin binding dye NAO. This is confirmed by plotting a line scan of the intensity along the length of individual vital mitochondria showing the overlap between the TMRE and NAO fluorescence levels (Figure 2B and Figure 2C). We observe this in both whole cells (reproducing ref (20), which found the same), as well as isolated mitochondria. Given that the mitochondrial inner membrane thickness is 4 nm and in tightly coupled mitochondria the in-folded membranes are tightly juxtaposed with a separation between 20 and 100 nm, we can estimate that a cristae finger would have a width of approximately 30 to 110 nm which is within the spatial resolution of the Airyscan microscope of 90–120 nm. Hence, this places the positively charged lipophilic dye in close proximity to the positive electromagnetic field of the cristae, an anomaly observed in a previous Airyscan study of mitochondria within the living cell.^20^

To further confirm that the TMRE is not evenly distributed within the mitochondrial matrix, we stained the isolated mitochondria with PicoGreen which stains mitochondrial nucleoids green. Mitochondrial nucleoids are located in the mitochondrial matrix (Figure 2D), and the staining of isolated mitochondria with TMRE and PicoGreen confirmed that the TMRE fluorescence does not correlate with the position of the mtDNA nucleoids and thus does not occupy the mitochondrial matrix (Figure 2E).

The explanation for this observation is that the TMRE is bound to the membrane, something which has been known with indirect studies for a long time.^24^ Here, voltage-dependent membrane bound lipophilic cationic dyes are being imaged in vital, isolated, intact, functional mitochondria, clearly and directly confirming via super-resolution microscopy that most of the TMRE are bound to the membrane, something that was only possible to determine via indirect methods previously.

### Binding of the TMRE Dye to the Membrane Model

**Figure 3:**
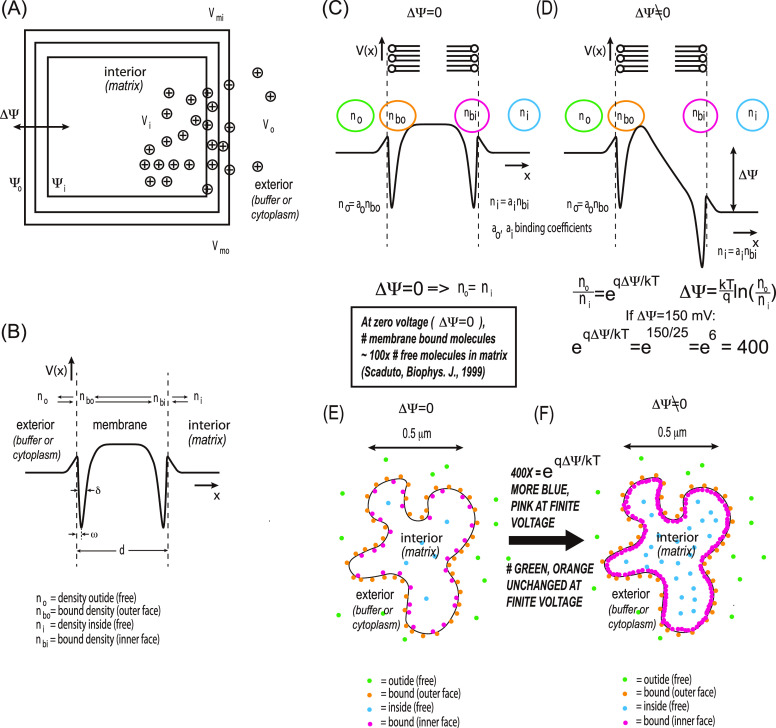
Uptake model for TMRE staining of mitochondria for voltage imaging. (A) Four-compartment model, showing membrane binding on both sides as well as the inside and outside. (B) Potential profile. (C, D) Zero and finite voltage potential profile of the binding of TMRE. The green and blue circles show the free TMRE on the outside (buffer side) and inside (matrix side) of the mitochondria. The orange and red circles show the bound TMRE on the outside and inside of the mitochondria, respectively. (E, F) Zero and finite voltage localization of the TMRE, showing the difference in # of TMRE molecules in the different regions. At finite voltage, the most intense fluorescence would be expected from the TMRE molecules indicated by the pink dots, localized at the matrix side of the membrane. The other TMRE molecules (free in the matrix, free in the buffer, bound to the buffer side) would be much dimmer and possibly not even observed within the practical dynamic range limits of the fluorescence detection system used. At zero voltage, the overall intensity (averaged over the organelle) would be much lower, but the intensity distribution would still be expected to be highest near the membrane.

In order to properly interpret the images of the TMRE intensity related to the membrane potential, it is critical to include the bound TMRE in consideration. To do this, we use a model proposed by Rottenberg in 1984,^35^ and shown in Figure 3A. Rottenberg^35^ proposed this in the context of mitochondria, but this model has not been used to interpret super-resolution images until this work: “*The* accumulation *of these cations by mitochondria is described by an uptake and binding to the matrix face of the inner membrane in addition to the binding to the cytosolic face of the inner membrane.*” Rottenberg used radiolabeled lipophilic cations (triphenylmethylphosphonium (TMTP^+^), tetraphenylarsonium (TPA^+^), and tetraphenylphosphonium (TPP^+^)) to determine the concentration in the buffer, and therefore no imaging was used. Kamo and Demura^27^ used this model and detected TPP^+^ in the buffer with electrochemical sensors; again no imaging was used: “*The membrane potential-dependent binding was analyzed with a model: the membrane is split into two halves, outer and inner half, and the amounts of bound probes in each region are governed by the concentration in the contiguous solution.*” Follow on work by Scaduto^24^ used TMRE and imaging, but not super-resolution imaging. We used diffraction-limited imaging also in mitochondria contained in nanofluidic chambers.^29^ In this work, we extend the use of this model to interpret voltage images using super-resolution imaging in functional, intact, isolated mitochondria and live cells.

In this model, TMRE is bound on the matrix and buffer (cytosol) side of the inner membrane. The amount of bound TMRE on the matrix side is proportional to the free concentration on the matrix side. The amount of bound TMRE on the buffer (cytosol) side is proportional to the free TMRE concentration on the buffer (cytosol) side. At zero membrane potential, the inner and outer free concentrations are equal. At nonzero membrane potential, the inner to outer free concentration obeys the Nernst equation; i.e., there is a higher inner concentration. Therefore, there is also a higher amount of TMRE bound on the inner membrane. The amount of TMRE bound on the outer membrane stays the same. It has been known since 1999^24^ that the amount of bound TMRE in the mitochondrial inner membrane is about 100× larger than the amount of free TMRE in the matrix (by mole #), regardless of the potential. *Since super-resolution imaging can resolve the cristae, i.e. internal membrane structure, it is mostly the TMRE bound on the inner membrane that is imaged during fluorescence, as it is larger by mole # than the free TMRE in the matrix, and as it is more concentrated (on the surface vs diffuse) in space.*

The biophysical basis of this model is shown in Figure 3. In this model, TMRE ions experience an effective potential energy profile (for zero voltage) shown in Figure 3B (from Rottenberg^35^). The shape of this curve results from the competition between the lipophilic side groups encouraging insertion into the membrane, and the charge encouraging repulsion from the membrane.^35^ Since both effects do not have the same spatial profile, this results in two minima in the potential, one at each side of the membrane, and thus two binding sites. The depth and width of the binding sites will determine the concentration of lipophilic cations in the membrane vs the free concentration: The concentration of free (unbound) ions in solution is proportional to the concentration of bound ions on the surface of the membrane. These constants of proportionality (defined as a_o_ and a_i_ in Figure 3) must typically be measured empirically (see Supporting Information (SI) section 3).

In Figure 3C,D this biophysical model is redrawn under zero and finite membrane potential, showing the TMRE densities bound to each side of the membrane, and free on each side of the membrane, and defining the binding coefficients (also referred to as activities in literature). We prefer the term binding coefficients instead of “activities” sometimes used in the literature, because it explicitly clearly states the physical meaning in this context. Assessing the location of TMRE is essential. However, various indirect techniques have been demonstrated to quantify the membrane binding in this model (see SI section 3). Although Figure 3C,D is implicitly what has been used to interpret membrane potential assays on mitochondria in the literature,^25−28^ it has never been explicitly presented as we have done in Figure 3C,D. The reason is that, until the advent of super-resolution microscopy, it was not possible to image the bound and free TMRE components independently.

Using known rate constants/activity coefficients (see SI section 3), we show in Figure 3E,F schematically how the TMRE molecules would be distributed in space at zero and finite membrane potential in a hypothetical mitochondrial inner membrane. Under finite potential, the purple population (TMRE bound to the inner membrane) will create the most fluorescence intensity. The free TMRE in the matrix (blue population) will be relatively dim. This is exactly what we observe experimentally (Figure 2). In sum because the # of TMRE molecules bound to the matrix side of the membrane is ∼100× larger than the number of free TMRE molecules inside the matrix, and because the membrane bound TMRE molecules are more concentrated on the surface of the membrane, it is the membrane bound TMRE on the inner membrane that “glows” in fluorescence images. This explains why, in our images, the cristae regions glow with TMRE but the matrix regions seem dark. A detailed accounting of the binding constants is presented in SI section 3.

Application of this model makes two additional predictions that we can test quantitatively: (1) The matrix region should not be completely dark, and (2) the membranes should still be labeled with TMRE even when the membrane potential is completely collapsed. We discuss these next.

### TMRE stains the membrane even after the voltage is collapsed

**Figure 4:**
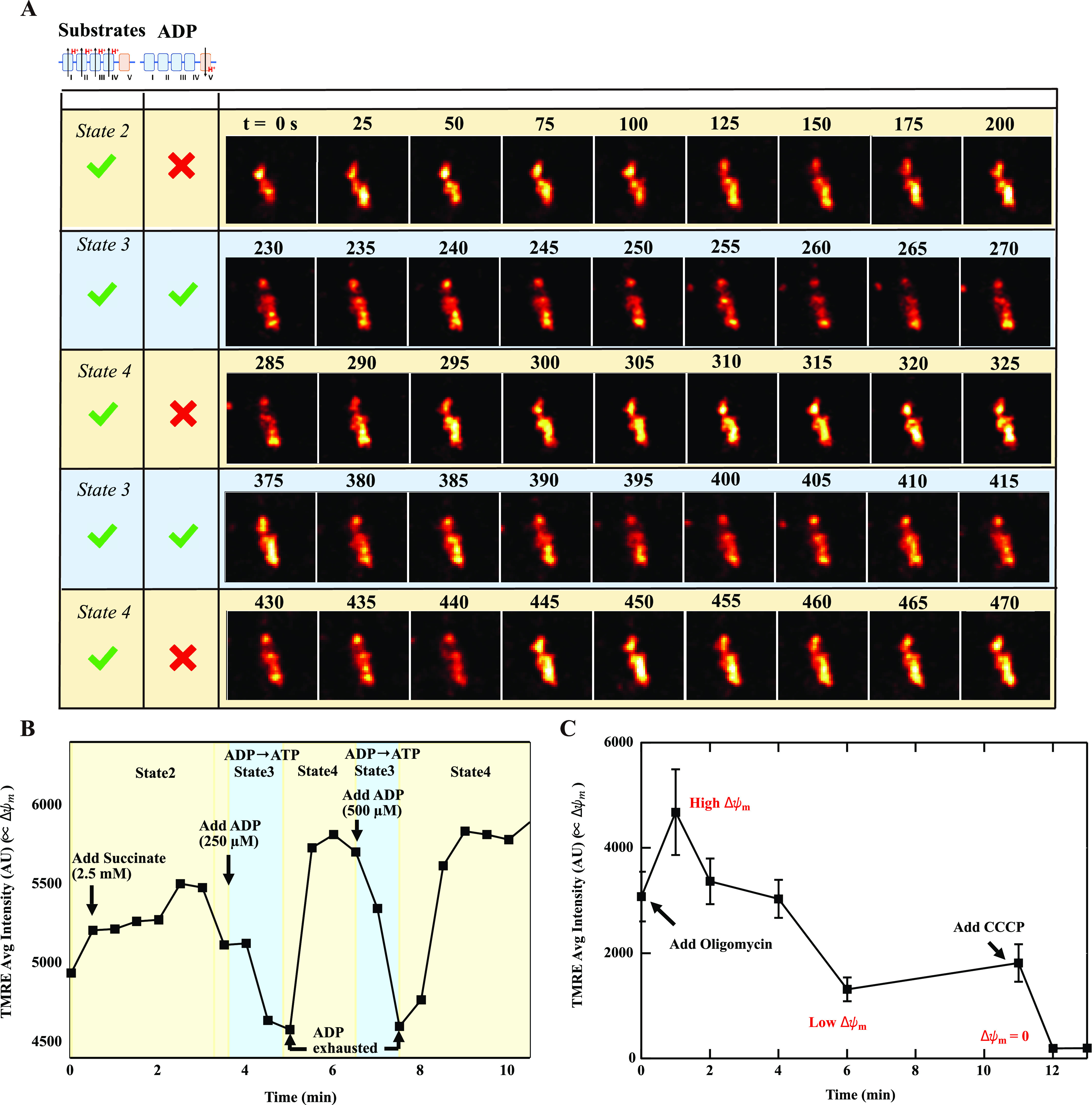
Isolated mitochondria TMRE signal in different respiratory states. (A) Mitochondrial structure in different respiratory states. The numbers on top of the figure represent the time in seconds. The pixel size of the image was 0.048 μm, which is limited by the inherent limits of the microscope. (B) Averaged TMRE changes of single mitochondrion during respiratory state transitions. (C) Averaged TMRE changes of single mitochondrion treated with Oligomycin and CCCP. Starting with 178 μg/mL isolated mitochondria, quantified by the Bradford assay, we stained the mitochondria with 10 nM TMRE and collected images using time-steps of 5 s to avoid photobleaching. The succinate (2.5 mM) was added at the 10th second, and ADP was added at the 240th second and again at the 380th second, giving a final concentration of 250 μM and 500 μM, respectively.

When we collapsed the voltage using CCCP, the total intensity of TMRE averaged over the entire organelle seemingly dropped to zero (Figure 4C and Figure 2F). However, on closer investigation, we found that it did not completely drop (Figure 2G). In the arbitrary units used, quantitatively it dropped to 200 when it was 5000 under energized mitochondria. Using super-resolution microscopy, we could look closer at the distribution of the dye after the collapse of the membrane potential. This is shown in Figure 2F. Although the overall intensity is lower, the shape is still similar. The line profile confirms the cristae are still labeled with (bound) TMRE, albeit at a much lower intensity. Thus, prior studies which showed the TMRE collapse are consistent with this work. In addition, this confirms the application of the model we proposed above. These effects are reproducible in different cell lines (see SI section 11).

### There is some TMRE fluorescence in the matrix

According to the model, the free concentration of TMRE (inside the matrix), though small, should be observed in both cases whether the membrane potential ΔΨ_m_ = 0 or not. This fits our observation in Figure 2F. The TMRE intensity in the matrix, in arbitrary units used, was nonzero even after CCCP collapsed the voltage.

### Postfacto Justification To Use Nernst Equation in TMRE Imaging Studies

At zero voltage, the bound TMRE is split roughly 50/50 on the inside/outside binding site. At finite voltage, the TMRE bound to the outside stays the same, but the TMRE bound to the inside is larger by a factor of e^∧^(−qΔΨ_m_ /*k*_B_*T*). For a typical membrane voltage of 150 mV, this is a factor of 400. Therefore, at finite voltage, the intensity of fluorescence is dominated by the TMRE bound to the matrix side of the membrane. In this way, the Nernst equation *can* be used as a semiquantitative measure of the local voltage of the membrane. Furthermore, it can even be used to determine the local voltage. Only when the voltage gets low compared to kT does this approximation break down. This postfacto justifies the use of the Nernst equation to image the membrane voltage in super-resolution microscopy studies of live cells and mitochondria, *and* studies where only the total uptake of TMRE is measured. This means future researchers can use the Nernst equation in super-resolution studies of mitochondrial membrane potential with this justification, but the interpretation should include rather than ignore the membrane binding, quantitatively modeled in this paper in the context of super-resolution microscopy.

## Conclusion

### High spatial resolution cristae electrophysiology during different metabolic states can be studied with isolated mitochondria using super-resolution microscopy

Based on the correct interpretation of Rottenberg’s 1984 membrane binding model,^35^ we can now quantitatively study mitochondria super-resolution electrophysiology and ultrastructure in different metabolic states. Motivated by the original observation by Hackenbrock^16,17^ that mitochondrial morphology changes in response to metabolic status (measured via TEM), we aimed to determine if the electrical voltage distribution also changed. We imaged the membrane potential (via super-resolution imaging of the TMRE fluorescence intensity) of intact, functional isolated mitochondria under different respiratory states, using pharmacological manipulation of the electron transport chain. Figure 4A shows the time-lapse super-resolution images of mitochondria in different respiratory states. In this study, we fed electrons into the ETC through complex II using succinate as the substrate to initiate state II respiration. We then added ADP to initiate state III respiration which on completion of ADP phosphorylation resulted in state IV respiration.^7^ This demonstrates real-time, live imaging of the voltage changes in mitochondria with the ultrastructure clearly resolved, as the mitochondria are put into different metabolic states.

Using this method, we demonstrate that the TMRE fluorescence intensity (indicative of local membrane potential ΔΨ_m_) is bright in both metabolic states, getting brighter in state IV (defined as the presence of substrates but without ADP hence without ATP synthesis), and getting dimmer when ADP is present (due to the synthesis of ATP consuming some of the energy stored in the membrane potential). Furthermore, we show clearly that the dark regions remain dark in both metabolic states. This clearly indicates that the primary binding site of the fluorophore is at the cristae, when it comes to electrical voltages, regardless of the metabolic state.

Figure 4B shows the average TMRE fluorescence intensity over the entire organelle (defined as a region-of-interest (ROI) as discussed in the Methods section and SI section 7) as a function of time, under different buffer chemistries designed to manipulate the ETC and, thus, the mitochondria membrane potential. The implicit assumption is that the TMRE average so determined can be used as n_i_ in the Nernst eq 1 to determine the ΔΨ_m_ average.

Consistent with the expectations from standard respiration studies,^7^ including our studies with external TPP^+^ electrodes which corroborated the findings,^25,26^ succinate addition increased TMRE fluorescence indicating the rise in mitochondrial membrane potential (Figure 4C). Addition of ADP caused a decline in TMRE fluorescence indicative of the utilization of the membrane potential by the ATP synthase to convert ADP to ATP, and when the phosphorylation of ADP was complete and the ATP synthesis stopped, the TMRE fluorescence increased due to the recovery of the membrane potential via state IV respiration. A second addition of ADP repeated the state III to state IV progression.

Additionally, to confirm prior results still apply in our measurements, we showed that oligomycin causes the TMRE fluorescence to increase (indicating that the average membrane potential increased): Inhibition of the ATP synthase with oligomycin drives the mitochondrial electrochemical gradient to its maximum resulting in maximum TMRE uptake and fluorescence. Prolonged oligomycin exposure caused the membrane potential to decline, a phenomenon commonly observed, perhaps due to the activation of the mitochondrial permeability transition pore (mtPTP). Furthermore, we showed that, with the addition of CCCP, the average TMRE fluorescence drops close to zero: Treatment with the mitochondrial uncoupling agent CCCP collapses the inner membrane electrochemical gradient resulting in the release of the mitochondrial TMRE and minimal mitochondrial fluorescence. Hence, with intact, functional mitochondria, the uptake and release of TMRE follow the expectations of changes in the mitochondrial membrane potential during OXPHOS function. This is reproducible over all 4 cell lines studied (see SI section 12). Additionally, mitochondrial heterogeneity as well as spatial and temporal fluctuations can be further analyzed (see SI section 16).

## Discussion

### Bulk models may break down in the cristae: There is only one (calculated) H^+^ ion in the cristae solution at known pH values, but electric fields are extremely intense

**Figure 5:**
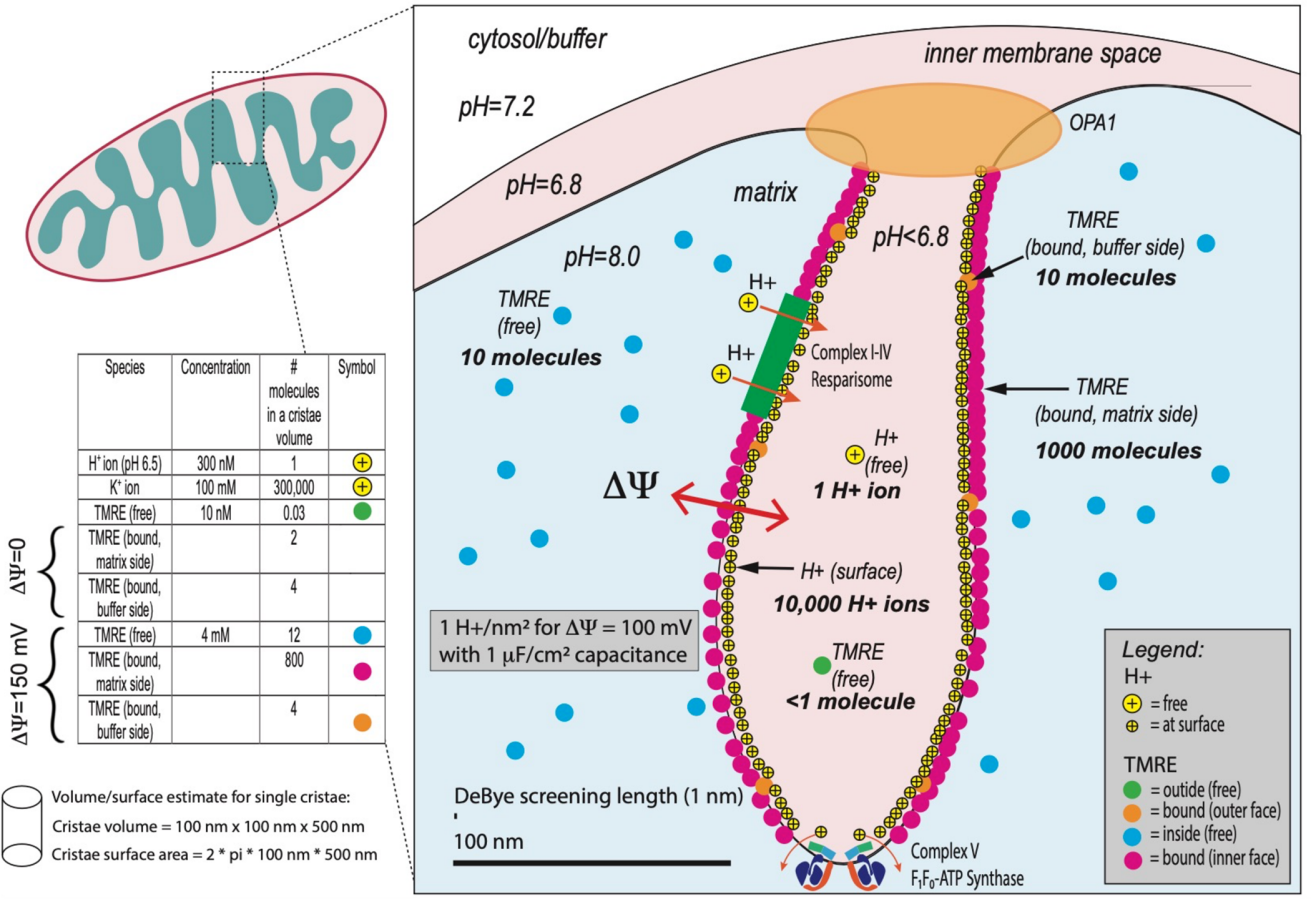
Schematic diagram of several different species and their distribution within a single cristae finger. Not shown are the OH- species on the membrane needed to maintain charge neutrality.

We now discuss the distribution of various charged species near a single cristae finger (Figure 5). Note that this is for discussion purposes only and does not detract from the main result of the paper that TMRE is localized on the matrix side of the mitochondrial inner membrane when there is a sustained membrane potential. In this discussion, we seek to put into perspective the electric fields, pH, and ionic species of the mitochondria as a whole organelle, from a systems perspective, and to indicate schematically what the TMRE fluorescence images represent physically.

We first discuss TMRE distribution. As discussed above, at the TMRE concentration used, the TMRE charge density is small compared to the other ionic species. Therefore, the TMRE does not significantly change any electric fields; rather it only acts as a probe of those fields and responds to them. Approximating a single cristae finger as a cylinder of diameter 100 nm and length 500 nm for volume and surface area estimates, and using known binding constants discussed in detail above and in the Supporting Information, based on a membrane potential of 150 mV, we find there will be ∼1,000 bound TMRE molecules at the surface on the matrix side of a single cristae finger (red dots). *These give the dominant observed fluorescent signal in super-resolution images.* The TMRE is positively charged, so there will be ∼1000 negative charges on the other side of the membrane (cytosol/buffer side), presumably OH^–^ species (not shown). The remaining TMRE is small in number and difficult to observe in super-resolution images: In the matrix, there will be ∼10 free TMRE molecules in the field of view (blue dots). Inside the finger, there will be less than one free TMRE molecule (green dot) and less than ∼10 bound TMRE molecules (orange dots) on the cytosol/buffer side of the membrane.

We turn next to the unbound hydrogen H^+^ and OH^–^ species. We assume the pH = 7.2 in the cytosol/buffer. Because protons are pumped out of the matrix, the matrix must be at a higher pH than the cytosol/buffer. While the exact matrix pH is difficult to measure, it is generally accepted to be between 7.4 and 8 (corresponding to a delta pH across the mitochondrial membrane of somewhere between 0.2 and 0.8 pH^36−41^). The intermembrane space (IMS) has a lower pH than the cytosol. Although not exactly known, these are indicated as 7.2, 6.8, and 8.0 in the cytosol (buffer), intermembrane space (IMS), and matrix, respectively, in the figure. We and others^39^ have shown that protons are concentrated in the cristae fingers since the OPA1 and MICOS complexes block proton transport through the tight cristae junctions. Since the source of the protons is dominantly inside the cristae fingers where the components of the ETC are localized,^10^ the effective pH inside the finger would be lower than the IMS, i.e. lower than 6.8. In order to estimate the # of protons inside a single finger, we estimate the volume as a cylinder of diameter 100 nm and length 500 nm. Based on this, if the pH inside the finger is 6.5, there will be statistically about one H^+^ ion in one cristae. Since the application of the concept of pH assumes a statistical distribution of a large number of OH^–^ and H^+^ ions, its application to a volume with less than one H^+^ ion indicates that a new model is needed.

We now turn to bound hydrogen H^+^ and OH^–^ species. Since there is a membrane potential of around 150 mV across the membrane, by Gauss’s law of electrostatics, there must be a sheet of positive charge on the inside of the cytosol/buffer side of the membrane (pink area), and a corresponding sheet of negative charge on the matrix side of the membrane (blue area). Based on an estimated bilayer capacitance per unit area of 1 μF/cm^2^, the amount of charge can be estimated as one electron equivalent of charge per nm^2^, positive on the cytosol/buffer side of the membrane, negative on the matrix side of the membrane. We postulate that the molecular identity of positive charges on the cytosol/buffer side are H^+^ ions, since these are the ions that are actively and continuously being pumped by the ETC to maintain the membrane potential. Approximating the total surface area of a single cristae finger’s membrane as a cylinder of diameter 100 nm and length 500 nm, the number of estimated charges per cristae finder is 10,000 (shown as yellow circles with a plus sign). Similarly, there would be 10,000 OH^–^ charges on the matrix side of the membrane (not shown).

The electric fields inside the cristae are intense. This huge imbalance between one free H^+^ ion and 10^5^ on the surface *must* affect the electrostatics inside the cristae to create immense electric fields. This would also have a huge potential to impact the protein complexes in the ETC. Clearly, a new model, not based on bulk, continuum distributions of charge and pH, which takes into account the immense electric fields and their impact on the ETC performance under different metabolic conditions is needed. The recognition of the few # of protons in a mitochondrion was also recently pointed out by Silverstein.^42^ Development of theories and experiments in this “stochastic” limit has begun in test systems.^43,44^ It has been shown that the classical definition of pH in very small volumes needs to be revised when applied to fluorescent probes of pH in mitochondria.^45^ Our super-resolution studies represent a significant step in experimentally dissecting the super-resolution electrophysiology of this organelle.

It is not unreasonable to speculate that, even given the Debye screening length of a few nanometers, at least in some regions of the cristae, the effective pH is not 6 or 7 but much more acidic,^46,47^ even approaching pH 1. One might even speculate even further that these strong fields affect the spin polarization and may even give rise to quantum effects in the microscopic environmental chemistry or even the macroscopic phenotype. Hints of this have already been reported in the literature.^48^ While in their infancy, super-resolution studies such as these, combined with quantum probes of mitochondrial function,^49^ may enable powerful methods of probing the connection between energy and life.

## Materials and Methods

### Cell Culture and Fluorescent Dye Staining

The MB231 cells and HeLa cells used in this research were purchased from ATCC. The HEK293 cells and HK2 cells were gifts from a collaborator. All the cells were cultured for 2–3 days in 75 cm^2^ tissue flasks at 37 °C and 5% CO_2_ before being ready for experimentation. Dyes (10 nM TMRE or 100 nM MitoTracker DeepRed (MTDR) and 100 nM MitoTracker Green (MTG) or 100 nM 10-N-nonyl acridine orange (NAO) or 3 μL/ml PicoGreen; Santa Cruz Biotechnology) were added to the cell culture media and incubated 1 h prior the cell retrieval.

### Mitochondrial Isolation

After retrieval, the cells were transferred into a falcon tube with 1 mL of ice-cold RBS buffer (5 mM KCl, 1 mM MgCl_2_, 20 mM HEPES (pH 7.0)) added, and the tube was then incubated on ice for 10 min. After incubation, the solution was transferred into a glass homogenizer, and we performed 20 strong strokes separately using a loose and tight stroker. After the douncing procedure, 1 mL of homogenization buffer (450 mM Mannitol, 150 mM Sucrose, 1 mM EGTA, 40 mM HEPES, 1% (w/v) fatty-acid free BSA, 2% protease inhibitor) was added to the solution. Next, we centrifuged the homogenate at 1000*g* for 5 min at 4 °C to remove large-scale debris. The supernatant was recentrifuged at 12000*g* for 20 min at 4 °C to get purified isolated mitochondria. The pellet was collected and resuspended in 37 °C KCl buffer (140 mM KCl, 2 mM MgCl_2_, 10 mM NaCl, 0.5 mM EGTA, 0.5 mM KH_2_PO_4_, 2 mM HEPES) (pH 7.2).

### Live Isolated Mitochondria Imaging

The collected isolated mitochondria were resuspended in 37 °C KCl buffer and plated in CELLview 4-compartment glass-bottom tissue culture dishes (Greiner Bio-One, 627870), PS, 35/10 mm. A 1500*g*, 8 min centrifugation step was applied prior to isolated mitochondria imaging to spin down the isolated mitochondria to the bottom of the dish. To improve the attachment of isolated mitochondria, the dishes were coated with Poly-l-lysine (0.1 mg/mL) for 10 min. Later, we removed the solvent and put the cartridge in a fume hood for 20 min to dry up before use. Prior to image analysis, raw .czi files were automatically processed into deconvoluted Airyscan images using the Zen software.

### Protocols for Visualization of Mitochondria Structure Using Airyscan and STED Microscopy

For Airyscan: We used a Zeiss LSM900 (w/incubation chamber, set to 37 °C) with Airyscan with an alpha Plan-Apochromat 63×/1.4 Oil DIC M27 objective. We adjusted the laser power between approximately 0.3% to 2%, and the master gain between 750 and 900. We started continuous scanning at maximum speed at a zoom factor of 1, in order to obtain a relatively strong signal-to-noise ratio for imaging. Later, we brought the field of mitochondria of interest into view and stopped scanning. We selected a mitochondrion of interest and used the crop function to zoom in approximately 6.0× to 10.0× until getting clear images of cristae structures. The pixel dwell time was set between 0.85 and 1.04 μs, respectively to avoid overtime exposure of mitochondria to the laser. Note that to get clear enough isolated mitochondria images, we set the frame time to no longer than 400 ms to avoid blurring due to the movement of mitochondria. NAO and MTG were used for structural imaging (labeling the lipid bilayer), and TMRE was used for voltage imaging, as explained in the main text.

For STED: We used an Abberior Stedycon (w/incubation chamber, set to 37 °C), gracefully on loan for a demonstration from Abberior. After obtaining a relatively strong signal-to-noise ratio at a zoom factor of 1, we brought a single mitochondrion into view and stopped scanning. We used the crop function to zoom in on the image to the desired size. We used the autoadjust function to find the best pixel size and pixel dwell time for the observation. The best pixel size and pixel dwell time in our experiment were 25 nm and 10 μs, respectively. MTDR was used for structural imaging (labeling the lipid bilayer), and TMRE was used for voltage imaging, as explained in the main text.

### Respiration State Experiment

Isolated mitochondria from HeLa cells were incubated in warm KCL buffer (140 mM KCl, 2 mM MgCl_2_, 10 mM NaCl, 0.5 mM EGTA, 0.5 mM KH_2_PO_4_, 2 mM HEPES) with a temperature control setting to 37 °C once isolated from HeLa cells (State 1). The concentration of isolated mitochondria was measured by using the Bradford assay. The time-step for each image was 5 s to avoid photobleaching. The 2.5 mM Succinate was used as the substrate to trigger electron transfer (State 2) and 250 μM ADP was added later to initiate the ADP-stimulated respiration (State 3).

### Image Analysis

Processed Airyscan images were analyzed using ImageJ (Fiji) software. For all images performed in this draft, we only adjust the brightness and contrast to demonstrate relevant changes in structure. Images acquired with STED microscopy were deconvoluted using Huygens deconvolution software. Additional image analysis is described in detail in SI section 7.

## Supporting Information Available

Respiration run images (ZIP)
Respiration run videos (ZIP)
Section 1: Phototoxicity/photobleaching. Section 2: Tubular vs Circular mitochondria. Section 3: How the binding constants were measured in the literature. Section 4: Airyscan and STED give comparable resolution for TMRE voltage stains. Section 5: Isolated mitochondria from different cell lines. Section 6: Confirmation that the mitochondria isolated are of optimal function and well coupled. Section 7: Image analysis and conversion to the average fluorescence intensity of TMRE over mitochondria. Section 8: State 3/State 4 average intensity plots. Section 9: The standard deviation of the TMRE fluorescence intensity. Section 10: 3D projection. Section 11: Distribution of the dye after the collapse of the membrane potential (other cell lines). Section 12: Response to oligomycin and CCCP is reproducible among cell lines. Section 13: Experimental conditions: Yield and statistics. Section 14: Relationship to Murphy et al. Section 15: Biological Implications: Disease and health in mitochondria. Section 16: Spatial and temporal statistical properties and mitochondrial heterogeneity. Section 17: Resolution discussion. Section 18: Comparison between Airyscan, STED, and Lattice SIM microscopy. Section 19: Wide view of isolated mitochondria: “Zoom out” vs “Zoom in”. Section 20: Literature review for super-resolution live-cell imaging of mitochondria. Section 21: Chemical structure of fluorescent dyes used in this manuscript. Section 22: Confusion on the binding abounds in the literature. Section 23: Alternative methods to determine concentrations of dye inside and outside the mitochondria. Section 24: Relationship to Miller et al. (PDF)
