# Supplementary figures and images for: Super-Resolution Imaging of Voltages in the Interior of Individual, Vital Mitochondria
Source: ACS Nano. 2023 Jun 8;18(2):1345–56. doi: 10.1021/acsnano.3c02768 (PMC10795477; doi:10.1021/acsnano.3c02768)

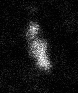

Supplement: Supplementary file 1 — nn3c02768_si_001.zip [file nn3c02768_si_001.zip › Run_respiration_exp1_mito1.tif]

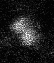

Supplement: Supplementary file 1 — nn3c02768_si_001.zip [file nn3c02768_si_001.zip › Run_respiration_exp1_mito2.tif]

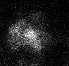

Supplement: Supplementary file 1 — nn3c02768_si_001.zip [file nn3c02768_si_001.zip › Run_respiration_exp1_mito3.tif]

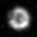

Supplement: Supplementary file 1 — nn3c02768_si_001.zip [file nn3c02768_si_001.zip › Run_respiration_exp2_mito1.tif]

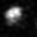

Supplement: Supplementary file 1 — nn3c02768_si_001.zip [file nn3c02768_si_001.zip › Run_respiration_exp2_mito2.tif]

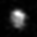

Supplement: Supplementary file 1 — nn3c02768_si_001.zip [file nn3c02768_si_001.zip › Run_respiration_exp2_mito3.tif]

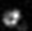

Supplement: Supplementary file 1 — nn3c02768_si_001.zip [file nn3c02768_si_001.zip › Run_respiration_exp3_mito1 .tif]

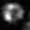

Supplement: Supplementary file 1 — nn3c02768_si_001.zip [file nn3c02768_si_001.zip › Run_respiration_exp3_mito2.tif]

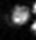

Supplement: Supplementary file 1 — nn3c02768_si_001.zip [file nn3c02768_si_001.zip › Run_respiration_exp3_mito3.tif]
