# Supplementary material for: Super-Resolution Imaging of Voltages in the Interior of Individual, Vital Mitochondria
Source: ACS Nano. 2023 Jun 8;18(2):1345–56. doi: 10.1021/acsnano.3c02768 (PMC10795477; doi:10.1021/acsnano.3c02768)
Supplement: Supplementary file 3 — nn3c02768_si_003.pdf [file nn3c02768_si_003.pdf]

Supporting Information

Super-resolution imaging of voltages in the interior of individual, vital mitochondria

*ChiaHung Lee‡, Douglas C. Wallace[Δ], Peter J. Burke[†]**

[†]Department of Electrical Engineering and Computer Science, ‡Department of Biomedical Engineering, University of California, Irvine, California 92697, United States

[Δ]Center for Mitochondrial and Epigenomic Medicine, Children's Hospital of Philadelphia and Department of Pediatrics, Division of Human Genetics, University of Pennsylvania, Philadelphia, PA 19104, United States

*Corresponding Author: pburke@uci.edu

Section 1: Phototoxicity/photobleaching

Both phototoxicity and photobleaching were observed in experiments for this work. A detailed and comprehensive study of these effects is beyond the scope of this paper and will be presented in a separate manuscript (Lee, et al, manuscript in preparation). For this work, only data where phototoxicity and photobleaching were not observed are presented.

Section 2: Tubular vs. Circular mitochondria

In cells mitochondria are generally tubular, they typically end up round when purified. The reasons for this are discussed in another manuscript (Lee, et al, manuscript in preparation), which is beyond the scope of this paper. Briefly, the mechanical lysis causes damage resulting in spherical mitochondria, but we have developed a gentler technique in which a fraction of the isolated mitochondria are tubular. For a statistical analysis of the fraction of tubular vs. spherical mitochondria, the reader is referred to Tables 1,2 and Section 13 in supporting information.

The tubular mitochondria are believed to be most representative of the mitochondria in cells, and so are the ones studied in this paper. The data on the circular mitochondria is beyond the scope of this paper, and will be presented in another manuscript (Lee, et al, manuscript in preparation).

Section 3: How the binding constants were measured in the literature

The electron spin resonance spectra of certain spin labeled lipophilic cations depend on whether the cation was bound or free. This was used to measure the amount of bound vs. free cation in liposome suspensions in 1978 [1], for zero voltage and the controlled voltage across the liposome. This proved that the amount of bound cation was voltage-dependent. Furthermore, measurements showed that the rate/binding constants were such that the amount of bound cation (by mole #) was much larger than that free and inside the liposome, by at least an order of magnitude.

The rate constants and binding were quantitatively measured in mitochondrial suspensions in 1987 by Kamo [2] for tetraphenylphosphonium (TPP^+) lipophilic cations: By using electrochemical detection of the TPP^+ concentration in the buffer under various conditions, the authors were able to show that, similar to the liposomes, 1) the amount of TPP^+ cation bound to the mitochondrial membrane was voltage-dependent, and 2) the number of TPP^+ molecules bound to the mitochondrial membrane was much larger than the # free inside the mitochondria (matrix). A similar series of experiments used TMRE (as we do) and optical detection of the amount in the buffer to find the quantitative rate constants for mitochondria [3]. They found that, at 28 °C, the ratio of # TMRE molecules bound to the inner membrane to the # of TMRE molecules free in the inner matrix was 60, and 129 for the similar ratio for the outer membrane binding (buffer side). (See units discussion in supporting information.)

A detailed description of the binding model

The TMRE is assumed to be low enough density (at 10 nM) so that:

- The # of TMRE per surface area of the bilayer is not saturating all of the possible binding sites. (Large-density TMRE will saturate the binding, as shown in [2,4].)
- The TMRE charge does not significantly perturb the electrostatics (electric fields), since other charged species such as H^+, K^+, Cl^-, and OH^-, are many orders of magnitude higher in concentration (μM to 0.1 M), and the fields generated by those charges is not changed much by the presence/absence of TMRE at 10 nM. That said, there will be some small OH^- or other negative charges to balance the TMRE positive charge.

The fundamental thermodynamic model regarding the binding of TMRE or any other lipophilic cation is based on the following assumption: The TMRE can move between different compartments, and the compartments are in equilibrium. The model used in this paper has four compartments, shown in Fig. 3.

Here, equilibrium means there is no net change in the average TMRE concentration with time, although it may fluctuate about the mean. Thermodynamics considerations of the Gibbs Free energy [5] imply that the electrochemical potential is constant. (At zero membrane voltage, the electrochemical potential is the chemical potential). If the energy of each compartment was the same, this would mean that the density of TMRE is the same in each compartment. However, there is a potential well for bound TMRE (see Fig. 3 in the main text), indicating that the densities will not be the same, even though the chemical potentials are the same.

The "density" of TMRE in the bilayer membrane is defined as the # of TMRE molecules/volume of the membrane compartment. The volume of the membrane compartment is equal to the membrane surface area times the width of the compartment (indicated as δ in Fig. 3). This is a model, which will be compared to experiments presently (below).

We make the following definitions for the density of TMRE molecules: n_i is the free TMRE inside a liposome or in the case of mitochondria inside the mitochondria, i.e. in the region called the matrix. n_{bi} is the density of bound TMRE bound to the inside surface of the membrane (in the case of a liposome) or the inside i.e. matrix surface of the membrane (in the case of mitochondria). Similarly, n_o is the density of free TMRE on the outside of the liposome or (in the case of mitochondria) on the cytosol (in cells) or buffer (in isolated suspensions of mitochondria). n_{bo} is the density of TMRE bound to the outer membrane surface (in the case of liposomes) or the outer mitochondria membrane surface (cytosol or buffer side) in the case of mitochondria. The bilayer is not assumed to be symmetric.

We define a_o as the constant of proportionality between n_o and n_{bo}. Through the following equation:

$$n_{bo} = a_o n_o$$

Similarly, for a_i:

$$n_{bi} = a_i n_i$$

Our definition does not provide any sort of a microscopic model for the origins of a_o or a_i, other than the assumptions of thermodynamic equilibrium and the overall shape of the potential shown in Fig. 3. The bilayer is not assumed to be symmetric, so we do not assume a_o and a_i are equal. Our definition is for expositional clarity in this paper's context, and not a standard in the literature. We do assume a_o and a_i are constants regardless of the membrane potential. This assumption is implicit in all of the literature to date [1–3].

Various authors in the literature have provided microscopic models for the value of a_o. Rottenberg [4], instead of a_o, used $K_{mo}\alpha_o$, where "K_{mo} is the ion partition coefficient for the external surface of the membrane and α_o is a function of the external surface potential ψ_o". Note that the surface potential is NOT the membrane potential, and is assumed to be constant [6].

The units of a_o or a_i are dimensionless. However, in practice, one does not easily know, and cannot easily measure, the exact surface area of the lipid bilayer of a mitochondrion. Furthermore, one cannot easily know, and cannot easily measure, the width of the binding potential well (δ in Fig. 3). Therefore, one cannot easily know or measure the "density" n_{bi} or n_{bo} of TMRE in the membrane compartment.

Binding constant per mg of mitochondrial protein:

Case I: $\Delta\Psi_m = 0$:

The experimental quantity measured with regard to mitochondrial suspensions is the total amount of protein in mg. This is usually measured by the Bradford assay, which measures optical absorption at a specific wavelength and converts this to mg of mitochondrial protein using an agreed upon constant from the literature [7].

The inner volume of the mitochondria (matrix volume) is usually taken as 1-2 μl/(mg of mitochondrial protein). From this, the total # of TMRE molecules can be estimated in the matrix, given the quantity of mitochondrial protein measured via the Bradford assay, and the density of TMRE (in moles/μl i.e. molarity). At $\Delta\Psi_m = 0$, the molarity of TMRE is the same on the inside (matrix) and outside (buffer), which is easily measured.

For a given amount of mitochondrial protein in mg, how many TMRE molecules are bound to the surface? This can be expressed as moles/(mg mitochondrial protein). For a given amount of mitochondrial protein in mg, how much "volume" of membrane binding compartment is there? (This would be in Fig. 3 the membrane surface area times δ.) This would be expressed as μl/(mg mitochondrial protein).

Matrix side:

(Considering the matrix side first): Rottenberg [4] defines "apparent internal partition coefficient" K_i' as our a_i times the membrane volume V_{mi} per mg mitochondrial protein:

$$K_i' \equiv a_i V_{mi} / (\text{mg mitochondrial protein})$$

With these units, for a concentration n_i of TMRE in the matrix, the # of bound TMRE molecules would be:

(eq. 1) # bound TMRE molecules (matrix side) = $n_{bi}\ V_{mi} = a_i\ n_i\ V_{mi} = K_i'$ (mg mitochondrial protein) n_i

From Rottenberg, 1984 [4]:

> *The membrane volume occupied by the phospholipids on the matrix and cytosolic surface of the inner membrane is approximately half the volume of the total phospholipids of the inner membrane. Quite likely the lipophylic cations occupy only a fraction of this volume. While this volume fraction is unknown it is proportional to the total membrane volume and therefore to the membrane protein content which is routinely measured. In the applications that follow the apparent surface membrane concentration is expressed in units of nmol/mg protein. The units of the apparent partition coefficients K', (nmol/mg protein)/(nmol/ µl), are therefore expressed in µl /mg protein.*

These units are confusing, but they are what Rottenberg [4] chose to use, and also authors since then [1,2] have used this nomenclature. Similarly, the # of free TMRE molecules in the matrix can be expressed as:

free TMRE molecules (in matrix) = $n_i\ V_i$

Again, one does not know the internal volume V_i easily. However, since the inner volume of the mitochondria (matrix volume) is usually taken as 1-2 µl/(mg of mitochondrial protein), one can calculate:

(eq. 2) # free TMRE molecules (in matrix) = $n_i\ V_i$ = (mg mitochondrial protein) (2 µl/mg) n_i

On comparison with the formula for the # of bound TMRE molecules, one can see the K_i' has units of µl/mg.

Rottenberg and other authors have expressed experimentally determined binding constants as K_i'.

In comparing eq. 1, 2, once can see that if $K_i' > 1\text{-}2$ µl/mg, more TMRE is bound to the membrane than is free in the matrix, i.e. the ratio is:

bound TMRE molecules (matrix side) / # free TMRE molecules (in matrix) =
K_i' / (1-2 µl/mg).

Scaduto[3] found, for TMRE at 28 C, K_i'= 60 (Table 1, Scaduto). ***This indicates 60x more TMRE molecules are bound to the inner membrane (matrix side) than free TMRE molecules in the matrix (at*** $\Delta\Psi_m = 0$***).***

Buffer side:

A similar line of definitions applies for binding to the outer side of the membrane (the buffer side):

$n_{bo} = a_o n_o$

$K_o' \equiv a_o V_{mo}$ / (mg mitochondrial protein)

(eq. 3) # bound TMRE molecules (buffer side)
= $n_{bo} V_{mo} = a_o n_o V_{m0} = K_o'$ (mg mitochondrial protein) n_o

In comparing eq. 1, 3, once can see that if $K_o' > 1\text{-}2$ μl/mg, more TMRE is bound to the membrane than is free in the matrix, i.e. the ratio is:

bound TMRE molecules (buffer side) / # free TMRE molecules (in matrix) =
K_o' / (1-2 μl/mg).

Scaduto[3] found, for TMRE at 28 C, K_i'= 129 (Table 1, Scaduto). ***This indicates 129x more TMRE molecules are bound to the outer membrane (buffer side) than free TMRE molecules in the matrix (at $\Delta\Psi_m = 0$).***

Case II: $\Delta\Psi_m \neq 0$:

If $\Delta\Psi_m \neq 0$, the free concentration ratio is governed by the Nernst equation:

$n_i/n_o = e^{(-q\Delta\Psi_m / k_B T)}$,

From equation 3, the # bound TMRE molecules (buffer side) do not change from the $\Delta\Psi_m = 0$ value.

From equation 2, the # bound TMRE molecules (matrix side) do change from the $\Delta\Psi_m = 0$ value, and it becomes larger by the factor $e^{(-q\Delta\Psi_m / k_B T)}$.

Both cases are shown for n_o = 10 nM in Fig. 3.

Section 4: Airyscan and STED give comparable resolution for TMRE voltage stains

In order to better resolve the cristae voltages, we next turned to STED as a potential higher resolution solution. Airyscan functions by reducing the point spread function, while STED functions with a donut photobleaching. Depending on the conditions, Airyscan can have a resolution down to 90 nm [8] with Airyscan Joint Deconvolution. Our implementation had a theoretical resolution of 120 nm because we had not yet upgraded to the 90 nm option.

In principle, STED can have a much better resolution, down to 20 nm [9,10]. Note that STED is very dependent on the bleach pump intensity and dye characteristics, while Airyscan is not. For this reason, it has been believed that Airscan is gentler on mitochondria, which are particularly sensitive to phototoxicity. However, it is possible to perform STED on live cells and mitochondria, at least for short periods of time. We decided to investigate the resolution of these two approaches on our isolated mitochondria.

We have performed STED and Airyscan imaging on mitochondria isolated using the same protocol (other than the imaging technology), in order to compare the two approaches. For structural studies, the resolution of STED was significantly better than Airyscan (Fig. S 1). The Full-width-half-maximum (FWHM) analysis gave us a 140 nm resolution of Airyscan with NAO dye and an 80 nm resolution of STED with MitoTracker DeepRed (MTDR) dye. This is consistent with the two prior studies of mitochondria in cells which quoted the resolution [10–13], which found ~50 nm and 80 nm, 35-71 nm, and 45 nm, respectively. Those studies did not approach the ultimate resolution of STED of 10 nm, presumably because the dyes were not optimized and the pump intensity needed to achieve those resolutions would have killed the cells instantly. Thus, for structural imaging STED is clearly outperforming Airyscan, in terms of resolution, for imaging of intact functional mitochondria.

In our work, we are more interested in functional assays. Since the mitochondria are intact and functional, they should sustain a membrane voltage, and we aimed to find the method with the best possible resolution. To our knowledge, at this time there is not a STED optimized lipophilic cationic dye that can image voltages with the full potential of STED. Therefore, we used TMRE for voltage imaging. Fig. S 1 shows a line profile of TMRE-labeled mitochondria imaged under nominally identical conditions, optimized for the specific method, for both Airyscan and STED. We find a FWHM of 140 nm and 145 nm for Airyscan and STED, respectively, indicating that both methods provide comparable spatial resolution for voltage imaging. Hopefully, future research on new voltage dyes that are more optimized for STED can improve the spatial resolution beyond this.

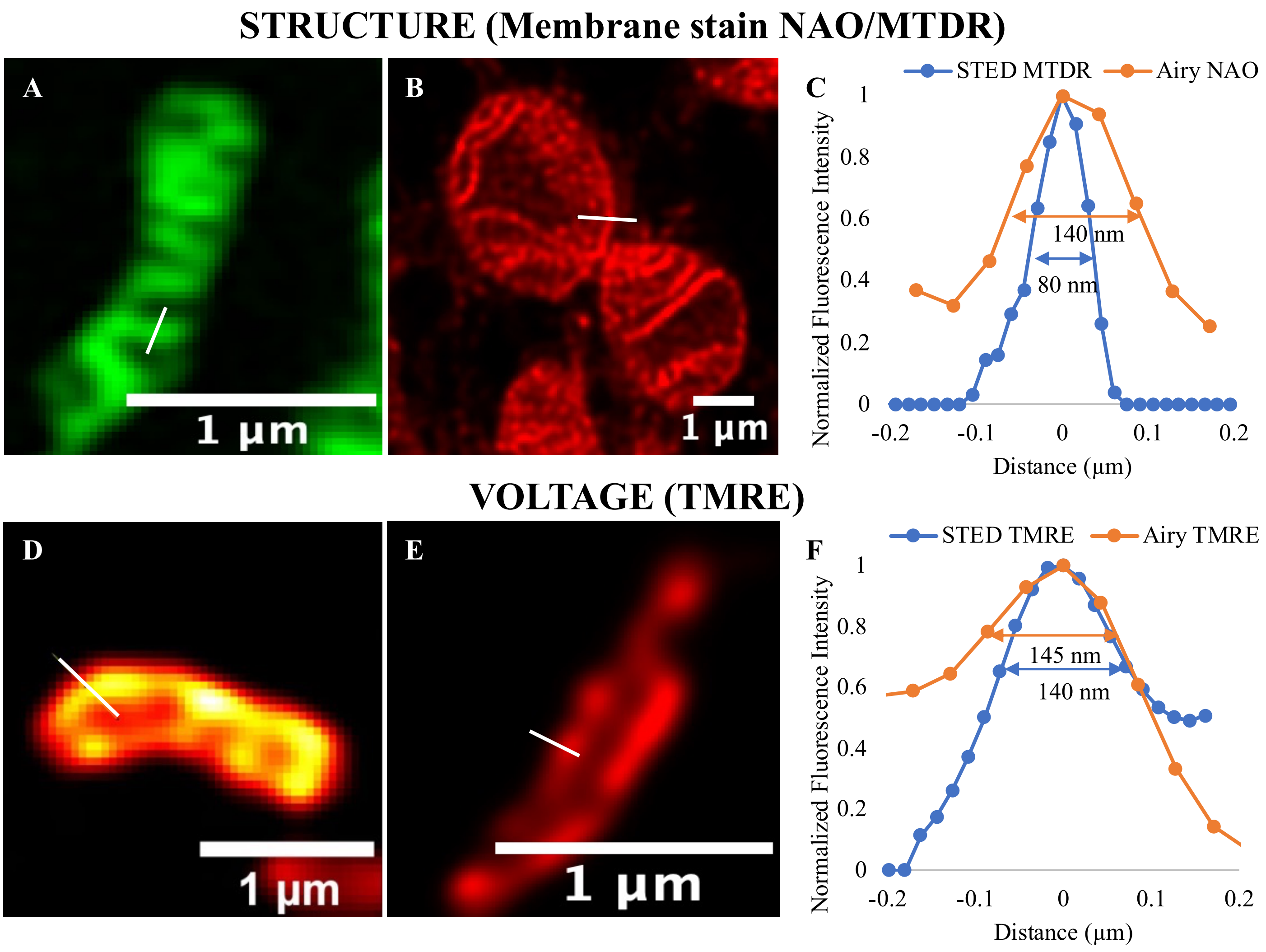

Fig. S 1. Line profile of mitochondria STRUCTURE image (A) NAO + Airyscan and (B) MTDR + STED. (C) Image resolution comparison on structure image (Normalized intensity, Full-width-half-maximum (FWHM)). Line profile of mitochondria VOLTAGE image with (D) TMRE +Airyscan and (E) TMRE + STED. (F) Image resolution comparison on voltage image (Normalized intensity, FWHM).

Section 5: Isolated mitochondria from different cell lines

We have also observed non-uniform TMRE distribution and cristae structure in 3 other cell lines (HEK293, MB231, and HK2). This indicates the phenomenon is reproducible across cell lines.

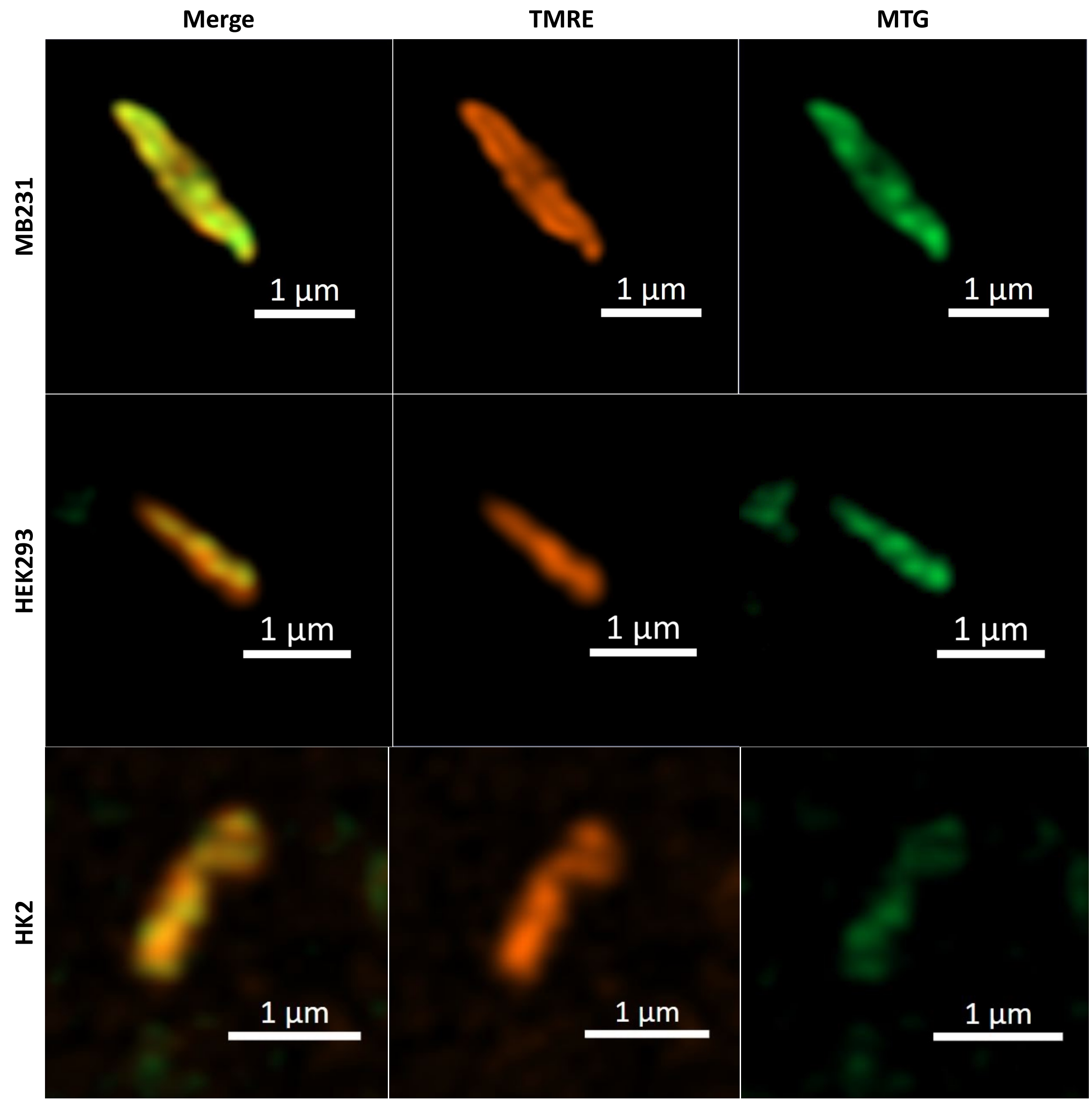

Fig. S 2. Mitochondria isolated from MB231, HEK293, and HK2 cell lines stained with Mitotracker green and TMRE.

Section 6: Confirmation that the mitochondria isolated are of optimal function and well coupled

Mitochondria are notoriously tricky to harvest from cultured cells (especially those with a low mitochondrial complement such as HeLa). Additional proof is required that what resulted was actually mitochondria and not another cell fraction. The standard method to confirm organelles are functional and well coupled is to perform respiration measurements. Therefore, to confirm that the mitochondria are of optimal function and well coupled, we performed respiration measurements matching the conditions used for microscopy (37 °C, ambient, see Method).

To quantify organelle integrity, the respiratory control ratio (RCR) is usually used [6,14]. This empirical parameter is based on the observation that mitochondria damaged during isolation show an increased proton leak of the inner membrane as compared to undamaged mitochondria, hence an increased state 4 respiration rate. The RCR is typically defined as the ratio of the state 3 to state 4 respiration rate (achieved using different substrates for the electron transport chain). The RCR assay thus measures the “leakage” of membrane potential quantitatively: Higher RCR means less damage to the mitochondria during the isolation process.

There is no ‘correct’ value for the RCR; depending on the tissue and the substrate, values can vary from 3 to 15 or more for well-prepared mitochondria [6]. We used an Agilent Seahorse XFe24 analyzer to measure the oxygen consumption rate (OCR) of mitochondria isolated using the protocol [15]. Our RCR value of isolated mitochondria from mice heart tissue is 3.9 ± 0.7 and the RCR value of isolated mitochondria from HeLa cells is 2.37, the error bars are according to the measurements of multiple wells. These data matched with historical literature consensus values to demonstrate that mitochondria are functional and are derived from proper isolation.

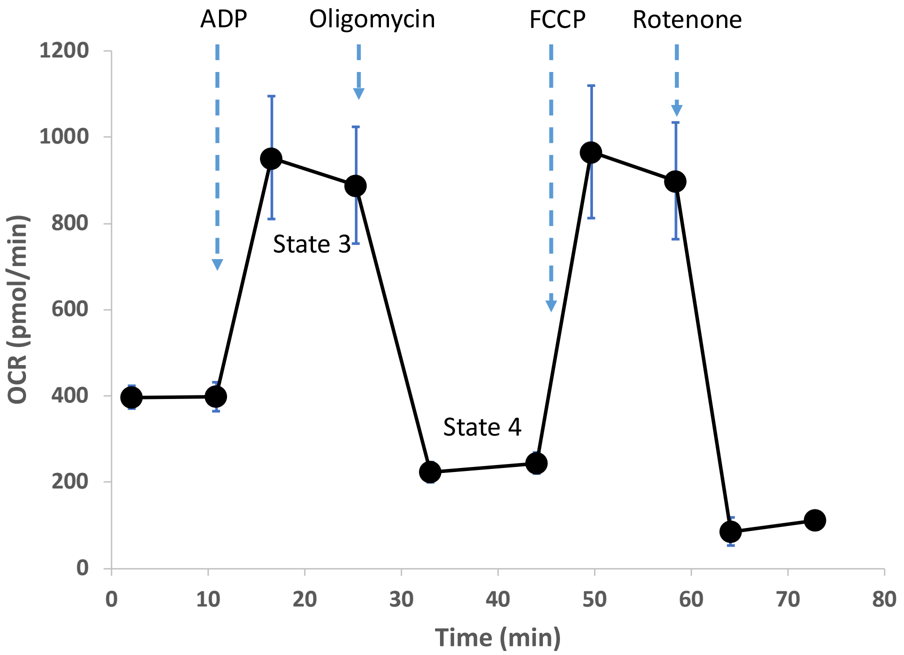

Fig. S 3. The oxygen consumption rate of isolated mitochondria from mouse heart tissue. The calculated RCR =3.9.

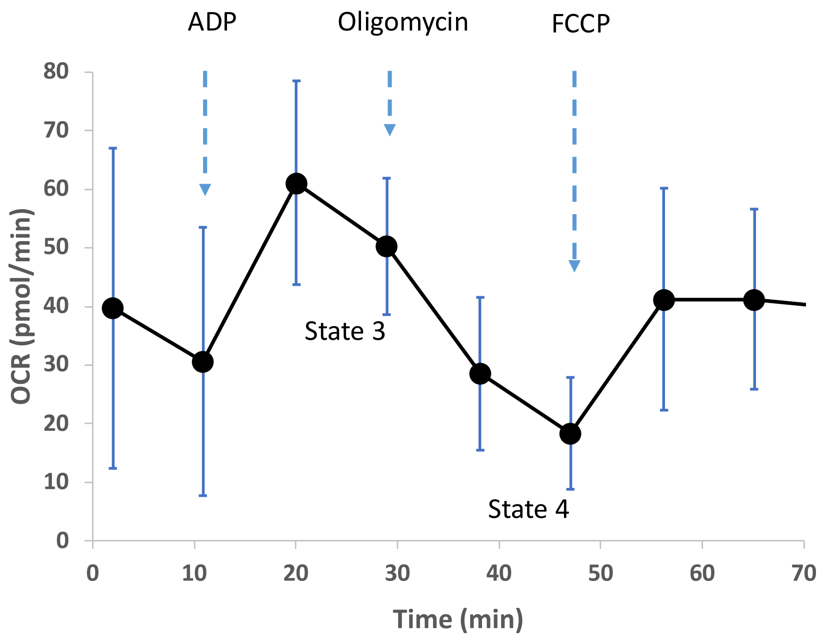

Fig. S 4. The oxygen consumption rate of isolated mitochondria from the HeLa cell line. The calculated RCR = 2.37.

Section 7: Image analysis and conversion to the average fluorescence intensity of TMRE over mitochondria

The Zeiss microscope created a time-lapse series of images (snapshots in time), typically one image per second. The exposure conditions and time-lapse rates for each experiment are listed in detail in the tables below.

These images were processed by the Zeiss software using the Airyscan algorithm on the signal from each photodetector. The resultant file was an image file (CZI format, which is a Zeiss format) which contained the processed time-lapse images, one image per frame.

Since typically more than one mitochondrion was available within the field of view, we used ImageJ to manually select a rough (rectangular) region of interest around the mitochondria and save this as a TIF file with several hundred frames. These TIF files are provided as supporting information files (*Run_respiration_exp#_mito#,* where # indicates the number of the experiment and mitochondrion) and can be viewed as movies by appropriate viewers. To be clear, these TIF files have no image processing or adjustment, they are just the original image series in Zeiss CZI cropped and converted to TIF. No information was lost or gained in this process. Thus, the TIF files are indeed the "raw data".

We wrote a script in Igor (an analysis program available for Mac or PC) to process the images. The script calculated the average intensity above a certain threshold for each frame, and then plotted that vs. time. It also calculated the std. deviation of the intensity for each frame and plotted that vs time. Pixels below the threshold were assumed to be outside of the physical region of the mitochondria. This was confirmed manually. Finally, every 6 points were averaged to give the average intensity over that period (30 seconds) and plotted. Fig. S 5 below shows the raw calculated FI and the data averaged every six points. Any reader with Igor can run this script ("macro") to reproduce the figures in this paper ("processed data") from the provided TIF movies ("raw data").

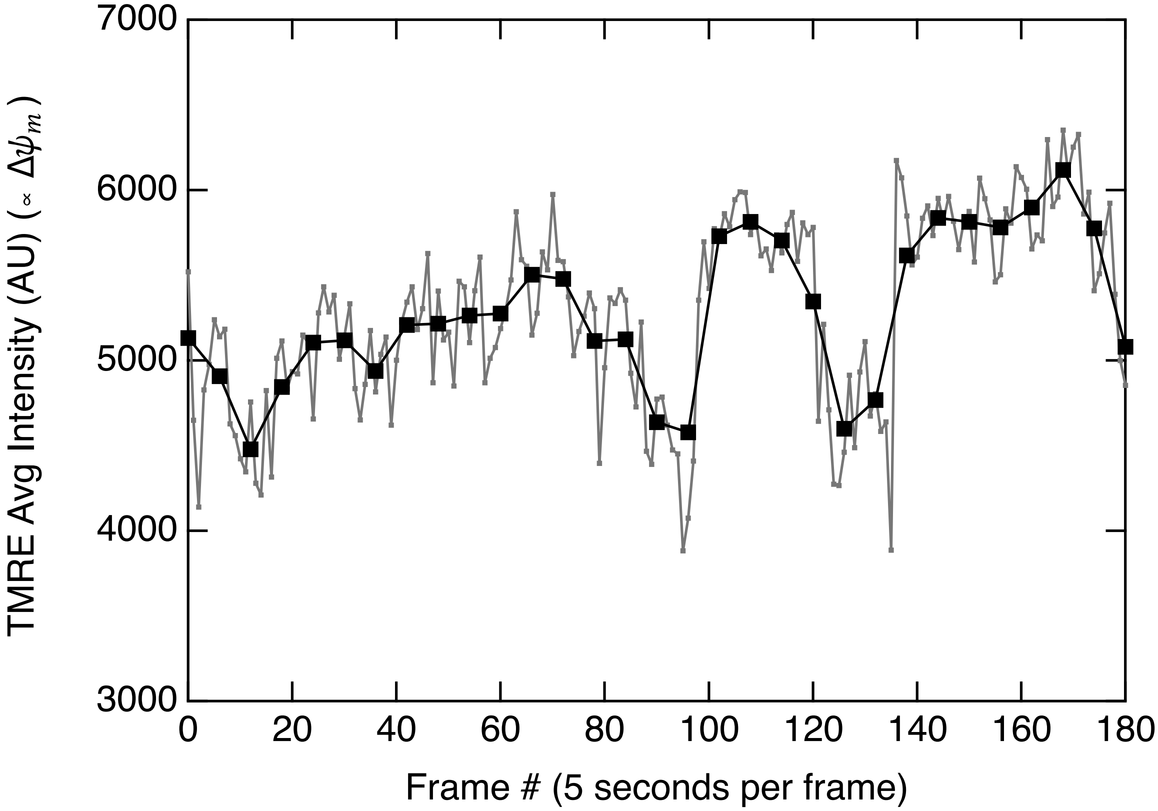

Fig. S 5. Grey points are the average fluorescence intensity of the mitochondria for each frame. Black points combine 6 frames per point to show long-term trends.

Image processing script

```
Macro loadZeissTif(imagewavename,filename,pathname, Thre)
	// This macro loads Tifs timelapse from the Zeiss
	// PJB 12/20/2022
	// Add Threshold
	// CHL 12/28/2022
	String imagewavename // Wavename of image loaded
	String fileName // name of file to load or "" to get dialog
	String Pathname // name of path or "" get dialog
	Variable Thre // Threshold, give Thre=0 not to set threshold

	Silent 1; PauseUpdate
	Preferences 1
	variable m_rows, m_columns,m_layers, m_chunks // size of each
dimension of loaded waves

	// load the image
	ImageLoad/o/T=tiff/S=0/C=-1/Q/N=$imagewavename/p=$pathname filename

	// get size of image
	m_rows=dimsize($imagewavename,0)
	m_columns=dimsize($imagewavename,1)
	m_layers=dimsize($imagewavename,2)
	m_chunks=dimsize($imagewavename,3)
	print "rows, columns, layers, chunks"
	print m_rows, m_columns, m_layers, m_chunks

	// make image waves

	imagetransform /p=3 getplane   $imagewavename
	Display;DelayUpdate
	AppendImage M_ImagePlane

	imagetransform /p=7 getplane   $imagewavename

	//without threshold
	String m_newwavename // name of 2d image wave for a particular frame
	String m_avg_wavename // name of wave with averages FI of images
	String m_stdDev_wavename // name of wave with std dev FI of images
	String m_avg_wavename_dec // name of wave with averages FI of images
	String m_stdDev_wavename_dec // name of wave with std dev FI of images

	m_avg_wavename=imagewavename+"_AVG_FI"
	m_stdDev_wavename=imagewavename+"_STD_FI"
	m_avg_wavename_dec=m_avg_wavename+"_DEC"
	m_stdDev_wavename_dec=m_stdDev_wavename+"_DEC"
	make/d/O/N=(m_layers)
$m_avg_wavename,$m_stdDev_wavename,$m_avg_wavename_dec,$m_stdDev_wavename_dec

	variable index1=0
	variable m_avg_FI,m_std_FI

	do
		// make an image
```

```
		m_newwavename = imagewavename+"frame"+ num2str(index1)
		Make/O/N=(m_rows,m_columns) $m_newwavename
		$m_newwavename = $imagewavename[p][q][index1]

		// Threshold
		// make px number < Threshold -> Nan
		setnan($m_newwavename,m_rows,m_columns, Thre)


		WaveStats/Q $m_newwavename
		m_avg_FI = V_avg
		m_std_FI = V_sdev
		print index1, m_avg_FI,m_std_FI, m_newwavename

		$m_avg_wavename[index1]=m_avg_FI
		$m_avg_wavename_dec[index1]=m_avg_FI
		$m_stdDev_wavename[index1]=m_std_FI
		$m_stdDev_wavename_dec[index1]=m_std_FI


		index1 += 1

	while(index1 < m_layers)

	Resample/DOWN=6 $m_avg_wavename_dec,$m_stdDev_wavename_dec

	display $m_avg_wavename, $m_avg_wavename_dec
	append $m_stdDev_wavename, $m_stdDev_wavename_dec
	Legend/C/N=text0/A=MC

EndMacro


function setnan(waves,row,col,Thre)
	wave waves
	variable row, col, Thre
	variable p, q
	for (p=0; p<row; p=p+1)
		for (q=0;q<col;q=q+1)
			if (waves[p][q]<Thre)
				waves[p][q]=nan
			endif
		endfor
	endfor
end function
```

Section 8: State 3/ State 4 average intensity plots

In our work, we reproduced the metabolic state manipulation experiment 3 times, and in each experiment, we studied 3 individual mitochondria that happened to be within the field of view with super-resolution microscopy. All of the traces are shown in Fig. S 6 to Fig. S 13 below.

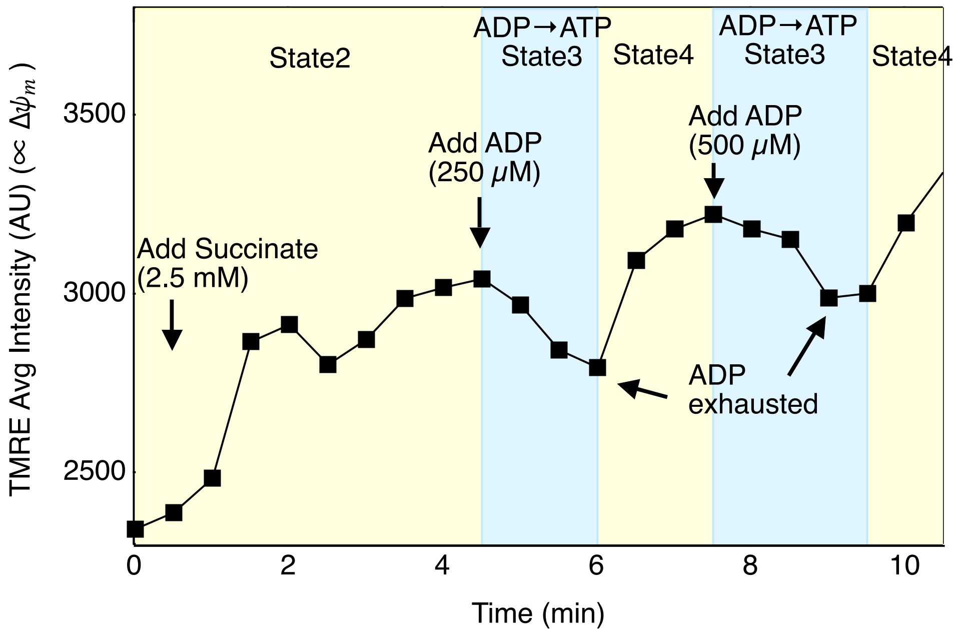

Fig. S 6. Isolated mitochondria respiration test.

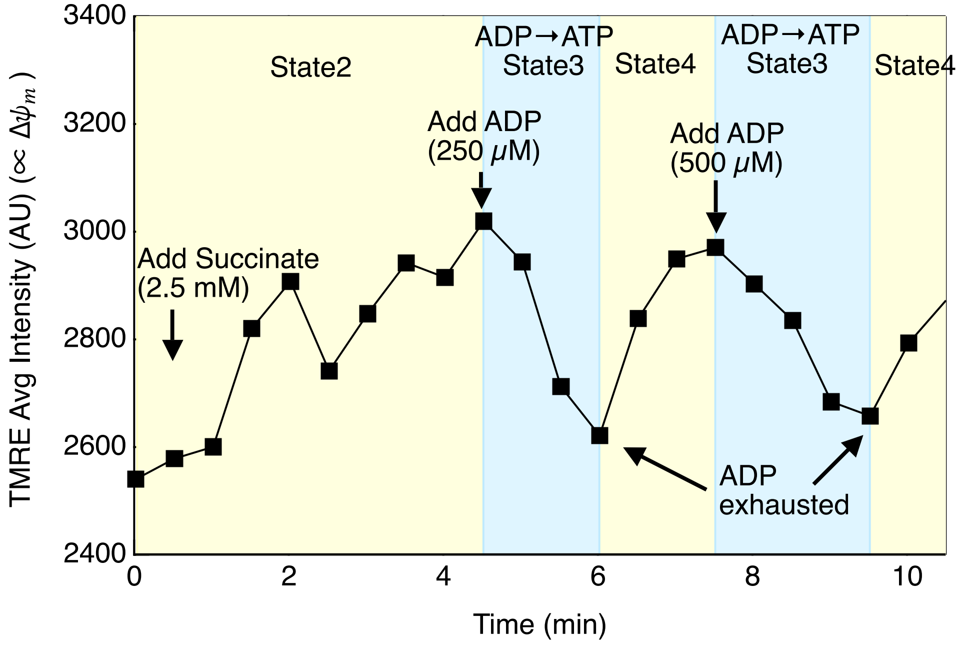

Fig. S 7. Isolated mitochondria respiration test.

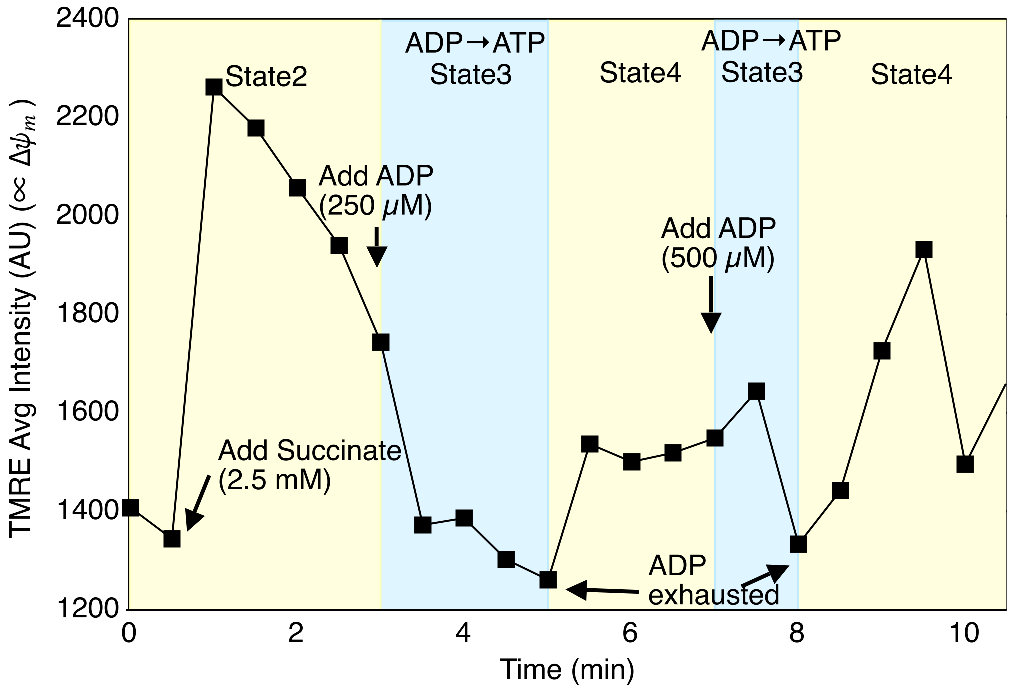

Fig. S 8. Isolated mitochondria respiration test.

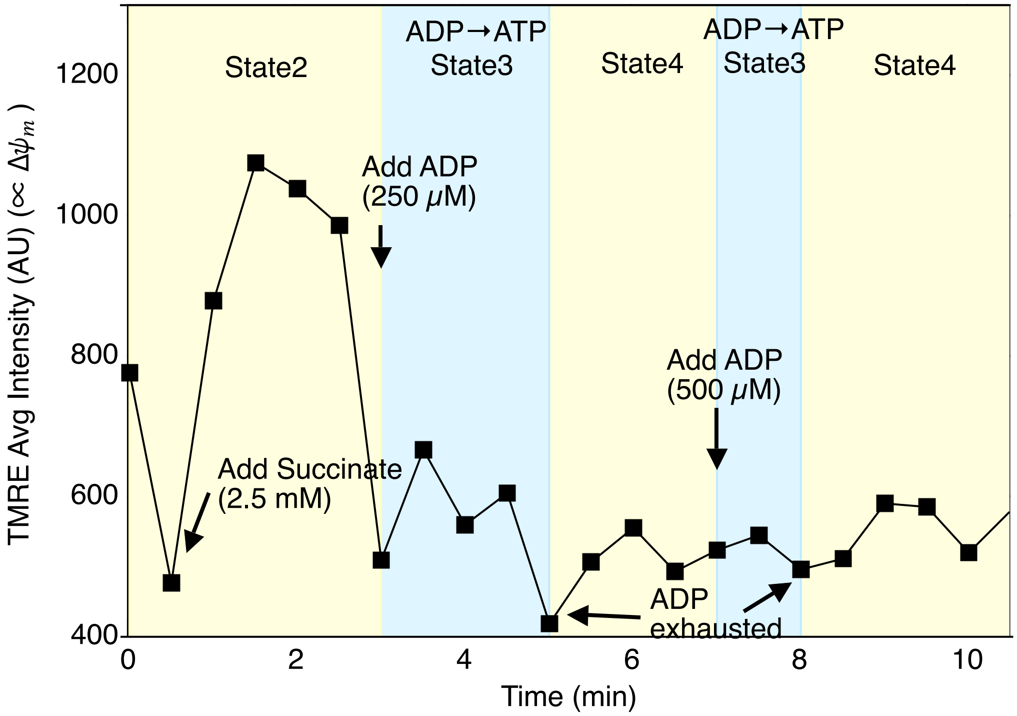

Fig. S 9. Isolated mitochondria respiration test.

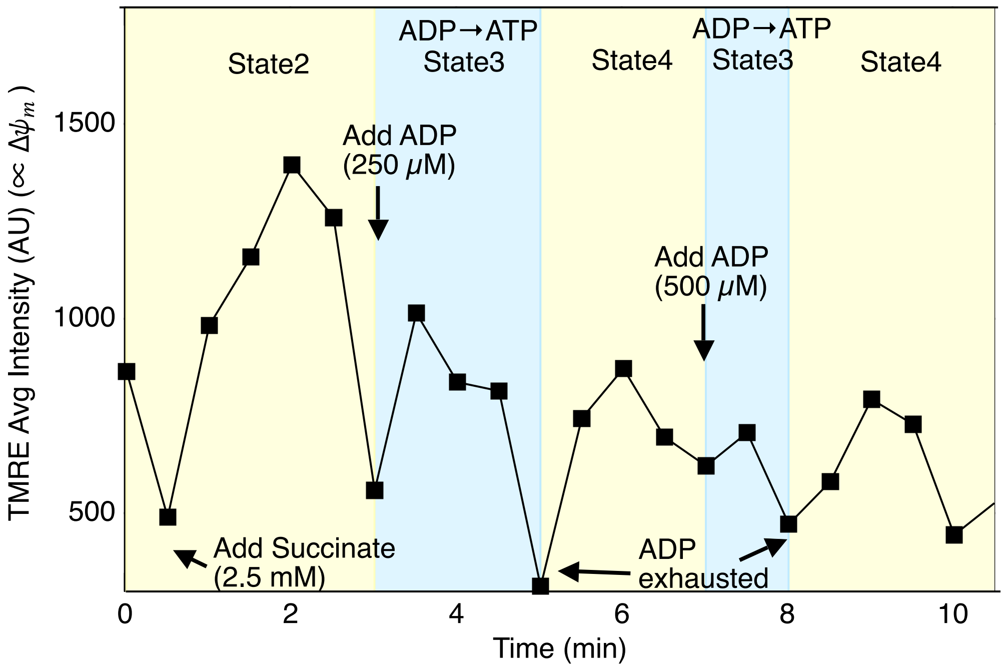

Fig. S 10. Isolated mitochondria respiration test.

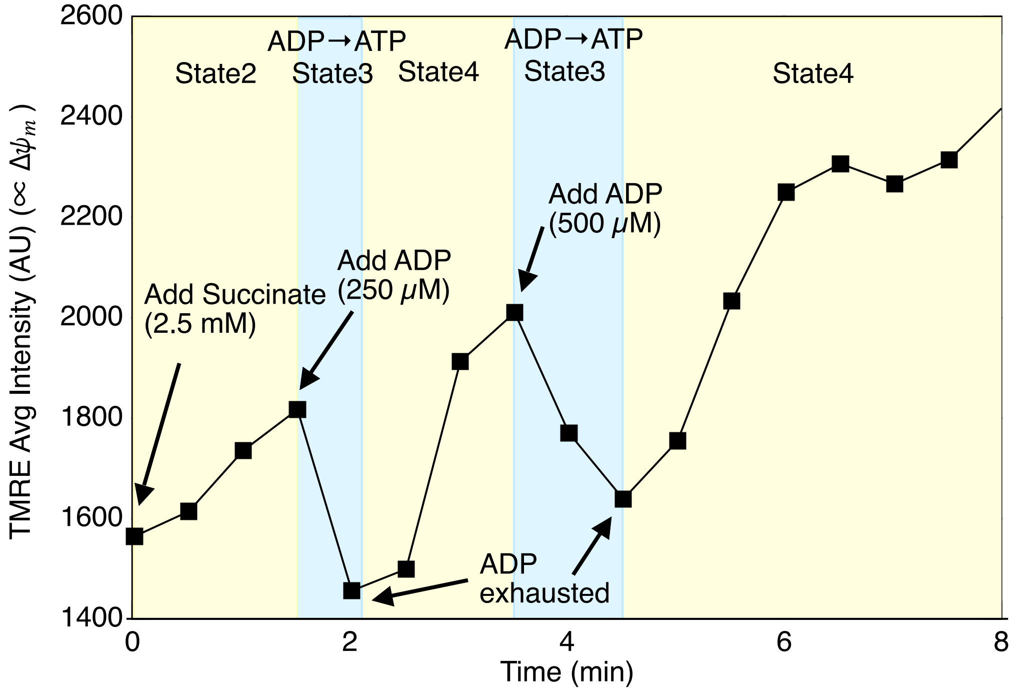

Fig. S 11. Isolated mitochondria respiration test.

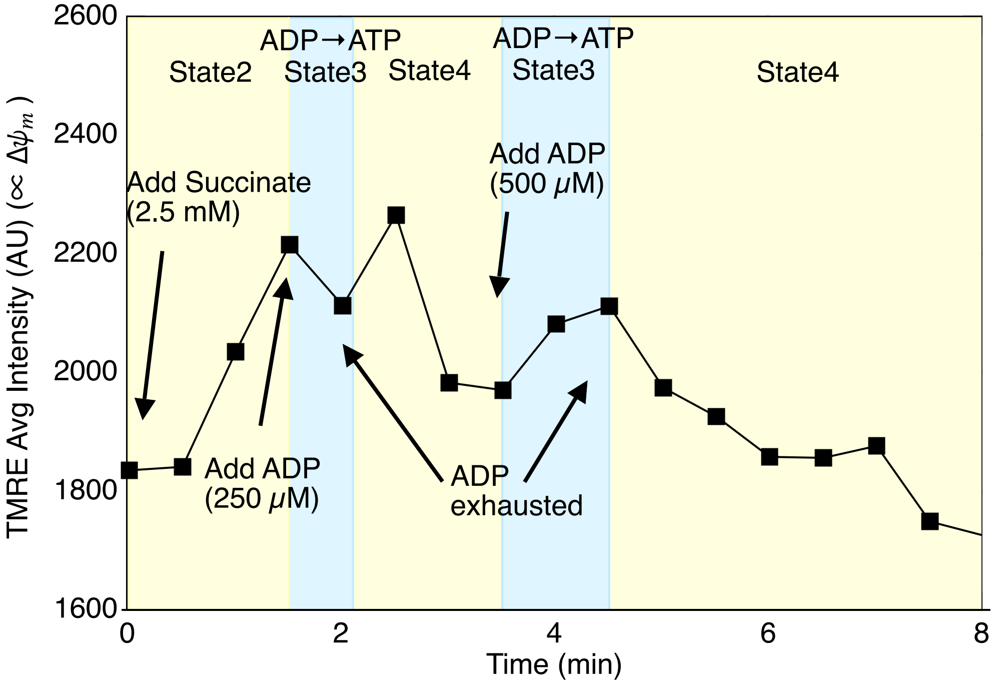

Fig. S 12. Isolated mitochondria respiration test.

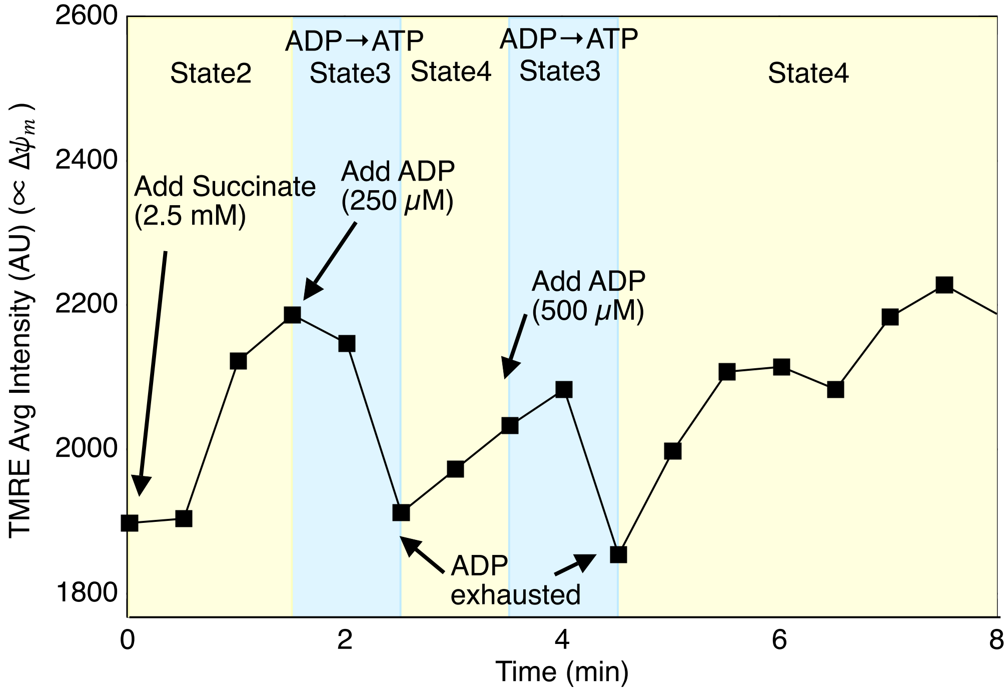

Fig. S 13. Isolated mitochondria respiration test.

Section 9: The standard deviation of the TMRE fluorescence intensity

Fig. S 14 below shows the average TMRE intensity, and the standard deviation, of the fluorescent intensity (FI) in the ROI for each frame, and the ratio of the standard deviation to the average, as a function of time, under different metabolic states. The deviation is about 60 % of the

average, indicating significant spatial fluctuations of the membrane potential due to the ultra-structure. Interestingly, this ratio does not change much with metabolic status.

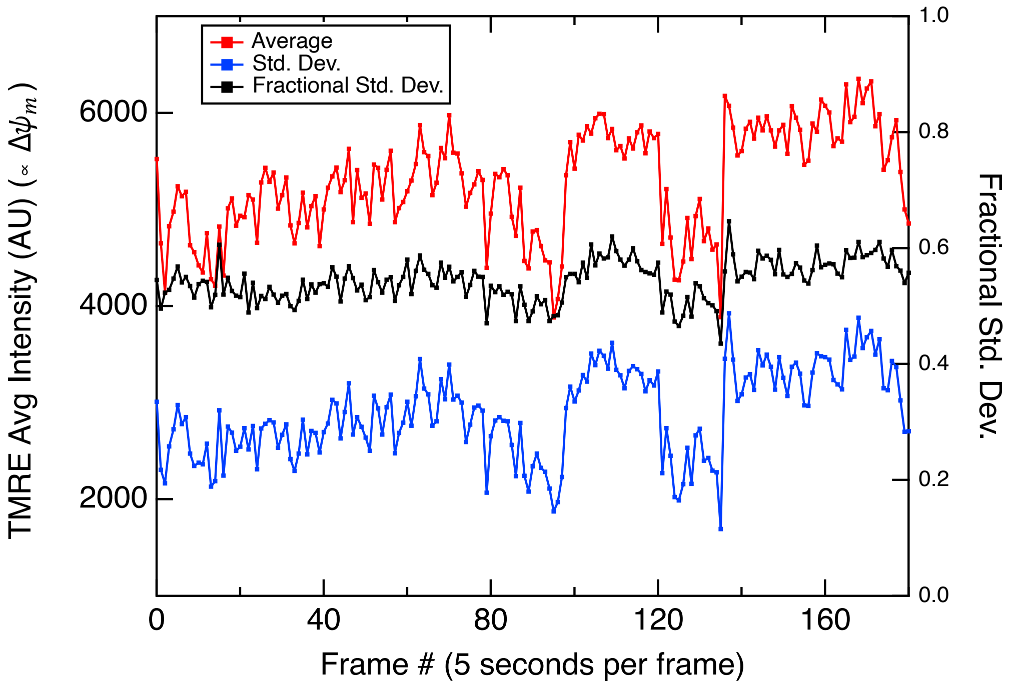

Fig. S 14. Comparison of TMRE AVG and STD intensity, and the Ratio of STD/AVG.

Section 10: 3D projection

Fig. S 15 below shows the 3D projection of one of the TMRE images showing the spatial variation in a different format.

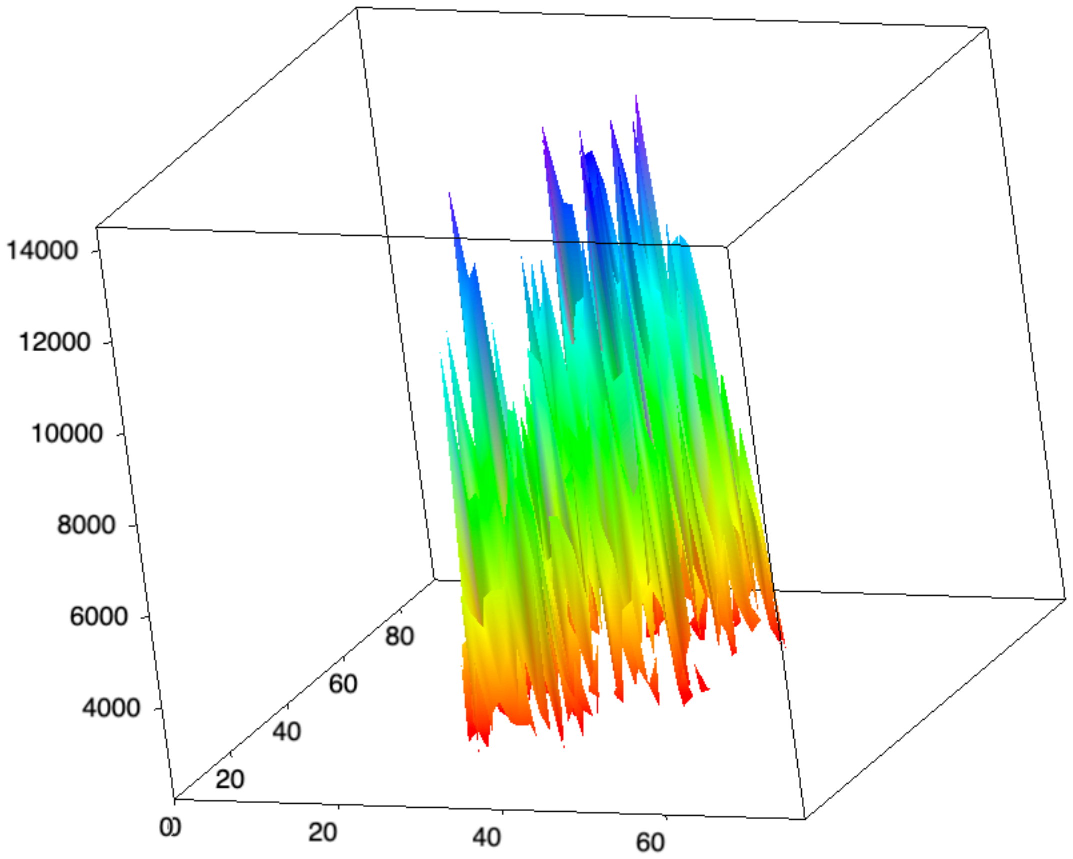

Fig. S 15. 3D representation of FI. Each pixel in XY is 48 nm.

Section 11: Distribution of the dye after the collapse of the membrane potential (other cell lines)

Fig. S 16 to Fig. S 18 below show the distribution of TMRE in isolated mitochondria before and after membrane potential collapse with CCCP. The zero membrane potential images still show structure, indicating that even under zero membrane potential, there is a small amount of TMRE bound to the membrane.

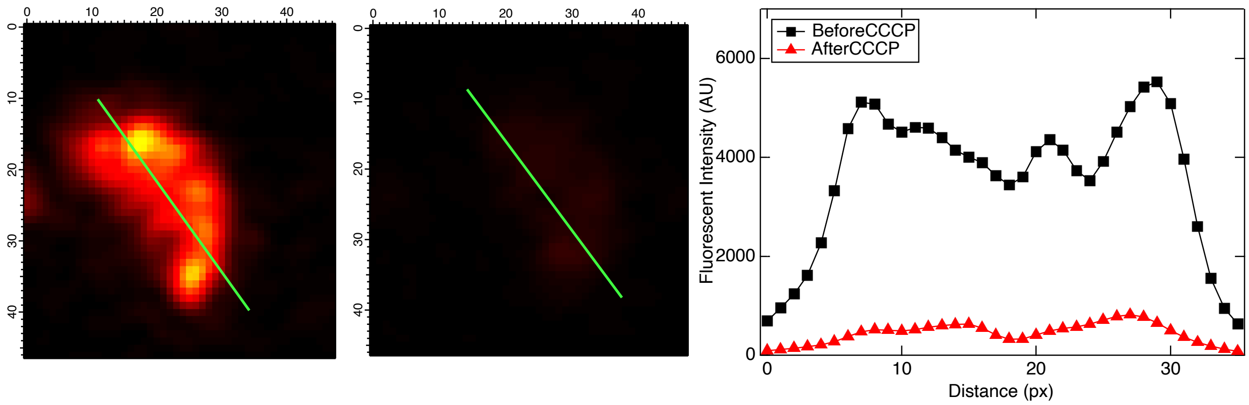

Fig. S 16. TMRE intensity of isolated mitochondria (HEK293) before and after treating with CCCP.

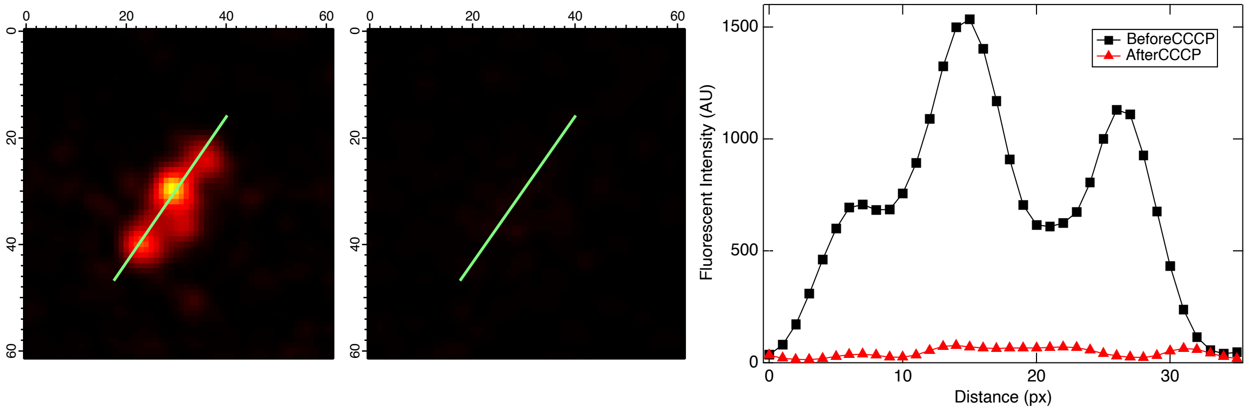

Fig. S 17. TMRE intensity of isolated mitochondria (HK2) before and after treating with CCCP.

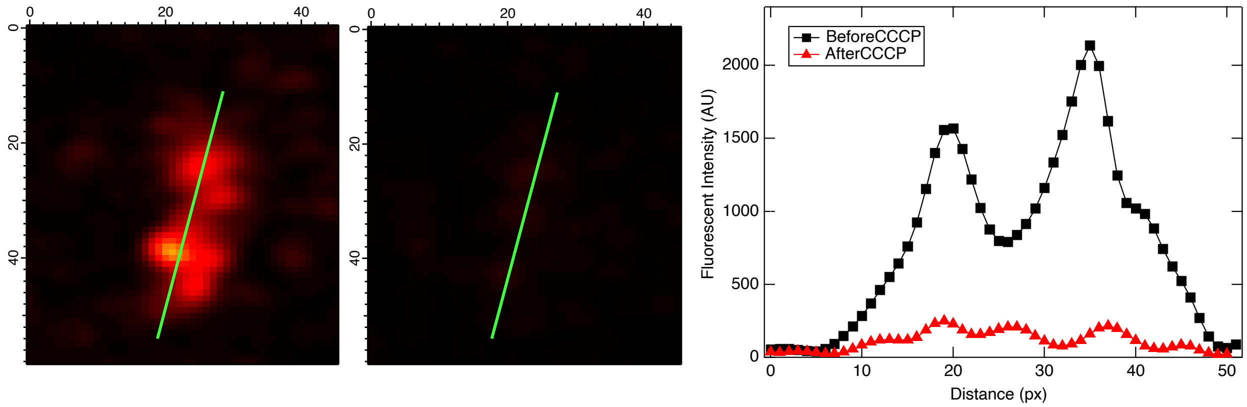

Fig. S 18. TMRE intensity of isolated mitochondria (HK2) before and after treating with CCCP.

Section 12: Response to oligomycin and CCCP is reproducible among cell lines

The response to oligomycin and CCCP presented in the main text for HeLa cells is also observed in 3 other cell lines (see Fig. S 19 to Fig. S 20 below).

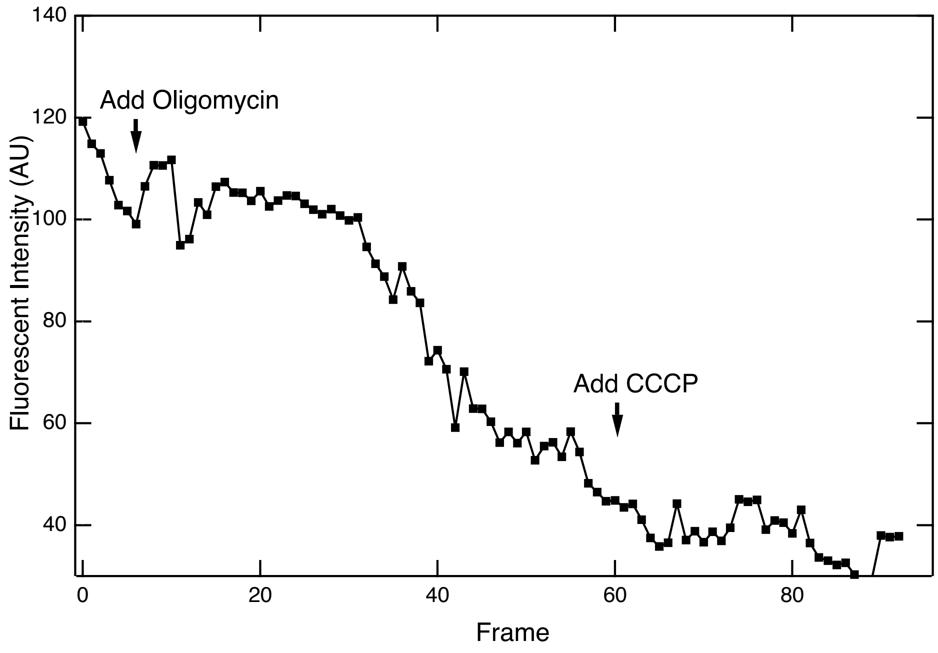

Fig. S 19. TMRE Intensity of isolated mitochondria (MB231) treated with Oligomycin and CCCP.

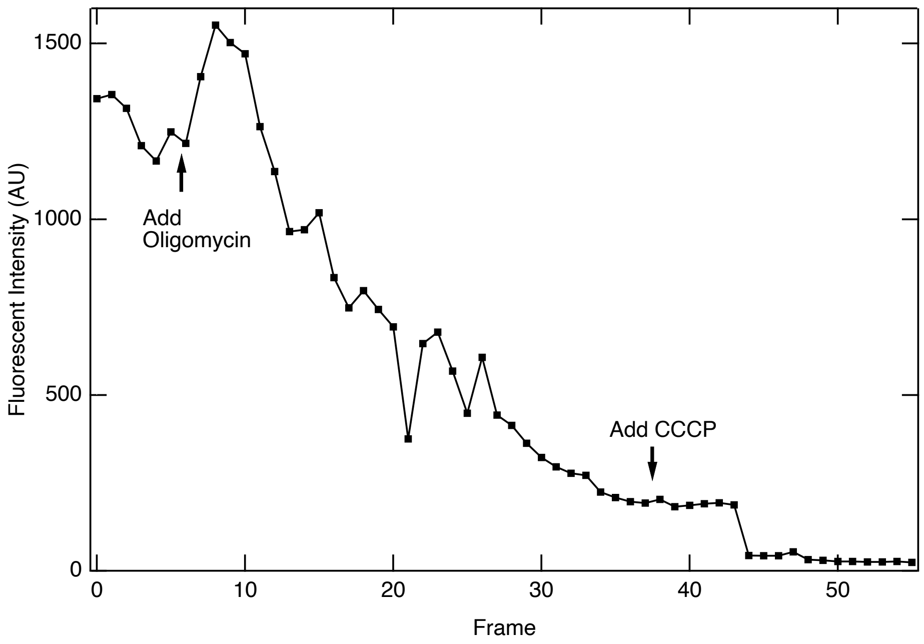

Fig. S 20. TMRE Intensity of isolated mitochondria (HEK293) treated with Oligomycin and CCCP.

Section 13: Experimental conditions: Yield and statistics

For each figure in the main text, we present a table of how many times that experiment was performed, and under what conditions. This demonstrates in detail the reproducibility of all the key findings of this paper. In particular, this clearly establishes that we are indeed seeing a common phenomenon, and not just an exception. In order to demonstrate that all conclusions drawn in the manuscript not just based on the few selected images shown, we list out the conclusion for each figure shown and the number of times that experimental conclusion was reproducible.

Conclusion for Fig. 1: "Tubular isolated mitochondria are observed which demonstrate regions of high and low cristae density when labeled with structural dyes such as NAO and mitotracker green and voltage variations when labeled with voltage dye TMRE".

In table 1, we list all the experiments on isolated mitochondria. In 26 of the 49 experiments (53%), tubular mitochondria are visible in the field of view, and were studied in more detail than circular mitochondria (discussed in more detail below), which are visible in all experiments. Typically, when tubular mitochondria are visible, they only constitute about 10% of the total # of visible mitochondria (Lee, et al, manuscript in preparation). Of the 26 tubular mitochondria experiments, in 17 of them the cristae were clearly observed (65%). The criteria to observe the cristae is that, upon performing a line profile along the axis of the tubular structure (manually aligned in ImageJ software), the peak/valley ratio exceeded 50% of mean.

Conclusion for Fig. 2B: "Voltage dye TMRE fluorescence and structure dye NAO fluorescence are correlated".

In the 17 experiments in Table 1 that showed cristae structure in tubular mitochondria, 13 of them had both a voltage and structural dye imaged. Of these 13, all of them showed correlation between the F.I. of the voltage dye and structure dye.

Conclusion for Fig. 2E: "mtDNA is anti-localized with TMRE voltage dye".

This experiment was performed 3 times, and all showed this conclusion.

Conclusion for Fig. 2F: "FCCP collapses TMRE intensity, but there is some non-zero intensity even after FCCP has collapsed the membrane voltage".

Table 2 lists these experiments. In all (6/6=100%), this effect was observed. This includes 4 different cell lines, proving it persists independent of cell line used. Additional figures confirming this are shown in Section 11 in supporting information.

Conclusion for Fig. 4A-B-C: "Changes in mitochondrial membrane potential as a function of time and position can be imaged in different metabolic states, created by the addition of substrates and inhibitors of the electron transport chain."

In Fig. 4B, we applied the respiration test to verify the function of mitochondria after isolation. This experiment was done 3 times (N=3) and 3 mitochondria were analyzed for each experiment

(total of 9 mitochondria). Among all the experiments, all 9 mitochondria showed responses to the addition of succinate (= 100%) and the responsive increase in TMRE signal is varies between 11% to 61 %. 6 out of 9 mitochondria showed responses to the addition of ADP (= 67 %) and the responsive decrease in TMRE is varies between 7 % to 29 %. Also see Section 7, 8, and 9 in supporting information for the detail analysis method and results. These are tabulated in Table 3.

As discussed in Section 8 in supporting information, we reproduced the metabolic state manipulation experiment 3 times, and in each experiment, we studied 3 individual mitochondria that happened to be within the field of view with super-resolution microscopy. All of the traces are shown in Fig. S 6 to Fig. S 13

In Fig. 4C, we used oligomycin to verify the mitochondrial function responding to inhibition of ATP synthesis. The experiment was done 4 times (N=4). Among all the experiments, 3 out of 4 times the mitochondria had responded to the addition of oligomycin (= 75% chance to show response to ATP synthesis inhibition) and the responsive increase in TMRE signal is varies between 17% to 52 %. See also Section 12 in supporting information, which shows additional reproducibility of Fig. 4C in two other cell lines, for a total of 3 cells.

Record for Isolated Mitochondria with Cristae observed (Fig 1)												
Date	Cell used (Passage #)	Microscope	Lables	Staining condition	Laser settings	Objective	Detection (nm)	Pixel time [µs]	Pixel Size [nm]	FOV [µm]	Seeing Cristae?	Shape
9/1/21	HeLa (P15)	Zeiss LSM 900<br>(Airyscan)	TMRE<br>MTG	10 nM; 15 min; DMEM<br>100 nM; 15 min; DMEM	λ_{ex}561nm (0.3%)<br>λ_{ex}488nm (0.1%)	63x	561-700<br>450-561	3.54	49	24.18x24.18	Y	circular
9/12/21	HeLa (P18)	Zeiss LSM 900<br>(Airyscan)	TMRE<br>MTG	10 nM; 15 min; DMEM<br>100 nM; 15 min; DMEM	λ_{ex}561nm (0.8%)<br>λ_{ex}488nm (0.5%)	63x	561-700<br>450-561	53.75<br>62.60	49	5.50x5.50	Y	tubular
9/13/21	HeLa (P18)	Zeiss LSM 900<br>(Airyscan)	TMRE<br>MTG	10 nM; 15 min; DMEM<br>100 nM; 15 min; DMEM	λ_{ex}561nm (1.0%)<br>λ_{ex}488nm (1.2%)	63x	561-700<br>450-561	8.24	42	6.34x6.34	Y	tubular/<br>circular
9/15/21	HeLa (P18)	Zeiss LSM 900<br>(Airyscan)	TMRE<br>MTG	10 nM; 15 min; DMEM<br>100 nM; 15 min; DMEM	λ_{ex}561nm (0.5%)<br>λ_{ex}488nm (0.5%)	63x	561-700<br>450-561	8.24	49	6.62x6.62	Y	tubular/<br>circular
9/20/21	HeLa (P7)	Zeiss LSM 900<br>(Airyscan)	TMRE<br>MTG	10 nM; 15 min; DMEM<br>100 nM; 15 min; DMEM	λ_{ex}561nm (0.7%)<br>λ_{ex}488nm (0.6%)	63x	561-700<br>450-561	16.16	49	11.64x11.64	Y	circular
9/22/21	HeLa (P8)	Zeiss LSM 900<br>(Airyscan)	TMRE<br>MTG	10 nM; 15 min; DMEM<br>100 nM; 15 min; DMEM	λ_{ex}561nm (0.7%)<br>λ_{ex}488nm (0.6%)	63x	561-700<br>450-561	10.49	49	8.26x8.26	Y	tubular
9/24/21	HeLa (P8)	Zeiss LSM 900<br>(Airyscan)	TMRE<br>MTG	10 nM; 15 min; DMEM<br>100 nM; 15 min; DMEM	λ_{ex}561nm (0.2%)<br>λ_{ex}488nm (0.6%)	63x	561-700<br>450-561	9.81	49	9.40x9.40	Y	circular
9/27/21	HeLa (P9)	Zeiss LSM 900<br>(Airyscan)	TMRE<br>MTG	10 nM; 15 min; DMEM<br>100 nM; 15 min; DMEM	λ_{ex}561nm (0.7%)<br>λ_{ex}488nm (0.2%)	63x	561-700<br>450-561	5.24	42	7.55x7.55	Y	tubular
9/28/21	HeLa (P9)	Zeiss LSM 900<br>(Airyscan)	TMRE<br>MTG	10 nM; 15 min; DMEM<br>100 nM; 15 min; DMEM	λ_{ex}561nm (0.7%)<br>λ_{ex}488nm (0.7%)	63x	561-700<br>450-561	7.11	43	11.66x11.66	Y	tubular
10/4/21	HeLa (P10)	Zeiss LSM 900<br>(Airyscan)	TMRE<br>NAO	10 nM; 15 min; DMEM<br>100 nM; 15 min; DMEM	λ_{ex}561nm (0.5%)<br>λ_{ex}488nm (0.5%)	63x	561-700<br>450-561	14.21	43	11.66x11.66	N	circular
10/15/21	HeLa (P13)	Zeiss LSM 900<br>(Airyscan)	TMRE<br>NAO	10 nM; 15 min; DMEM<br>100 nM; 15 min; DMEM	λ_{ex}561nm (0.4%)<br>λ_{ex}488nm (0.3%)	63x	561-700<br>450-561	2.61	42	6.34x6.34	N	circular
10/20/21	HeLa (P14)	Zeiss LSM 900<br>(Airyscan)	TMRE	10 nM; 15 min; DMEM	λ_{ex}561nm (10%)	63x	561-700	7.83	47	3.19x3.19	Y	circular
11/5/21	HeLa (P7)	Zeiss LSM 900<br>(Airyscan)	TMRE<br>MTG	10 nM; 15 min; DMEM<br>100 nM; 15 min; DMEM	λ_{ex}561nm (1.0%)<br>λ_{ex}488nm (0.5%)	63x	561-700<br>450-561	5.31	42	16.90x16.90	N	circular
11/13/21	HeLa (P8)	Zeiss LSM 900<br>(Airyscan)	TMRE<br>NAO	10 nM; 15 min; DMEM<br>100 nM; 15 min; DMEM	λ_{ex}561nm (5.0%)<br>λ_{ex}488nm (1.0%)	63x	561-700<br>450-561	3.92	43	7.43x7.43	Y	circular
11/30/21	HeLa (P10)	Zeiss LSM 900<br>(Airyscan)	TMRE<br>NAO	10 nM; 15 min; DMEM<br>100 nM; 15 min; DMEM	λ_{ex}561nm (5.0%)<br>λ_{ex}488nm (1.0%)	63x	561-700<br>450-561	3.92	43	8.45x8.45	Y	tubular/<br>circular
12/3/21	HeLa (P11)	Zeiss LSM 900<br>(Airyscan)	TMRE<br>MTG	10 nM; 15 min; DMEM<br>100 nM; 15 min; DMEM	λ_{ex}561nm (2.0%)<br>λ_{ex}488nm (0.2%)	63x	561-700<br>450-561	3.27	43	10.14x10.14	Y	tubular
1/7/22	HeLa (P9)	Zeiss LSM 900<br>(Airyscan)	TMRE	10 nM; 15 min; DMEM	λ_{ex}561nm (2.0%)	63x	561-700	0.85	46	10.14x10.14	Y	tubular
1/10/22	HeLa (P10)	Zeiss LSM 900<br>(Airyscan)	TMRE<br>MTG	10 nM; 15 min; DMEM<br>100 nM; 15 min; DMEM	λ_{ex}561nm (5.0%)<br>λ_{ex}488nm (4.5%)	63x	561-700<br>450-561	1.31	43	11.66x11.66	N	circular
1/16/22	HeLa (P11)	Zeiss LSM 900<br>(Airyscan)	TMRE<br>MTG	10 nM; 15 min; DMEM<br>100 nM; 15 min; DMEM	λ_{ex}561nm (1.0%)<br>λ_{ex}488nm (0.3%)	63x	561-700<br>450-561	2.22	43	10.14x10.14	Y	circular
2/3/22	HeLa (P13)	Zeiss LSM 900<br>(Airyscan)	TMRE<br>MTG	10 nM; 15 min; DMEM<br>100 nM; 15 min; DMEM	λ_{ex}561nm (2.0%)<br>λ_{ex}488nm (3.0%)	63x	561-700<br>450-561	1.15	43	76.99x76.99	N	circular
2/4/22	HeLa (P13)	Zeiss LSM 900<br>(Airyscan)	TMRE	10 nM; 15 min; DMEM	λ_{ex}561nm (0.5%)	63x	561-700	4.07	49	12.68x12.68	Y	tubular
2/8/22	L6 (P6)	Zeiss LSM 900<br>(Airyscan)	TMRE<br>MTG	10 nM; 15 min; DMEM<br>100 nM; 15 min; DMEM	λ_{ex}561nm (1.0%)<br>λ_{ex}488nm (1.0%)	63x	561-700<br>450-561	3.72	43	10.14x10.14	Y	tubular
2/11/22	HeLa (P16)	Zeiss LSM 900<br>(Airyscan)	TMRE<br>MTG	10 nM; 15 min; DMEM<br>100 nM; 15 min; DMEM	λ_{ex}561nm (1.0%)<br>λ_{ex}488nm (0.5%)	63x	561-700<br>450-561	10.66	43	8.44x8.44	Y	tubular
2/14/22	HeLa (P17)	Zeiss LSM 900<br>(Airyscan)	TMRE<br>MTG	10 nM; 15 min; DMEM<br>100 nM; 15 min; DMEM	λ_{ex}561nm (2.0%)<br>λ_{ex}488nm (2.0%)	63x	561-700<br>450-561	3.55	43	12.68x12.68	Y	circular
3/25/22	HeLa (P7)	Zeiss LSM 900<br>(Airyscan)	TMRE<br>MTG	10 nM; 15 min; DMEM<br>100 nM; 15 min; DMEM	λ_{ex}561nm (3.0%)<br>λ_{ex}488nm (1.0%)	63x	561-700<br>450-561	4.45	43	10.14x10.14	N	circular
3/30/22	HeLa (P7)	Zeiss LSM 900<br>(Airyscan)	TMRE<br>MTG	10 nM; 15 min; DMEM<br>100 nM; 15 min; DMEM	λ_{ex}561nm (3.0%)<br>λ_{ex}488nm (1.0%)	63x	561-700<br>450-561	1.15	43	78.01x78.01	N	circular
4/1/22	HeLa (P7)	Zeiss LSM 900<br>(Airyscan)	TMRE<br>MTG	10 nM; 15 min; DMEM<br>100 nM; 15 min; DMEM	λ_{ex}561nm (2.0%)<br>λ_{ex}488nm (1.0%)	63x	561-700<br>450-561	4.45	43	10.14x10.14	Y	circular
4/4/22	HeLa (P8)	Zeiss LSM 900<br>(Airyscan)	TMRE<br>MTG	10 nM; 15 min; DMEM<br>100 nM; 15 min; DMEM	λ_{ex}561nm (2.0%)<br>λ_{ex}488nm (3.0%)	63x	561-700<br>450-561	8.13	41	5.32x5.32	Y	circular
4/8/22	HeLa (P9)	Zeiss LSM 900<br>(Airyscan)	TMRE<br>MTG	10 nM; 15 min; DMEM<br>100 nM; 15 min; DMEM	λ_{ex}561nm (4.0%)<br>λ_{ex}488nm (3.0%)	63x	561-700<br>450-561	8.13	41	4.34x4.34	Y	circular
4/13/22	HeLa (P10)	Zeiss LSM 900<br>(Airyscan)	TMRE<br>MTG	10 nM; 15 min; DMEM<br>100 nM; 15 min; DMEM	λ_{ex}561nm (2.0%)<br>λ_{ex}488nm (2.0%)	63x	561-700<br>450-561	10.66	43	8.45x8.45	N	tubular/<br>circular
4/15/22	HeLa (P10)	Zeiss LSM 900<br>(Airyscan)	TMRE<br>MTG	10 nM; 15 min; DMEM<br>100 nM; 15 min; DMEM	λ_{ex}561nm (3.0%)<br>λ_{ex}488nm (4.0%)	63x	561-700<br>450-561	14.21	42	6.43x6.43	Y	tubular/<br>circular
4/25/22	HeLa (P12)	Zeiss LSM 900<br>(Airyscan)	TMRE<br>MTG	10 nM; 15 min; DMEM<br>100 nM; 15 min; DMEM	λ_{ex}561nm (1.0%)<br>λ_{ex}488nm (1.0%)	63x	561-700<br>450-561	5.22	43	6.34x6.34	Y	tubular/<br>circular
5/3/22	HeLa-GFP (P8)	Zeiss LSM 900<br>(Airyscan)	TMRE<br>GFP	10 nM; 15 min; DMEM	λ_{ex}561nm (4.0%)<br>λ_{ex}488nm (4.0%)	63x	561-700<br>450-561	1.79	42	4.06x4.06	Y	tubular
5/3/22	L6 (P8)	Zeiss LSM 900<br>(Airyscan)	TMRE<br>MTG	10 nM; 15 min; DMEM<br>100 nM; 15 min; DMEM	λ_{ex}561nm (4.0%)<br>λ_{ex}488nm (4.0%)	63x	561-700<br>450-561	2.15	42	3.38x3.38	N	tubular/<br>circular
5/11/22	L6 (P9)	Zeiss LSM 900<br>(Airyscan)	TMRE<br>PicoGreen	10 nM; 15 min; DMEM<br>3 ul/ml; 15 min; DMEM	λ_{ex}561nm (4.0%)<br>λ_{ex}488nm (1.0%)	63x	561-700<br>450-561	2.3	42	6.34x6.34	Y	tubular
5/14/22	HeLa (P12)	Zeiss LSM 900<br>(Airyscan)	TMRE<br>MTG	10 nM; 15 min; DMEM<br>100 nM; 15 min; DMEM	λ_{ex}561nm (4.0%)<br>λ_{ex}488nm (4.0%)	63x	561-700<br>450-561	2.3	42	6.34x6.34	Y	tubular
5/18/22	HeLa (P13)	Zeiss LSM 900<br>(Airyscan)	TMRE<br>MTG	10 nM; 15 min; DMEM<br>100 nM; 15 min; DMEM	λ_{ex}561nm (4.0%)<br>λ_{ex}488nm (4.0%)	63x	561-700<br>450-561	2.3	42	6.34x6.34	Y	circular
6/6/22	HeLa (P15)	STED	TMRE	10 nM; 15 min; DMEM	λ_{ex}561nm (10%)	100x	561-700	10	18	4.00x5.00	N	circular
6/13/22	HeLa (P16)	Zeiss LSM 900<br>(Airyscan)	TMRE<br>MTG	10 nM; 15 min; DMEM<br>100 nM; 15 min; DMEM	λ_{ex}561nm (1.0%)<br>λ_{ex}488nm (4.0%)	63x	561-700<br>450-561	1.43	43	5.07x5.07	Y	circular
6/20/22	HeLa (P18)	Zeiss LSM 900<br>(Airyscan)	MTDR	10 nM; 15 min; DMEM	λ_{ex}641nm (4.0%)	63x	642-700	1.5	56	5.00x5.00	Y	tubular/<br>circular
6/21/22	HeLa (P19)	Zeiss LSM 900<br>(Airyscan)	TMRE	10 nM; 15 min; DMEM	λ_{ex}561nm (4.0%)	63x	561-700	1.32	49	12.68x12.68	Y	tubular/<br>circular
6/23/22	HeLa (P19)	Zeiss LSM 900<br>(Airyscan)	TMRE	10 nM; 15 min; DMEM	λ_{ex}561nm (5.0%)	63x	561-700	0.84	49	24.18x24.18	Y	circular
6/28/22	HeLa (P20)	Zeiss LSM 900<br>(Airyscan)	TMRE	10 nM; 15 min; DMEM	λ_{ex}561nm (2.0%)	63x	561-700	2.65	49	39.00x39.00	Y	circular
7/18/22	HeLa (P23)	Zeiss LSM 900<br>(Airyscan)	TMRE	10 nM; 15 min; DMEM	λ_{ex}561nm (5.0%)	63x	561-700	1.32	49	78.01x78.01	N	circular
12/17/22	MB231 (P9)	Zeiss LSM 900<br>(Airyscan)	TMRE<br>MTG	10 nM; 15 min; DMEM<br>100 nM; 15 min; DMEM	λ_{ex}561nm (0.8%)<br>λ_{ex}488nm (2.0%)	63x	561-700<br>450-561	2	49	3.54x5.45	Y	tubular
12/17/22	HEK293 (P5)	Zeiss LSM 900<br>(Airyscan)	TMRE<br>MTG	10 nM; 15 min; DMEM<br>100 nM; 15 min; DMEM	λ_{ex}561nm (2.0%)<br>λ_{ex}488nm (2.0%)	63x	561-700<br>450-561	1.44	49	3.24x3.28	Y	tubular/<br>circular
12/26/22	HEK293 (P6)	Zeiss LSM 900<br>(Airyscan)	TMRE	10 nM; 15 min; DMEM	λ_{ex}561nm (1.0%)	63x	561-700<br>450-561	1.32	49	11.5x11.5	Y	tubular
12/27/22	HEK293 (P6)	Zeiss LSM 900<br>(Airyscan)	TMRE	10 nM; 15 min; DMEM	λ_{ex}561nm (2.0%)	63x	561-700<br>450-561	0.97	49	6.99x6.70	Y	tubular
12/28/22	HK2 (P3)	Zeiss LSM 900<br>(Airyscan)	TMRE	10 nM; 15 min; DMEM	λ_{ex}561nm (1.0%)	63x	561-700<br>450-561	0.97	49	37.83x37.83	Y	tubular

Table 1. Record and statistic table for Fig. 1. Within a total of 49 isolated mitochondria experiments, 38 of them shows cristae structure in isolated mitochondria with TMRE dye (78 %), and 26 of them sustained in tubular shape (53 %). Within a total of 26

tubular shape isolated mitochondria experiments, 17 of them were able to show cristae morphology through line profiling (65 %).

Record for Isolated Mitochondria Treat with CCCP (Fig 2F)												
Date	Cell used (Passage #)	Microscope	Lables	Staining condition	Laser settings	Objective	Detection (nm)	Pixel time [µs]	Pixel Size [nm]	FOV [µm]	Seeing Cristae?	Shape
5/11/22	L6 (P9)	Zeiss LSM 900 (Airyscan)	TMRE<br>MTG	10 nM; 15 min; DMEM<br>100 nM; 15 min; DMEM	λ_{ex}561nm (4.0%)<br>λ_{ex}488nm (1.0%)	63x	561-700<br>450-561	2.3	42	6.34x6.34	Y	tubular
12/26/22	MB231(P11)	Zeiss LSM 900 (Airyscan)	TMRE	10 nM; 15 min; DMEM	λ_{ex}561nm (4.0%)	63x	561-700	0.85	49	16.90x16.90	Y	tubular
12/26/22	HEK293 (P6)	Zeiss LSM 900 (Airyscan)	TMRE	10 nM; 15 min; DMEM	λ_{ex}561nm (1.0%)	63x	561-700	1.32	49	11.5x11.5	Y	tubular
12/27/22	HEK293 (P6)	Zeiss LSM 900 (Airyscan)	TMRE	10 nM; 15 min; DMEM	λ_{ex}561nm (2.0%)	63x	561-700	0.97	49	3.52x3.47	Y	tubular<br>circular
12/28/22	HK2 (P3)	Zeiss LSM 900 (Airyscan)	TMRE	10 nM; 15 min; DMEM	λ_{ex}561nm (1.0%)	63x	561-700	0.97	49	39.00x39.00	Y	tubular
12/28/22	HK2 (P3)	Zeiss LSM 900 (Airyscan)	TMRE	10 nM; 15 min; DMEM	λ_{ex}561nm (2.0%)	63x	561-700	0.84	49	2.98x2.93	Y	tubular

Table 2. Record and statistic table for Fig. 3. We've examined the line profile of isolated mitochondria before and after treatment with CCCP (N=6, with 4 different kinds of cell lines).

Record for Isolated Mitochondria Respiration Experiment (Fig 4A)												
Date	Cell used (Passage #)	Lables	Laser settings	Detection (nm)	Pixel time [µs]	Pixel Size [nm]	FOV [µm]	Shape	Percentage increase due to succinate	Two Drops by ADP addition	Percentage of 1st drop	Percentage of 2nd drop
06/21/22 mito1	HeLa (P19)	TMRE	λ_{ex}561nm (4.0%)	561-700	1.32	49	12.68x12.68	tubular/ circular	11%	Y	17%	21%
6/21/22 mito2	HeLa (P19)	TMRE	λ_{ex}561nm (4.0%)	561-700	1.32	49	12.68x12.68	tubular/ circular	24%	Y	8%	7%
6/21/22 mito3	HeLa (P19)	TMRE	λ_{ex}561nm (4.0%)	561-700	1.32	49	12.68x12.68	tubular/ circular	16%	Y	13%	11%
6/23/22 mito1	HeLa (P19)	TMRE	λ_{ex}561nm (5.0%)	561-700	0.84	49	24.18x24.18	circular	46%	Y	29%	19%
6/23/22 mito2	HeLa (P19)	TMRE	λ_{ex}561nm (5.0%)	561-700	0.84	49	24.18x24.18	circular	39%	N	N/A	N/A
6/23/22 mito3	HeLa (P19)	TMRE	λ_{ex}561nm (5.0%)	561-700	0.84	49	24.18x24.18	circular	61%	N	N/A	N/A
6/28/22 mito1	HeLa (P20)	TMRE	λ_{ex}561nm (2.0%)	561-700	2.65	49	39.00x39.00	circular	16%	Y	20%	19%
6/28/22 mito2	HeLa (P20)	TMRE	λ_{ex}561nm (2.0%)	561-700	2.65	49	39.00x39.00	circular	21%	N	N/A	N/A
6/28/22 mito3	HeLa (P20)	TMRE	λ_{ex}561nm (2.0%)	561-700	2.65	49	39.00x39.00	circular	15%	Y	13%	11%

Table 3. Record and statistic table for Fig. 4A. The rate of mitochondria showing response to ADP addition is 67 %. The percentage drop of TMRE intensity varies between mitochondria from 7 % to 29 % (N=3).

Record for Isolated Mitochondria Treat with Oligomycin (Fig 4C)											
Date	Cell used (Passage #)	Microscope	Lables	Staining condition	Laser settings	Detection (nm)	Pixel time [µs]	Pixel Size [nm]	FOV [µm]	Response to Oligomycin	Percentage of Change
5/11/22	L6 (P9)	Zeiss LSM 900 (Airyscan)	TMRE<br>MTG	10 nM; 15 min; DMEM<br>100 nM; 15 min; DMEM	λ_{ex}561nm (4.0%)<br>λ_{ex}488nm (1.0%)	561-700<br>450-561	2.3	42	6.34x6.34	Y	52%
12/26/22	MB231(P11)	Zeiss LSM 900 (Airyscan)	TMRE	10 nM; 15 min; DMEM	λ_{ex}561nm (4.0%)	561-700	0.85	49	16.90x16.90	N	N/A
12/27/22	HEK293 (P6)	Zeiss LSM 900 (Airyscan)	TMRE	10 nM; 15 min; DMEM	λ_{ex}561nm (2.0%)	561-700	0.97	49	3.52x3.47	Y	17%
12/28/22	HK2 (P3)	Zeiss LSM 900 (Airyscan)	TMRE	10 nM; 15 min; DMEM	λ_{ex}561nm (1.0%)	561-700	0.97	49	39.00x39.00	Y	24%

Table 4. Record and statistic table for Fig. 4C. The rate of mitochondria showing response to Oligomycin addition is 75%. The percentage change of TMRE intensity varies between mitochondria from 17 % to 52 % (N=4).

Record for Seahorse Experiment								
Date	Cell used (Passage #)	Instrument	Chemical Addition	Response to ADP	Response to Oligomycin	Response to CCCP	Response to Rotenone	Respiratory Control Ratio (RCR)
10/17/19	HeLa cell (P12)	Seahorse XFe24	55 µL, 40 mM ADP; 60 µL, 25 µg/mL oligomycin, 65 µL, 40 µM FCCP;	Y	Y	Y	N/A	2.37
1/5/22	Mouse Heart	Seahorse XFe24	55 µL, 40 mM ADP; 60 µL, 25 µg/mL oligomycin, 65 µL, 40 µM FCCP; 70 µL, 40 µM Rotenone	Y	Y	y	Y	3.9

Table 5. Record and statistic table for the Seahorse experiment. We've done the respiration test on both isolated mitochondria from HeLa cells and the mouse heart. The Respiratory Control Ratio (RCR) was 2.37 and 3.9, respectively (N=1).

Record for Isolated Mitochondria with STED											
Date	Cell used (Passage #)	Lables	Staining condition	Laser settings	Objective	Detection (nm)	Pixel time [µs]	Pixel Size [nm]	FOV [µm]	Seeing Cristae?	Shape
6/6/22	HeLa (P15)	TMRE	10 nM; 15 min; DMEM	λ_{ex}561nm (10%)	100x	561-700	10	18	4.00x5.00	N	circular
6/6/22	HeLa (P15)	MTDR	10 nM; 15 min; DMEM	λ_{ex}641nm (4.0%)	100x	642-700	10	15	34.00x34.00	Y	circular

Table 6. Record and statistic table for isolated mitochondria observed by STED microscope. We were able to resolve mitochondria cristae with MTDR but not TMRE (N=1).

Section 14: Relationship to Murphy et al [16]

In the main text, we have clarified that, even under zero potential, there is still TMRE bound to the membrane. One could in principle call the binding to the outer membrane (buffer or cytosol side) potential independent binding. Only the TMRE bound to the inner membrane (matrix side) is potential dependent, since the free TMRE in the matrix is potential dependent, and the amount bound on the matrix side is proportional to the free TMRE in the matrix. We have also referenced key previous literature that deals with this.

Murphy studied "potential independent binding" extensively, for example, ref [16]. His cartoon (Fig. 1A) is identical to our cartoon (Fig. 3A), in principle, which again is equivalent to Rottenberg 1984 which we cited in our original draft manuscript. In Murphy's work, he studied the "proportion of cation that is membrane bound" for different TPP^+ conjugates. The conjugates of various linker lengths, up to 15 carbon atoms, became gradually more hydrophobic as the linker length increased. Therefore, he found, the "proportion of cation that is membrane bound" became gradually larger and larger as the linker length was increased by his lab and students.

The relationship to our model presented In Fig. 3 in the main text is as follows: Murphy essentially studied the constant "a_i", which in our model is the binding coefficient between the bound and free TMRE in the matrix. Murphy called this the "proportion of cation that is membrane bound" in the matrix. Murphy found, logically, that the more hydrophobic the moiety was, the larger the value of a_i, i.e. the larger the fraction bound to the membrane. In our work, we used TMRE which has a known and measured value of a_i from the literature, measured by many groups for TMRE. Whereas, Murphy measured that binding coefficient for other species of lipophilic cations that, while related in structure to the TMRE used in this work, are chemically different. Therefore, Murphy's binding coefficient measurements in ref [16] are not directly applicable to our work. The way the value of a_i was measured for TMRE by other groups is discussed in detail in Section 3 in supporting information above.

Note, for Murphy, a_o also increased with the hydrophobicity of his compounds, so that his compounds also bound to the outer membrane with increasing binding coefficient as a function of

hydrophobicity, to the point where in extreme cases, he observed significant "uptake" even with no membrane voltage.

However, it is important to note that, no matter what value of binding coefficient (a_i, a_o), there is ALWAYS some membrane binding. It is just not measurable in all "uptake" experiments if the voltage is zero and (a_i, a_o) are small. This is why we initially did not notice it in our super-resolution experiments, until we re-examined the images and, lo and behold, even at zero voltage (CCCP collapsed), we were able to image the bound TMRE and show it had structure related to the cristae and mitochondrial internal ultrastructure (Fig. 2F).

Section 15: Biological Implications: Disease and health in mitochondria.

The internal ultra-structure of mitochondria has long and recently been known to be important in health and disease, including stem cell biology [17], cancer [18], and aging [19]. Our work extends the ultrastructure studies to functional studies, allowing the superresolution electrophysiology of mitochondria to be directly tested in response to pharmacological manipulation of metabolism for future studies in aging, disease, and health. Summarizing: "*Novel research on the functional relevance of compartmentalization has highlighted a key role of regulated cristae subcompartment structure in bioenergetics and in human diseases.*" [20]

The topic of mitochondria ultrastructure has been studied with TEM and on fixed cells extensively because of new findings about the relevance to apoptosis and metabolism [21–26]. It is not just the ultrastructure: the membrane potential (bioelectronic property) also plays a critical role in the physiology of the organelle & cell: "*Cristae dynamics and local changes in mitochondrial membrane potential at the level of individual cristae are predicted to have major consequences for mitochondrial functions such as oxidative phosphorylation, thermogenesis,* $Ca2^{+}$ homeostasis, and apoptosis." [27]

Outstanding questions that the methods and techniques presented in this work can help to address in future studies are: What are the energy requirements of cristae membrane dynamics? [27] Do other molecular players regulate cristae membrane dynamics? [27] How are cristae dynamics affected during apoptosis? [27] How do cristae dynamics affect the superresolution organization of redox nanodomains and redox signaling? [27] What is the role of cristae architecture in mitochondrial bioenergetics? [20]

For example, what might be the difference between interfibrillar and subsarcolemmal mitochondria in muscle cells? What is the functional difference between cerebellar Purkinje vs. granular cell mitochondria? In a cell harboring a heteroplasmic mitochondrial DNA disease mutation, are the mitochondria physiologically different depending on their resident mitochondrial DNA genotype or are all of the mitochondria functionally similar due to homogenization by fusion and fission? Are mitochondria undergoing mitophagy (a membrane potential dependent phenomenon) physiologically different from the mitochondria that will be retained?

Note that this manipulation of metabolic states in state 3 and state 4 is not possible in whole cells, and so this clearly demonstrates a significant advance above the already significant live cell imaging of TMRE in whole cells in ref. [28].

Section 16: Spatial and temporal statistical properties and mitochondrial heterogeneity

Prior to this work (and the advent of super-resolution microscopy), researchers were only able to experimentally measure the total amount of TMRE taken up by mitochondria, without resolving where within the organelle it was located. In practice, the entire organelle would appear as one voxel/pixel, and the intensity so measured of the voxel was the average fluorescence intensity, and hence, proportional to the average TMRE density inside the mitochondria. This work shows for the first time the evolution of the electrophysiology at the superresolution scale as the mitochondria translate between different metabolic states.

Mitochondrial DNA exhibits heterogeneity from one organelle to the next, even within a single cell, a phenomenon termed heteroplasmy: Each individual mitochondria carries a different DNA copy [29,30]. Therefore, functional differences between mitochondria are to be expected. We term this "functional heterogeneity". In orthogonal measurements under identical conditions, we performed TPP^+ analysis of the membrane potential [31,32]. If we compare the effect of succinate to the membrane potential change, the result from the TPP^+ test (calculated ~ 27 % increase) is comparable to our data in this manuscript (11 % – 24 %). Although our studies of single mitochondria are consistent with the ensemble membrane potential studies using TPP^+ electrodes [31], it should be expected that there is some spread in the membrane potential of one mitochondrion to the next, discussed next.

In our work, we reproduced the metabolic state manipulation experiment 3 times, and in each experiment, we studied 3 individual mitochondria that happened to be within the field of view with super-resolution microscopy. With this sample size of 9, we found 66 % of the mitochondria demonstrated a change in average membrane potential upon state 3/state 4 transitions. The change was between 5 % – 20 %. The overall average membrane potential of each mitochondrion varied from one mitochondrion to the next. Although 9 is a small sample size, this is a demonstration of functional heterogeneity plasmy in isolated mitochondria. Although beyond the scope of this paper, scaling this to 96 well plates or even microfluidic chambers could enable massively parallel interrogation of mitochondrial functional heteroplasmy.

Our measurements also enable quantitative measurement of both the spatial and temporal variation of the membrane potential within a single, individual mitochondrion. The spatial variation of the mitochondrial membrane potential was calculated as the ratio of the standard deviation of the fluorescent intensity (FI) inside the ROI divided by the average FI inside the ROI. The ROI is the mitochondria (see SI). During the metabolic manipulation, we found the variation to be about 60 % of the average FI and did not change significantly during the transition from state 3 to state 4 (Fig. S 14). As for the temporal variation, we calculated the average intensity at each interrogation point and this shows some variation of order 10 % of the total fluorescence intensity (hence potential) as a function of time. While this could be partially from instrumental noise, this is an upper limit on fluctuation on the second timescale of the average mitochondrial membrane potential. This is the first proof of concept use of super-resolution microscopy to determine spatial and temporal fluctuations of the mitochondrial membrane potential along a single, isolated mitochondrion. Clearly, improvements in the spatial and temporal resolution will be an intriguing avenue for future research, enabling potentially for the first time to ask the question of whether there is a Hogdkins-Huxley equivalent model (which we would term the Burke-Wallace model) of mitochondrial membrane dynamics [33].

Section 17: Resolution discussion

It is absolutely true we cannot resolve which side of the inner membrane the TMRE is localized to with the given setup. In fact, to resolve that, one would need to resolve with better resolution than the size of the lipid bilayer (~ 4 nm), which is beyond the capability of any live cell microscopy technique.

However, experiments over 40 years on liposomes have shown lipophilic cations (such as TMRE) are taken INTO the liposome in a voltage dependent manner. Furthermore, they have shown by spin EPR studies and other studies that after it is taken up, it binds to the inside of the liposome membrane[34–36]. Furthermore, many experiments over the last 40 years[2–4,31,32,34–41] have shown that lipophilic cations such as TMRE are taken up by mitochondria INTO the matrix when it is fed substrates and inhibitors of the electron transport chain, and that it is released back into the buffer/cytosol when the membrane potential is shorted to zero with FCCP. So, there is plenty of evidence that TMRE is taken up INTO the mitochondria matrix in response to a membrane potential. Nobody doubts this, and nobody has any reason to doubt it, even though they cannot see it. Our results confirm this finding that has been repeated thousands of times around the world. There is also an excellent model based on the Nernst equation that supports this [2–4,31,32,34–41]. Therefore, our work is the first to show experimentally in vital isolated mitochondria that the TMRE is localized near the cristae, and the models to date showed indirectly that they are localized on the inside, not outside, i.e. matrix side of the inner membrane.

Notice that voltage drop is across the inner membrane, which is folded (see Fig. 1A). The outer membrane is completely porous to small and even medium sized molecules.

Furthermore, the outer membrane is sometimes parallel to the inner membrane (see Fig. 1), and the space between them (called the "intermembrane space") is typically less than 20 nm. So in some images where the outer membrane is seen (e.g. Fig. S1B), it is typically so close to the parallel inner membrane that both are imaged within the resolution of the microscope.

Section 18: Comparison between Airyscan, STED, and Lattice SIM microscopy

Airyscan and STED were described and investigated for voltage imaging above. In this section, we provide data from Lattice SIM (Structured Illumination Microscopy) for voltage imaging with TMRE.

In Lattice SIM, the sample area is illuminated with a lattice spot pattern instead of grid lines as in conventional SIM. This leads to a higher imaging speed, higher contrast, and less bleaching of fluorophores, which makes it suitable for time-series studies in bio samples [42]. Noted that Lattice SIM microscopy can achieve imaging at fast frame rates, typically ranging from several frames per second to several tens of frames per second, depending on the resolution and the sample. In principle, Lattice SIM with SIM^2 processing can provide with a resolution of down to 60 nm. However, with preliminary experiments, when using TMRE (Elyra 7 Lattice SIM with SIM^2 processing), we found a resolution of around 200 nm (using the FWHM of a line profile of the smallest observable feature in Fig. S 21), much worse than STED or Airyscan for voltage imaging. Therefore, we did not pursue this imaging method.

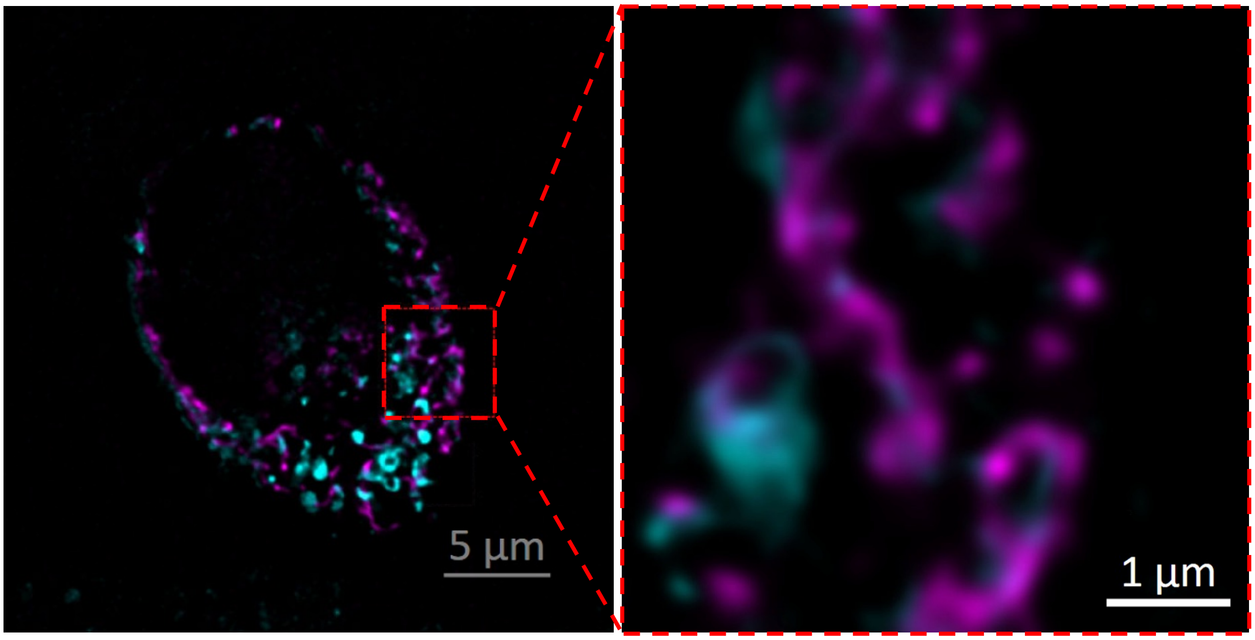

Fig. S 21. Lattice SIM image of mitochondria in HeLa cells stained with mitotracker green (blue) and TMRE (purple)

Section 19: Wide view of isolated mitochondria: "Zoom out" vs. "Zoom in"

We are limited to about an hour of imaging since the mitochondria die about an hour after isolation. In addition, when we "zoom out" (e.g. 6-10x larger field of view), we are limited in resolution and cannot resolve internal microstructure. Therefore, instead of doing many "zoom" images on one sample, we were forced to do many individual experiments over the course of 2 years to build up meaningful statistics, as shown in Section 13 in supporting information. This does not change the findings of the paper.

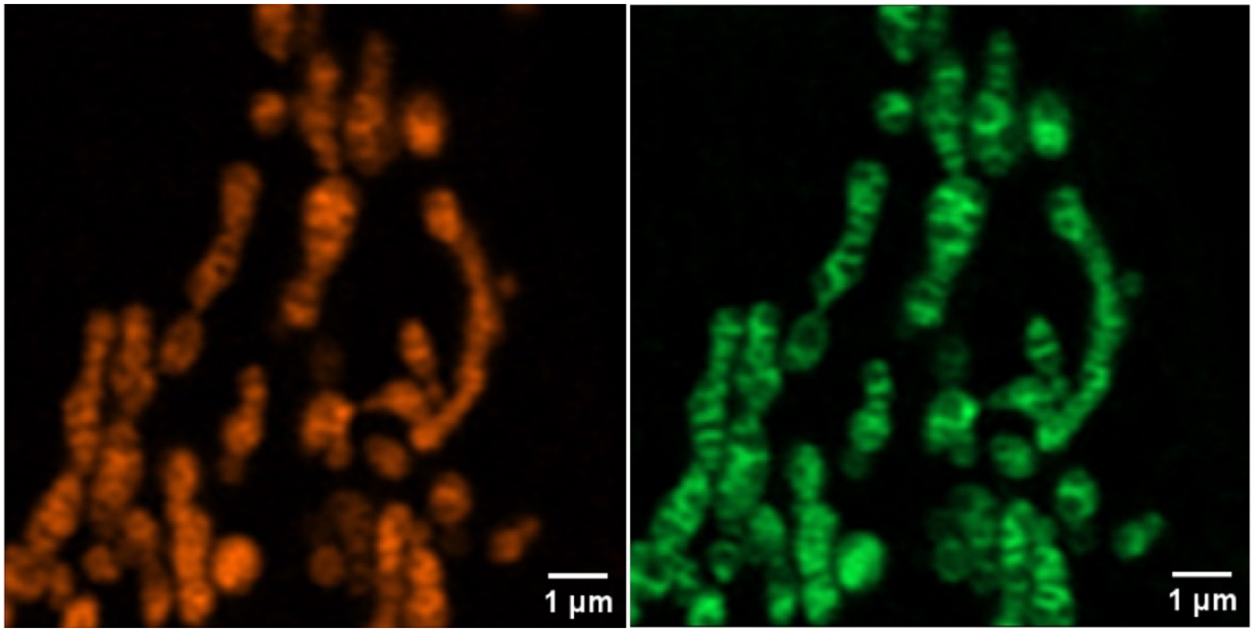

Fig. S 22. The wide-view image for Fig. 2B in the manuscript.

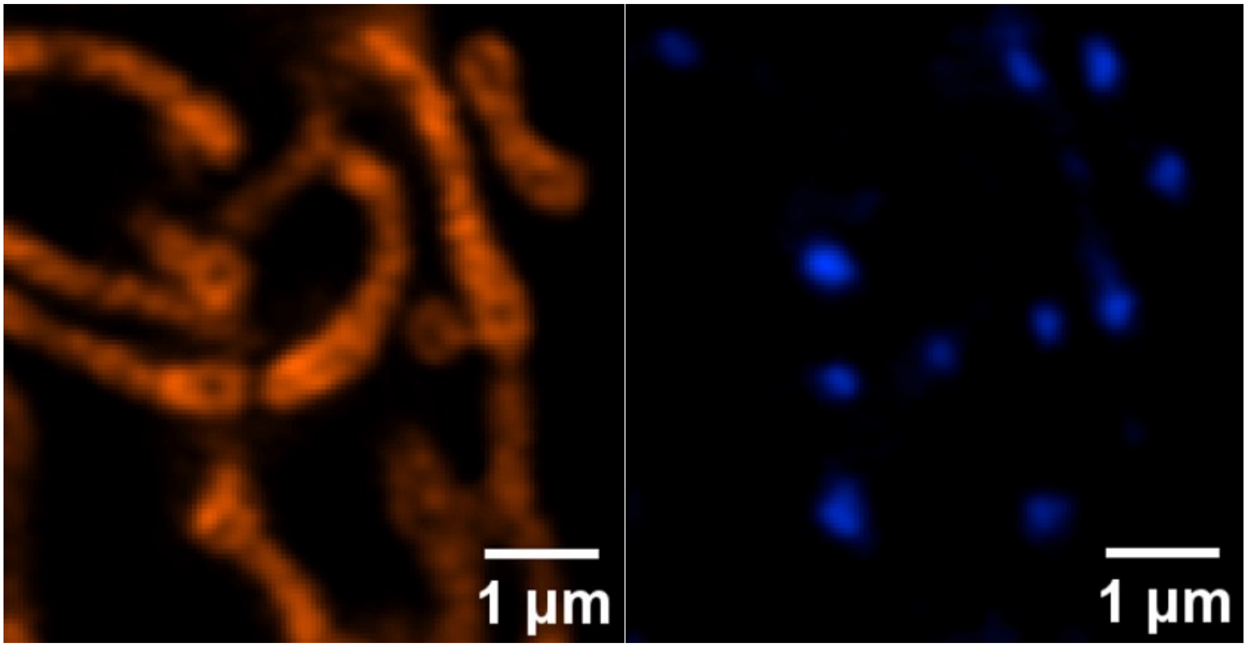

Fig. S 23. Wide-view image for Fig. 2E in the manuscript.

In this paper, we present mostly the "zoom in" images to show the inner structure of isolated mitochondria. Here we explained the reason for presenting the "zoom in" images in this draft, and present "zoom out" images for each "zoom in" image presented in the paper.

Fig. S 22 and Fig. S 23 show zoom out of mitochondria presented as zoom in in Fig. 2. Clearly, the mitochondria cristae are reproducible and the conclusions sound when zoomed out. However, these were in whole cells, where no circular mitochondria were observed when the cells were properly treated.

For isolated mitochondria, Fig. S 24 and Fig. S 25 represent typical "zoom out" images. First, we notice, as discussed above, that the number of circular mitochondria out numbers the number of tubular mitochondria. In the interest of time for this paper, we focused on the tubular mitochondria. The circulate mitochondria will be studied in a different manuscript (Lee, et al, manuscript in preparation), and are beyond the scope of this work.

Second, the internal ultrastructure of the mitochondria is difficult to resolve in the "zoom out" images. Furthermore, generating zoom out images take 10x longer (~5 seconds for each laser channel) than zoom in images (<400 ms for each laser channel), during which time motion blurring may be more significant (see "Protocols for visualization of mitochondria structure using Airyscan and STED microscopy" in the materials and methods section of the main manuscript). Therefore, instead of doing many "zoom" images on one sample, we were forced to do many individual experiments over the course of 2 years to build up meaningful statistics, as shown in the Section 13 in supporting information.

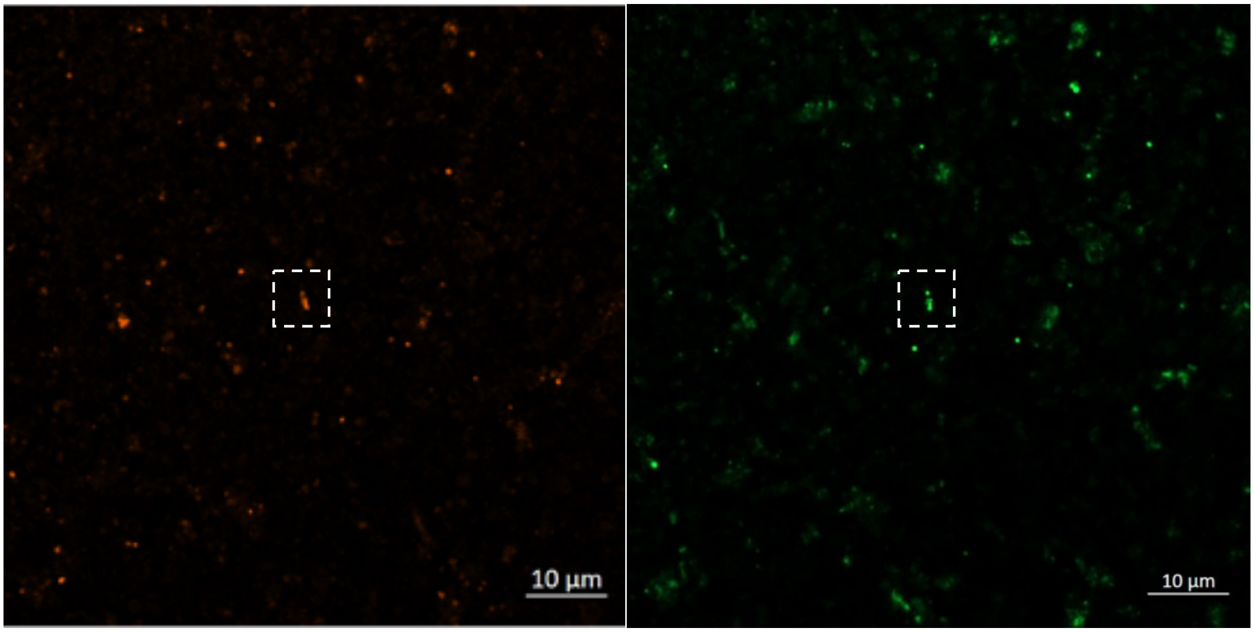

Fig. S 24. Wide-view images of isolated mitochondria stained with TMRE (orange) and MTG (green). This is the same data as Fig. 4A in the manuscript. ROI (zoom region) indicated was presented in the main text.

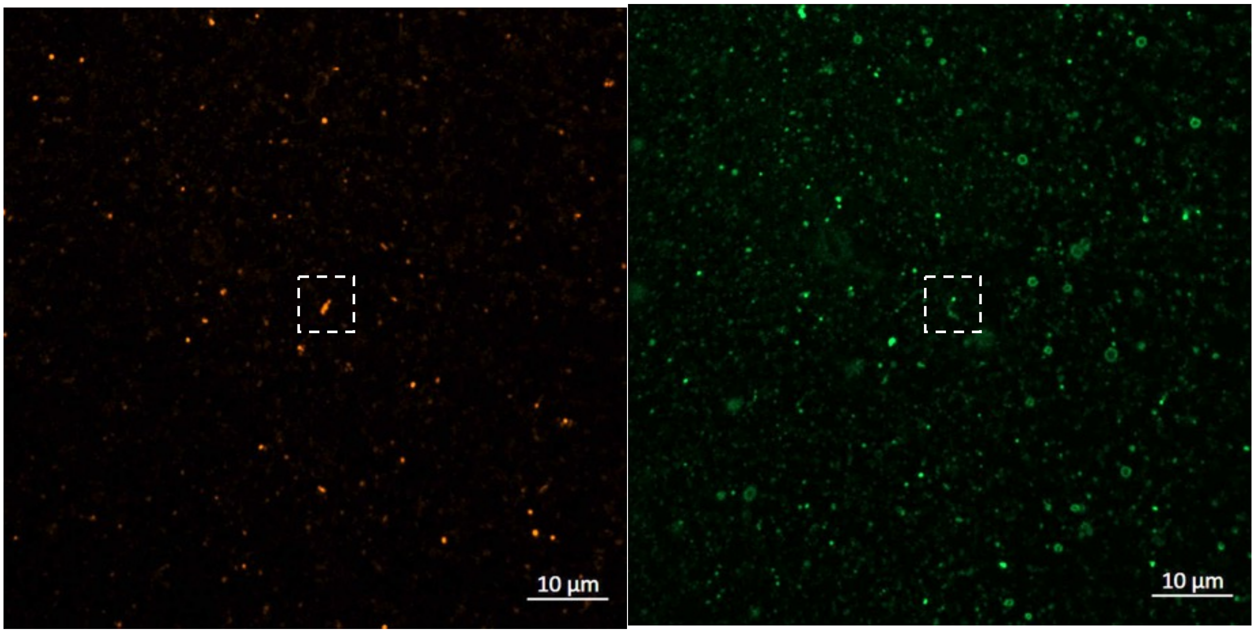

Fig. S 25. Wide-view images for 4C in the manuscript. ROI (zoom region) indicated was presented in the main text.

Section 20: Literature review for super-resolution live-cell imaging of mitochondria

Year	Author	Microscope	Image Resolution	Dye used	Structure/ Function	In cell/ Isolated	Publication
2019	Dane. W.	Airyscan	150 nm	MTG/TMRE	Structure/Voltage	In cell	Individual cristae within the same mitochondrion display different membrane potentials and are functionally independent
2019	Till. S.	STED	74 nm	SNAP-tag	Structure	In cell	Live-Cell STED Nanoscopy of Mitochondrial Cristae
2019	Chenguang. W.	STED	45 nm	MitoPB Yellow	Structure	In cell	A photostable fluorescent marker for the superresolution live imaging of the dynamic structure of the mitochondrial cristae
2020	Xusan. Y.	STED	35.2 nm	MitoESq-635	Structure	In cell	Mitochondrial dynamics quantitatively revealed by STED nanoscopy with an enhanced squaraine variant probe
2020	Yifang. S.	STED	80 nm	SiRMO	Structure	In cell	Improving Brightness and Stability of Si-Rhodamine for Super-Resolution Imaging of Mitochondria in Living Cells
2022	Tianyan. L	STED	50 nm	PKMO	Structure	In cell	Multi-color live-cell STED nanoscopy of mitochondria with a gentle inner membrane stain
	**Ours**	**Airyscan**	**120 nm**	**MTG/NAO/TMRE**	**Voltage/Respiration**	**Isolated**	

Table 7. Literature reviews of mitochondria studies using super-resolution techniques. All The literatures studied structures of mitochondria in cells. We're the only one studied isolated mitochondria and its functions.

The following Table 7 (from refs. [28, 10–13], & ref. [43], which came out while this paper was under review) listed with the name of the publication, type of super-resolution microscope use, image resolution, type of dye use, structural or functional imaging, and in cell or isolated of the recent research related to super-resolution study on mitochondria in live-cells.

Note first that all but one of these measure structure, not voltage. Therefore, "nanoscopy", i.e. sub-50 nm imaging of voltage is still not achieved anywhere in the literature.

Second, the images we have of TMRE with STED, although it is able to image structure very well (80 nm, Fig. S 1, comparable to the literature on live-cells with STED), we found that voltage imaging with STED was still limited to 140 nm.

Third, and finally, none of the work above uses isolated mitochondria. Isolated mitochondria provide advantages above whole cells in manipulating the metabolic state, as discussed in the introduction section of the main text.

A recent review also covers live-cell nanoscopy[44].

Section 21: Chemical structure of fluorescent dyes used in this manuscript

Nonyl Acridine Orange (NAO)	Tetramethylrhodamine ethyl ester, perchlorate (TMRE)	MitoTracker Green FM (MTG)
•$\lambda_{Ex}/\lambda_{Em}$ = 495/522 nm<br>•Orange solid soluble in DMSO or DMF<br>• $C_{26}H_{38}BrN_3$<br>•MW: 473	•$\lambda_{Ex}/\lambda_{Em}$ = 549/574 nm<br>•Red solid soluble in DMSO, DMF or EtOH<br>•$C_{26}H_{27}ClN_2O_7$<br>•MW: 515	•$\lambda_{Ex}/\lambda_{Em}$ = 490/516 nm<br>•Orange solid soluble in DMSO<br>•$C_{34}H_{28}Cl_5N_3O$<br>•MW: 672
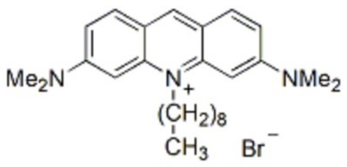	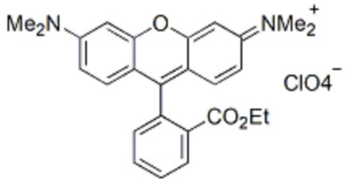	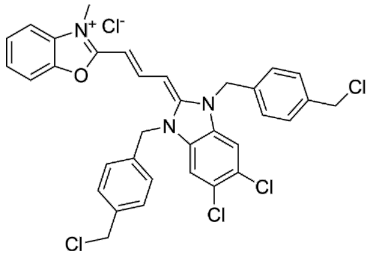

Figure references:
https://biotium.com/product/nonyl-acridine-orange-nao/
https://biotium.com/product/tetramethylrhodamine-ethyl-ester-perchlorate-tmre/
https://www.medchemexpress.com/mitotracker-green-fm.html

Section 22: Confusion on the binding abounds in the literature:

Even as recent as 2018, ref. [45] shows a misleading picture of the lipophilic cation TPP^+ is free and ignores membrane binding in the schematic and in the analysis.

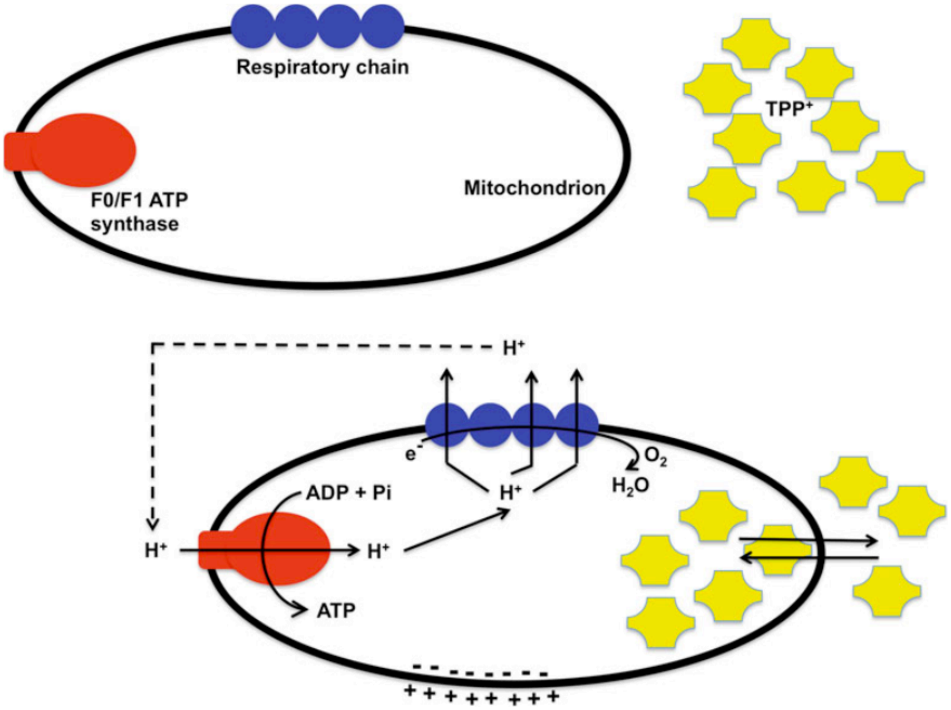

Fig. S 26. Schematic representation of TPP^+ distribution across the mitochondrial membrane, according to the Nernst equation from.[45] This example shows how in the literature, membrane binding of lipophylic cations is typically (and incorrectly) ignored. Reprinted from Methods Mol Biol, 1782, Teodoro, J. S.; Palmeira, C. M.; Rolo, A. P., Mitochondrial Membrane Potential (ΔΨ) Fluctuations Associated with the Metabolic States of Mitochondria, Pages 109–119., Copyright (2018). with permission from Springer Nature.

Our model builds on and enhances, but differs substantially from that of ref. [28]:

Quote #1 (from published review/response letter ref. [28]):

> *…our data quantifying just the signal from the **diffusible** fraction of TMRE (non-binding and thus sensitive to ΔΨm) which also responds differently in the cristae and IBM to oligomycin and FCCP, strongly supports that increased **diffusible** TMRE FI in the cristae is caused by increased ΔΨm, when compared to the IBM.*
>
> *The FIs of the probes at different subcellular compartments can be used to extrapolate the differences in **concentrations** of the probe, which are needed to calculate the difference in ΔΨm between compartments (Ehrenberg et al., 1988; Farkas et al., 1989; Loew et al., 1993; Twig et al., 2008; Wikstrom et al., 2007).”*
>
> *https://www.embopress.org/action/downloadSupplement?doi=10.15252/embj.2018101056&file=embj2018101056.reviewer_comments.pdf*

Comment:

This ignores the bound TMRE, which creates the brightest fluorescence intensity, which we showed in this manuscript. Words like diffusible and concentration refer to free, unbound TMRE which is not what is seen in the images since it is much dimmer than the bound TMRE.

Quotes #2,3 (From supplemental info [46], [47], referenced in ref. [28]):

The non ΔΨ-dependent component of TMRE, also known as the ***binding component, can be ignored*** *when calculating relative changes in ΔΨ as it is fixed and voltage-independent (2). (2=O'Reilly)*

The non-Δψm-dependent component of TMRE, also known as ***the binding component, can be ignored*** *as it is fixed and voltage-independent (Loew et al., 1993;O'Reilly et al., 2003).*

Response:

This ignores the bound TMRE on the matrix side to calculate the membrane potential, which creates the brightest fluorescence intensity, which we showed in this manuscript. It was stated in O'Reilly [38] that this needs to be taken into account, but this was not properly applied in ref. [28]'s work:

(O'Reilly et al., 2003 [38]): TMRE, even at low concentrations, exhibits significant binding to the mitochondrial membrane as found by Scaduto and Grotyohann (1999).

(O'Reilly et al., 2003 [38]): Scaduto and Grotyohann (1999) have shown that TMRE and similar dyes exhibit significant binding to the inner mitochondrial membranes so that most fluorescence comes not from free TMRE but from bound TMRE. (See eq. 1, 2 in that reference, repeated here):

TMRE, even at low concentrations, exhibits significant binding to the mitochondrial membrane as found by Scaduto and Grotyohann (1999). Hence the absolute value for $\Delta\Psi_m$ cannot be determined by a simple Nernstian relationship between $[\mathrm{TMRE}]_m$ and $[\mathrm{TMRE}]_c$, but changes in $\Delta\Psi_m$ (flicker amplitude) can be determined from changes in FI as follows. The FI in a mitochondrion is a measure of its total TMRE content, as given by

$$\{\mathrm{TMRE}\}_{\mathrm{total}} = K_i[\mathrm{TMRE}]_m + volume_m[\mathrm{TMRE}]_m + K_o[\mathrm{TMRE}]_c + volume_{im}[\mathrm{TMRE}]_c, \quad (1)$$

where $\{\mathrm{TMRE}\}_{\mathrm{total}}$ = total mitochondrial TMRE in units of mass, and $[\mathrm{TMRE}]_c$ was always 2.5 nM and includes [TMRE] in the mitochondrial intermembrane space.

The following are given in units of volume:

$volume_m$ = mitochondrial matrix volume
$volume_{im}$ = volume of the intermembrane space
K_i = partition coefficient for $[\mathrm{TMRE}]_m$ binding to the inner mitochondrial membrane
K_o = partition coefficient for $[\mathrm{TMRE}]_c$ binding to the inner mitochondrial membrane

$[\mathrm{TMRE}]_c$ is >300-fold less than $[\mathrm{TMRE}]_m$ in a mitochondrion with $\Delta\Psi_m = -150$ mV; $volume_m$ and $volume_{im}$ are small compared to K_o or K_i; the value of K_o is 129, and that of K_i is 60 at 28°C (Scaduto and Grotyohann, 1999). (This value for K_i is in precise agreement with the bound:free ratio of 63:1 which we obtained as outlined in the previous paragraph.) Hence, the above expression simplifies to

$$\{\mathrm{TMRE}\}_{\mathrm{total}} = (K_i + volume_m)[\mathrm{TMRE}]_m. \quad (2)$$

Fig. S 27. Reprinted from Biophysical Journal, 85(5), O'Reilly, C. M., Fogarty, K. E., Drummond, R. M., Tuft, R. A., & Walsh, J. v., Quantitative analysis of spontaneous mitochondrial depolarizations, Pages 3350–3357., Copyright (2003). with permission from Elsevier. [38]

In this work, we also reference Scaduto 2009 [3] as a key reference for our interpretation, which explains why the cristae TMRE fluorescence shines bright, whereas inside the matrix the TMRE fluorescence is close to zero.

In summary, Scatudo 2009 [3], OReilly 2003 [38], and our paper all clearly state that the TMRE bound to the inside is the dominant amount of TMRE, and is voltage-dependent. Our model builds on and enhances, but differs substantially from that of ref [28], which discusses the "diffusible", "non-binding" component, i.e. unbound TMRE, by including the bound component in the analysis.

However, going beyond Scatudo and OReilly, our work is the first to observe the bound component using super-resolution and correctly interpret it.

Section 23: Alternative methods to determine concentrations of dye inside and outside the mitochondria

In this paper, the lipophilic cationic dye concentrations inside and outside the mitochondria are indirectly measured via the fluorescence intensity when quantifying the voltage using the Nernst equation. In principle, this method could be problematic due to the presence of solvent sensitivity: The environment inside and outside mitochondria is different, which could affect the brightness of dyes. One might ask, is there a better way to determine the concentration of dyes inside and outside the mitochondria?

The answer is yes, absolutely, there is a better way to determine the concentration of dyes inside and outside the mitochondria. The better way consists of using an electrochemical sensor of the lipophilic cation that is specific: When the mitochondria take up or release the cation, the external (buffer) concentration can be read out. Since it is read out only in the buffer, there are no artifacts related to the different chemical environments. One only needs to carefully calibrate the system. In fact, this is commonly done in the literature, by many groups, including our own [2,31,32].

In all of the findings in the literature, the results from the external electrochemical electrode are consistent with the fluorescent dye experiments. Therefore, although the artifact that the reader might be concerned with, while in principle a completely legitimate concern, in practice has been shown not to be an issue.

Section 24: Relationship to Miller et al [48]

As we mentioned in the paper, TMRE is a "tried and true" dye that has been extensively used for over 20 years without artifacts by the mitochondria community. That said, it is always a good idea to investigate new dyes that are faster, more sensitive, etc. Since this is not a review paper, we did not cover all possible voltage dyes.

One new dye approach was published by Miller, et al [48]. In that work, the technique to probe $\Delta\Psi m$ has been reported that utilizes photoinduced electron transfer (PeT)-based Rhodamine Voltage Reporter (RhoVR) instead of the traditional lipophilic dyes that accumulate in the mitochondria in a $\Delta\Psi m$-dependent manner [48]. In principle, such a technique could provide a faster voltage sensor, something that would be very exciting in the context of mitochondrial membrane potential fluctuations discussed in this manuscript. The issue of whether that dye would function in a super-resolution system has yet to be addressed. In addition, the low intensity and high pump powers needed would have to be addressed in the context of mitochondria. Miller et al also observed significant photobleaching, something we are also investigating actively (Burke, et al, manuscript in preparation).

We view this paper as the first step on a journey to dissect the electrophysiology of mitochondria on the nanoscale, not the final step. New dyes and new super-resolution imaging technologies are sorely needed. Ours is the first demonstrated quantitative super-resolution imaging of voltages in mitochondria, but we expect the field to explode now that we have laid the groundwork.

References

(1) Cafiso, D. S.; Hubbell, W. L. Estimation of Transmembrane Potentials from Phase Equilibriums of Hydrophobic Paramagnetic Ions. *Biochemistry* **1978**, *17* (1), 187–206.

(2) Demura, M.; Kamo, N.; Kobatake, Y. Mitochondrial Membrane Potential Estimated with the Correction of Probe Binding. *Biochimica et Biophysica Acta (BBA) - Bioenergetics* **1987**, *894* (3), 355–364. https://doi.org/10.1016/0005-2728(87)90113-7.

(3) Scaduto, R. C.; Grotyohann, L. W. Measurement of Mitochondrial Membrane Potential Using Fluorescent Rhodamine Derivatives. *Biophys J* **1999**, *76* (1), 469–477. https://doi.org/10.1016/S0006-3495(99)77214-0.

(4) Rottenberg, H. Membrane Potential and Surface Potential in Mitochondria: Uptake and Binding of Lipophilic Cations. *J Membr Biol* **1984**, *81* (2), 127–138. https://doi.org/10.1007/BF01868977.

(5) Callen, H. B.; Scott, H. L. Thermodynamics and an Introduction to Thermostatistics, 2nd Ed. *Am J Phys* **1998**, *66* (2), 164–167. https://doi.org/10.1119/1.19071.

(6) Nicholls, D. G.; Ferguson, S. J. *Bioenergetics*, 4th ed.; Academic Press, San Diego, 2013.

(7) Bradford, M. A Rapid and Sensitive Method for the Quantitation of Microgram Quantities of Protein Utilizing the Principle of Protein-Dye Binding. *Anal Biochem* **1976**, *72* (1–2), 248–254. https://doi.org/10.1006/abio.1976.9999.

(8) Olivia Prazeres da Costa; Ralf Engelmann; Martin Gleisner; Lutz Schäfer; Xianke Shi; Eva Simbürger; Max Voll; Klaus Weisshart; Georg Wieser. *A Practical Guide of Deconvolution*; 2021.

(9) Vicidomini, G.; Bianchini, P.; Diaspro, A. STED Super-Resolved Microscopy. *Nat Methods* **2018**, *15* (3), 173–182. https://doi.org/10.1038/nmeth.4593.

(10) Stephan, T.; Roesch, A.; Riedel, D.; Jakobs, S. Live-Cell STED Nanoscopy of Mitochondrial Cristae. *Sci Rep* **2019**, *9* (1), 12419. https://doi.org/10.1038/s41598-019-48838-2.

(11) Wang, C.; Taki, M.; Sato, Y.; Tamura, Y.; Yaginuma, H.; Okada, Y.; Yamaguchi, S. A Photostable Fluorescent Marker for the Superresolution Live Imaging of the Dynamic Structure of the Mitochondrial Cristae. *Proceedings of the National Academy of Sciences* **2019**, *116* (32), 15817–15822. https://doi.org/10.1073/pnas.1905924116.

(12) Song, Y.; Zhang, X.; Shen, Z.; Yang, W.; Wei, J.; Li, S.; Wang, X.; Li, X.; He, Q.; Zhang, S.; Zhang, Q.; Gao, B. Improving Brightness and Stability of Si-Rhodamine for Super-Resolution Imaging of Mitochondria in Living Cells. *Anal Chem* **2020**, *92* (18), 12137–12144. https://doi.org/10.1021/acs.analchem.9b04926.

(13) Yang, X.; Yang, Z.; Wu, Z.; He, Y.; Shan, C.; Chai, P.; Ma, C.; Tian, M.; Teng, J.; Jin, D.; Yan, W.; Das, P.; Qu, J.; Xi, P. Mitochondrial Dynamics Quantitatively Revealed by STED Nanoscopy with an Enhanced Squaraine Variant Probe. *Nat Commun* **2020**, *11* (1), 3699. https://doi.org/10.1038/s41467-020-17546-1.

(14) Gnaiger, E. *Mitochondrial Pathways and Respiratory Control. An Introduction to OXPHOS Analysis. Mitochondrial Physiology Network 17.18*; OROBOROS MiPNet Publications, Innsbruck, 2012.

(15) Lee, C.; Chen, Y.; Wang, P.; Wallace, D. C.; Burke, P. J. A Three-Dimensional Printed Inertial Microfluidic Platform for Isolation of Minute Quantities of Vital Mitochondria. *Anal Chem* **2022**, *94* (19), 6930–6938. https://doi.org/10.1021/acs.analchem.1c03244.

(16) Asin-Cayuela, J.; Manas, A.-R. B.; James, A. M.; Smith, R. A. J.; Murphy, M. P. Fine-Tuning the Hydrophobicity of a Mitochondria-Targeted Antioxidant. *FEBS Lett* **2004**, *571* (1–3), 9–16. https://doi.org/10.1016/j.febslet.2004.06.045.
(17) Prigione, A.; Fauler, B.; Lurz, R.; Lehrach, H.; Adjaye, J. The Senescence-Related Mitochondrial/Oxidative Stress Pathway Is Repressed in Human Induced Pluripotent Stem Cells. *Stem Cells* **2010**, *28* (4), 721–733. https://doi.org/10.1002/stem.404.
(18) Burke, P. J. Mitochondria, Bioenergetics and Apoptosis in Cancer. *Trends Cancer* **2017**, *3* (12), 857–870. https://doi.org/10.1016/j.trecan.2017.10.006.
(19) Gonzalez-Freire, M.; De Cabo, R.; Bernier, M.; Sollott, S. J.; Fabbri, E.; Navas, P.; Ferrucci, L. Reconsidering the Role of Mitochondria in Aging. *Journals of Gerontology - Series A Biological Sciences and Medical Sciences* **2015**, *70* (11), 1334–1342. https://doi.org/10.1093/gerona/glv070.
(20) Iovine, J. C.; Claypool, S. M.; Alder, N. N. Mitochondrial Compartmentalization: Emerging Themes in Structure and Function. *Trends Biochem Sci* **2021**, *46* (11), 902–917. https://doi.org/10.1016/j.tibs.2021.06.003.
(21) Cogliati, S.; Frezza, C.; Soriano, M. E.; Varanita, T.; Quintana-Cabrera, R.; Corrado, M.; Cipolat, S.; Costa, V.; Casarin, A.; Gomes, L. C.; Perales-Clemente, E.; Salviati, L.; Fernandez-Silva, P.; Enriquez, J. A.; Scorrano, L. Mitochondrial Cristae Shape Determines Respiratory Chain Supercomplexes Assembly and Respiratory Efficiency. *Cell* **2013**, *155* (1), 160–171. https://doi.org/10.1016/j.cell.2013.08.032.
(22) Glancy, B.; Kim, Y.; Katti, P.; Willingham, T. B. The Functional Impact of Mitochondrial Structure Across Subcellular Scales. *Front Physiol* **2020**, *11* (November), 1–24. https://doi.org/10.3389/fphys.2020.541040.
(23) Anand, R.; Reichert, A. S.; Kondadi, A. K. Emerging Roles of the MICOS Complex in Cristae Dynamics and Biogenesis. *Biology (Basel)* **2021**, *10* (7), 600. https://doi.org/10.3390/biology10070600.
(24) Patten, D. A.; Wong, J.; Khacho, M.; Soubannier, V.; Mailloux, R. J.; Pilon-Larose, K.; MacLaurin, J. G.; Park, D. S.; McBride, H. M.; Trinkle-Mulcahy, L.; Harper, M.; Germain, M.; Slack, R. S. OPA1-dependent Cristae Modulation Is Essential for Cellular Adaptation to Metabolic Demand. *EMBO J* **2014**, *33* (22), 2676–2691. https://doi.org/10.15252/embj.201488349.
(25) Hackenbrock, C. R. Ultrastructural Bases for Metabolically Linked Mechanical Activity in Mitochondria. I. Reversible Ultrastructural Changes with Change in Metabolic Steady State in Isolated Liver Mitochondria. *J Cell Biol* **1966**, *30* (2), 269–297. https://doi.org/10.1083/jcb.30.2.269.
(26) Hackenbrock, C. R. Ultrastructural Bases for Metabolically Linked Mechanical Activity in Mitochondria. II. Electron Transport-Linked Ultrastructural Transformations in Mitochondria. *J Cell Biol* **1968**, *37* (2), 345–369. https://doi.org/10.1083/jcb.37.2.345.
(27) Kondadi, A. K.; Anand, R.; Reichert, A. S. Cristae Membrane Dynamics – A Paradigm Change. *Trends Cell Biol* **2020**, *30* (12), 923–936. https://doi.org/10.1016/j.tcb.2020.08.008.
(28) Wolf, D. M.; Segawa, M.; Kondadi, A. K.; Anand, R.; Bailey, S. T.; Reichert, A. S.; Bliek, A. M.; Shackelford, D. B.; Liesa, M.; Shirihai, O. S. Individual Cristae within the Same Mitochondrion Display Different Membrane Potentials and Are Functionally Independent. *EMBO J* **2019**, *38* (22), e101056. https://doi.org/10.15252/embj.2018101056.

(29) Aryaman, J.; Johnston, I. G.; Jones, N. S. Mitochondrial Heterogeneity. *Front Genet* **2018**, *9* (JAN), 718. https://doi.org/10.3389/fgene.2018.00718.
(30) Nissanka, N.; Moraes, C. T. Mitochondrial DNA Heteroplasmy in Disease and Targeted Nuclease-Based Therapeutic Approaches. *EMBO Rep* **2020**, *21* (3), e49612. https://doi.org/10.15252/embr.201949612.
(31) Lim, T.-S.; Davila, A.; Wallace, D. C.; Burke, P. J. Assessment of Mitochondrial Membrane Potential Using an On-Chip Microelectrode in a Microfluidic Device. *Lab Chip* **2010**, *10*, 1683–1688.
(32) Lim, T.-S.; Davila, A.; Zand, K.; Wallace, D. C.; Burke, P. J. Wafer-Scale Mitochondrial Membrane Potential Assays. *Lab Chip* **2012**, *12* (15), 2719–2725. https://doi.org/10.1039/c2lc40086c.
(33) Burke, P. A Modified Hodgkin–Huxley Model for Nanoelectronics. In *International Conference On Nanomedicine And Nanobiotechnology*; Paris, France, 2016; p 167.
(34) Cafiso, D. S.; Hubbell, W. L. EPR Determination of Membrane Potentials. *Annu Rev Biophys Bioeng* **1981**, *10* (1), 217–244.
(35) Cafiso, D. S.; Hubbell, W. L. Transmembrane Electrical Currents of Spin-Labeled Hydrophobic Ions. *Biophys J* **1982**, *39* (3), 263–272. https://doi.org/10.1016/S0006-3495(82)84516-5.
(36) Cafiso, D. S.; Hubbell, W. L. Estimation of Transmembrane Potentials from Phase Equilibriums of Hydrophobic Paramagnetic Ions. *Biochemistry* **1978**, *17* (1), 187–195. https://doi.org/10.1021/bi00594a028.
(37) Kamo, N.; Muratsugu, M.; Hongoh, R.; Kobatake, Y. Membrane Potential of Mitochondria Measured with an Electrode Sensitive to Tetraphenyl Phosphonium and Relationship between Proton Electrochemical Potential and Phosphorylation Potential in Steady State. *Journal of Membrane Biology* **1979**, *49* (2), 105–121.
(38) O'Reilly, C. M.; Fogarty, K. E.; Drummond, R. M.; Tuft, R. A.; Walsh, J. V. Quantitative Analysis of Spontaneous Mitochondrial Depolarizations. *Biophys J* **2003**, *85* (5), 3350–3357.
(39) Labajova, A.; Vojtiskova, A.; Krivakova, P.; Kofranek, J.; Drahota, Z.; Houstek, J. Evaluation of Mitochondrial Membrane Potential Using a Computerized Device with a Tetraphenylphosphonium-Selective Electrode. *Anal Biochem* **2006**, *353* (1), 37–42. https://doi.org/10.1016/j.ab.2006.03.032.
(40) Gerencser, A. A.; Chinopoulos, C.; Birket, M. J.; Jastroch, M.; Vitelli, C.; Nicholls, D. G.; Brand, M. D. Quantitative Measurement of Mitochondrial Membrane Potential in Cultured Cells: Calcium-Induced de- and Hyperpolarization of Neuronal Mitochondria. *J Physiol* **2012**, *590* (Pt 12), 2845–2871. https://doi.org/10.1113/jphysiol.2012.228387.
(41) Nicholls, D. G. Fluorescence Measurement of Mitochondrial Membrane Potential Changes in Cultured Cells. *Methods Mol Biol* **2012**, *810*, 119–133. https://doi.org/10.1007/978-1-61779-382-0_8.
(42) Anna, L.; Yauheni, N.; Ralf, N.; Marie-Christine, S.; Ricardo, B.; Teresa, K.; Markus, S.; Ingo, K. Super-Resolution Imaging by Dual Iterative Structured Illumination Microscopy. *bioRxiv* **2021**.
(43) Liu, T.; Stephan, T.; Chen, P.; Keller-Findeisen, J.; Chen, J.; Riedel, D.; Yang, Z.; Jakobs, S.; Chen, Z. Multi-Color Live-Cell STED Nanoscopy of Mitochondria with a Gentle Inner Membrane Stain. *Proc Natl Acad Sci U S A* **2022**, *119* (52). https://doi.org/10.1073/pnas.2215799119.

(44) Stockhammer, A.; Bottanelli, F. Appreciating the Small Things in Life: STED Microscopy in Living Cells. *J Phys D Appl Phys* **2020**, *54* (3). https://doi.org/10.1088/1361-6463/abac81.
(45) Teodoro, J. S.; Palmeira, C. M.; Rolo, A. P. Mitochondrial Membrane Potential (ΔΨ) Fluctuations Associated with the Metabolic States of Mitochondria. *Methods Mol Biol* **2018**, *1782*, 109–119. https://doi.org/10.1007/978-1-4939-7831-1_6.
(46) Twig, G.; Elorza, A.; Molina, A. J. a; Mohamed, H.; Wikstrom, J. D.; Walzer, G.; Stiles, L.; Haigh, S. E.; Katz, S.; Las, G.; Alroy, J.; Wu, M.; Py, B. F.; Yuan, J.; Deeney, J. T.; Corkey, B. E.; Shirihai, O. S. Fission and Selective Fusion Govern Mitochondrial Segregation and Elimination by Autophagy. *EMBO J* **2008**, *27* (2), 433–446. https://doi.org/10.1038/sj.emboj.7601963.
(47) Wikstrom, J. D.; Katzman, S. M.; Mohamed, H.; Twig, G.; Graf, S. A.; Heart, E.; Molina, A. J. A.; Corkey, B. E.; de Vargas, L. M.; Danial, N. N. β-Cell Mitochondria Exhibit Membrane Potential Heterogeneity That Can Be Altered by Stimulatory or Toxic Fuel Levels. *Diabetes* **2007**, *56* (10), 2569.
(48) Klier, P. E. Z.; Martin, J. G.; Miller, E. W. Imaging Reversible Mitochondrial Membrane Potential Dynamics with a Masked Rhodamine Voltage Reporter. *J Am Chem Soc* **2021**, *143* (11), 4095–4099. https://doi.org/10.1021/jacs.0c13110.
